# Supplementary material for: Large Number of Direct or Pseudo-Direct Band Gap Semiconductors among A3TrPn2 Compounds with A = Li, Na, K, Rb, Cs; Tr = Al, Ga, In; Pn = P, As
Source: Molecules. 2024 Aug 28;29(17):4087. doi: 10.3390/molecules29174087 (PMC11397444; doi:10.3390/molecules29174087)
Supplement: Supplementary file 1 [file molecules-29-04087-s001.zip › molecules-3132256-supplementary.pdf]

Supporting Information: Large number of Direct or Pseudo-direct Band Gap Semiconductors Among A_3TrPn_2 Compounds with A = Li, Na, K, Rb, Cs; Tr = Al, Ga, In; Pn = P, As

Contents

1 Additional Information on DFT calculations **3**

2 Structure F **15**
2.1 Li_3AlP_2[1] 15
2.2 Li_3GaP_2[1] 18
2.3 Li_3AlAs_2[2] 21
2.4 Li_3GaAs_2[2] 24

3 Structure type G **27**
3.1 Li_3InP_2[3] 27
3.2 Li_3InAs_2[2] 30

4 Structure type E **33**
4.1 Na_3AlP_2[4] 33
4.2 Na_3GaP_2[5] 36
4.3 K_3InP_2[4] 39
4.4 Na_3AlAs_2[6] 41
4.5 K_3InAs_2[7] 44

5 Structure type H **46**
5.1 Na_3InP_2[8] 46
5.2 Na_3InAs_2[9] 50

6 Structure type C **54**
6.1 K_3AlP_2[10] 54
6.2 Rb_3InP_2[11] 59
6.3 K_3AlAs_2[12] 64

7 Structure type D **69**

8 Structure type B **74**

9 Structure type A **78**
9.1 Cs_3AlP_2[15] 78
9.2 Cs_3GaP_2[16] 82
9.3 Cs_3AlAs_2[17] 86
9.4 Cs_3GaAs_2[18] 89

10 Structure predictions **92**
10.1 K_3GaP_2 92
10.2 Rb_3AlP_2 96
10.3 Na_3GaAs_2 100
10.4 K_3GaAs_2 102
10.5 Rb_3AlAs_2 106
10.6 Rb_3GaAs_2 111
10.7 Rb_3InAs_2 115
10.8 Cs_3InAs_2 118

References **123**

1 Additional Information on DFT calculations

Table S1. Optimized atomic coordinates for all 3-1-2 compounds, including the lowest energy structure models for all predicted compounds (written in italics). All atomic positions have an occupation of 1.

atom	Wyckoff position	x	y	z
		Li_3AlP_2		
Li1	8 d	0.11487	0.00000	0.00000
Li2	16 g	0.62386	0.25874	0.02222
Al1	8 d	0.36774	0.00000	0.00000
P1	8 f	0.00000	0.38201	0.21016
P2	8 e	0.25000	0.11603	0.25000
		Li_3GaP_2		
Li1	16 g	0.87746	0.24166	-0.01907
Li2	8 d	0.11557	0.00000	0.00000
Ga1	8 d	0.36647	0.00000	0.00000
P1	8 f	0.00000	0.38217	0.21212
P2	8 e	0.25000	0.11833	0.25000
		Li_3AlAs_2		
Li1	8 d	0.38413	0.00000	0.00000
Li2	16 g	0.37642	0.24179	0.01948
Al1	8 d	0.13214	0.00000	0.00000
As1	8 f	0.00000	0.11884	0.21286
As2	8 e	0.25000	0.88215	0.25000
		Li_3GaAs_2		
Li1	8 d	0.38344	0.00000	0.00000
Li2	16 g	0.62198	0.24181	0.01635
Ga1	8 d	0.13384	0.00000	0.00000
As1	8 f	0.00000	0.11812	0.21347
As2	8 e	0.25000	0.87990	0.25000
		Li_3InP_2		
Li1	32 g	0.12102	0.37868	0.18880
Li2	32 g	0.87349	0.36753	0.18505
Li3	32 g	0.87121	0.61326	0.05955
In1	32 g	0.37519	0.86733	0.43633
P1	16 d	0.00000	0.25000	0.24948
P2	16 e	0.23844	0.00000	0.25000

Table S1. Continued.

atom	Wyckoff position	x	y	z
P3	32 g	0.00613	0.50041	0.12487
		Li_3InAs_2		
Li1	32 g	0.87217	0.36908	0.18551
Li2	32 g	0.87256	0.61468	0.06027
Li3	32 g	0.62912	0.62800	0.06052
In1	32 g	0.37527	0.86712	0.43621
As1	16 d	0.00000	0.25000	0.24900
As2	16 e	0.23978	0.00000	0.25000
As3	32 g	0.00646	0.49902	0.12498
		Na_3AlP_2		
Na1	8 j	0.21038	0.81344	0.00000
Na2	4 b	0.50000	0.00000	0.25000
Al1	4 a	0.00000	0.00000	0.25000
P1	8 j	0.70126	0.39557	0.00000
		Na_3GaP_2		
Na1	4 b	0.50000	0.00000	0.25000
Na2	8 j	0.77977	0.18433	0.00000
Ga1	4 a	0.00000	0.00000	0.25000
P1	8 j	0.70319	0.39420	0.00000
		K_3InP_2		
K1	8 j	0.20975	0.81753	0.00000
K2	4 b	0.50000	0.00000	0.25000
In1	4 a	0.00000	0.00000	0.25000
P1	8 j	0.69218	0.40139	0.00000
		Na_3AlAs_2		
Na1	8 j	0.23095	0.81793	0.00000
Na2	4 b	0.50000	0.00000	0.25000
Al1	4 a	0.00000	0.00000	0.25000
As1	8 j	0.70468	0.39184	0.00000
		K_3InAs_2		
K1	8 j	0.82049	0.21406	0.00000
K2	4 b	0.50000	0.00000	0.25000
In1	4 a	0.00000	0.00000	0.25000

Table S1. Continued.

atom	Wyckoff position	x	y	z
As1	8 j	0.60002	0.30457	0.00000
		Cs_3InAs_2		
Cs1	8 j	0.82255	0.20656	0.00000
Cs2	4 b	0.50000	0.00000	0.25000
In1	4 a	0.00000	0.00000	0.25000
As1	8 j	0.59109	0.32301	0.00000
		Na_3InP_2		
Na1	4 e	0.87589	0.34819	0.79719
Na2	4 e	0.55872	0.00999	0.39570
Na3	4 e	0.18698	0.53712	0.85006
Na4	4 e	0.38351	0.65299	0.45438
Na5	4 e	-0.06617	0.52312	0.60671
Na6	4 e	0.31995	0.49752	0.63738
In1	4 e	0.63615	0.65553	0.71050
In2	4 e	0.87345	0.64203	-0.04101
P1	4 e	0.65164	0.73736	0.54179
P2	4 e	0.87586	0.74209	0.79360
P3	4 e	0.40573	0.70660	0.27451
P4	4 e	0.87291	0.21904	0.47666
		Na_3InAs_2		
Na1	4 e	0.87373	0.34836	0.79792
Na2	4 e	0.55902	0.01008	0.39527
Na3	4 e	0.18748	0.53609	0.85080
Na4	4 e	0.38427	0.65233	0.45389
Na5	4 e	-0.06681	0.52649	0.60718
Na6	4 e	0.32083	0.49389	0.63779
In1	4 e	0.63795	0.65412	0.70981
In2	4 e	0.87471	0.64177	-0.04152
As1	4 e	0.65095	0.73819	0.53996
As2	4 e	0.87928	0.74375	0.79218
As3	4 e	0.40590	0.71110	0.27424
As4	4 e	0.87045	0.22217	0.47732
		Na_3GaAs_2		
Na1	4 e	0.87667	0.34390	0.79598
Na2	4 e	0.56557	0.00183	0.39514
Na3	4 e	0.18501	0.53049	0.84812

Table S1. Continued.

atom	Wyckoff position	x	y	z
Na4	4 e	0.38349	0.65580	0.45435
Na5	4 e	-0.06950	0.52403	0.60698
Na6	4 e	0.32722	0.49980	0.63565
Ga1	4 e	0.63673	0.65883	0.71244
Ga2	4 e	0.87392	0.63657	-0.03872
As1	4 e	0.65769	0.73850	0.54611
As2	4 e	0.86811	0.73549	0.79863
As3	4 e	0.41225	0.69824	0.27269
As4	4 e	0.87872	0.20954	0.47922
		K_3AlP_2		
K1	2 i	0.34558	-0.08115	0.69180
K2	2 i	0.82634	0.39986	0.69321
K3	2 i	0.80244	0.76312	0.68238
K4	2 i	0.21691	0.75216	0.61891
K5	2 i	0.16706	0.42827	0.75606
K6	2 i	-0.01658	0.10706	0.58696
K7	2 i	0.54658	0.07908	0.74647
K8	2 i	0.48606	0.57751	0.83987
K9	2 i	0.03545	0.10054	0.84691
K10	2 i	0.52294	0.37598	0.58472
K11	2 i	0.31545	0.08536	0.01544
K12	2 i	0.81889	0.62412	-0.02605
Al1	2 i	0.15972	0.38926	0.51019
Al2	2 i	0.40932	0.14349	0.50894
Al3	2 i	0.14690	0.78171	-0.07845
Al4	2 i	0.64115	0.26919	-0.05377
P1	2 i	0.22352	0.22104	0.64301
P2	2 i	0.32782	-0.01328	0.46147
P3	2 i	0.58660	0.65947	0.60482
P4	2 i	0.08782	0.59200	0.54174
P5	2 i	-0.01329	0.83736	0.81643
P6	2 i	0.48872	0.29691	0.84314
P7	2 i	0.85221	0.36330	-0.06714
P8	2 i	0.37208	0.86367	-0.09455
		Rb_3InP_2		
In1	2 i	0.58667	0.35494	-0.00840
In2	2 i	0.83755	0.10925	-0.01130
In3	2 i	0.13885	0.28586	0.41968

Table S1. Continued.

atom	Wyckoff position	x	y	z
In4	2 i	0.36689	0.22938	0.55632
Rb1	2 i	0.65703	0.57529	0.80632
Rb2	2 i	0.78616	0.74773	0.87917
Rb3	2 i	0.17656	0.09707	0.80524
Rb4	2 i	0.48034	0.08038	0.34349
Rb5	2 i	0.79501	0.26718	0.18220
Rb6	2 i	0.01333	0.39611	-0.09043
Rb7	2 i	-0.03660	0.39652	0.64729
Rb8	2 i	0.45627	0.41428	0.75169
Rb9	2 i	0.83765	0.06818	0.74379
Rb10	2 i	0.47125	0.12384	-0.08557
Rb11	2 i	0.32151	0.58944	0.51691
Rb12	2 i	0.16911	0.87832	0.52798
P1	2 i	0.57862	0.15626	0.10725
P2	2 i	0.77505	0.27722	0.85293
P3	2 i	0.32611	0.48628	-0.03854
P4	2 i	-0.08272	-0.09720	-0.04171
P5	2 i	0.02216	0.65238	0.68708
P6	2 i	0.84836	0.86589	0.43432
P7	2 i	0.63435	0.62820	0.59220
P8	2 i	0.46943	0.80846	0.34271
		K_3AlAs_2		
K1	2 i	0.34487	-0.07854	0.68954
K2	2 i	0.83030	0.40025	0.69257
K3	2 i	0.79466	0.75873	0.68495
K4	2 i	0.20647	0.75994	0.61893
K5	2 i	0.17288	0.43255	0.75426
K6	2 i	-0.01761	0.10862	0.58889
K7	2 i	0.56130	0.07217	0.74784
K8	2 i	0.48173	0.57618	0.84272
K9	2 i	0.03011	0.10263	0.84646
K10	2 i	0.52833	0.37330	0.58327
K11	2 i	0.31334	0.08541	0.00646
K12	2 i	0.81382	0.63035	-0.02763
Al1	2 i	0.16315	0.38970	0.51013
Al2	2 i	0.41168	0.14495	0.50857
Al3	2 i	0.14296	0.78506	-0.08205
Al4	2 i	0.64391	0.27117	-0.05278
As1	2 i	0.22687	0.22171	0.64631

Table S1. Continued.

atom	Wyckoff position	x	y	z
As2	2 i	0.32184	-0.00986	0.46051
As3	2 i	0.57715	0.65561	0.60748
As4	2 i	0.08668	0.59487	0.54286
As5	2 i	-0.02685	0.84924	0.81326
As6	2 i	0.49417	0.29500	0.84044
As7	2 i	0.86043	0.36511	-0.06532
As8	2 i	0.38112	0.85983	-0.09951
		Cs_3InP_2		
Cs1	2 i	0.04010	0.48112	0.20060
Cs2	2 i	0.40993	-0.01595	0.81204
Cs3	2 i	0.40425	0.33658	0.82417
Cs4	2 i	0.15041	0.23473	0.67275
Cs5	2 i	0.53769	0.64517	-0.08829
Cs6	2 i	0.78352	0.03688	0.74969
Cs7	2 i	0.21479	0.69279	0.12139
Cs8	2 i	0.11038	0.83331	0.48756
Cs9	2 i	0.27366	0.22812	0.35667
Cs10	2 i	0.47205	0.36893	0.54796
Cs11	2 i	0.24888	0.67199	0.72864
Cs12	2 i	0.06083	0.89843	-0.08509
In1	2 i	-0.08446	0.64428	-0.00902
In2	2 i	0.66254	0.89363	-0.01267
In3	2 i	0.35857	-0.02161	0.56159
In4	2 i	0.86132	0.48926	0.57350
P1	2 i	0.39201	0.89023	0.04053
P2	2 i	0.46693	-0.08301	0.41485
P3	2 i	0.85596	0.79205	0.09928
P4	2 i	0.19099	0.52902	-0.03829
P5	2 i	0.79892	0.78275	0.85839
P6	2 i	0.09010	0.54950	0.58231
P7	2 i	0.14573	-0.03732	0.65776
P8	2 i	0.64861	0.49930	0.67858
		Rb_3InAs_2		
Rb1	2 i	0.53438	0.57262	0.19225
Rb2	2 i	0.33009	0.74297	0.12212
Rb3	2 i	0.02082	0.09655	0.19473
Rb4	2 i	0.17498	0.08028	0.65203
Rb5	2 i	0.02738	0.26529	0.81602

Table S1. Continued.

atom	Wyckoff position	x	y	z
Rb6	2 i	0.07816	0.39433	0.09147
Rb7	2 i	0.38894	0.39495	0.35486
Rb8	2 i	0.80136	0.41827	0.24927
Rb9	2 i	0.41989	0.06285	0.25389
Rb10	2 i	0.61824	0.12753	0.08358
Rb11	2 i	0.16448	0.58968	0.48785
Rb12	2 i	0.30335	0.87460	0.47272
In1	2 i	0.42192	0.35363	0.00747
In2	2 i	0.17616	0.10845	0.01101
In3	2 i	0.44585	0.28824	0.58256
In4	2 i	0.07529	0.22751	0.44310
As1	2 i	0.31899	0.15321	0.89031
As2	2 i	0.37673	0.27582	0.14930
As3	2 i	0.71592	0.48721	0.03893
As4	2 i	0.12286	0.89993	0.04223
As5	2 i	0.28167	0.64481	0.31206
As6	2 i	0.71368	0.86837	0.56357
As7	2 i	0.77702	0.63050	0.40560
As8	2 i	0.18850	0.80855	0.66072
		Rb_3GaP_2		
Rb1	8 c	0.68345	0.32883	0.20953
Rb2	8 c	0.67262	0.43022	0.78729
Rb3	8 c	0.53969	0.29224	0.89533
Rb4	8 c	0.59540	0.47200	0.35953
Rb5	8 c	0.82868	0.27601	0.86082
Rb6	8 c	0.84528	0.41225	0.47069
Ga1	8 c	0.54491	0.34147	0.52542
Ga2	8 c	0.87826	0.40153	0.05366
P1	8 c	-0.04445	0.33451	0.19257
P2	8 c	0.68124	0.19382	0.07868
P3	8 c	0.45589	0.39673	0.67448
P4	8 c	0.76488	0.45539	0.11996
		K_3GaP_2		
K1	8 c	0.68895	0.32757	0.21562
K2	8 c	0.67751	0.43036	0.78375
K3	8 c	0.54747	0.29416	0.89988
K4	8 c	0.59765	0.47102	0.36278
K5	8 c	0.83579	0.27615	0.86196

Table S1. Continued.

atom	Wyckoff position	x	y	z
K6	8 c	0.84608	0.41342	0.46976
Ga1	8 c	0.55113	0.34059	0.52781
Ga2	8 c	0.87921	0.40175	0.05340
P1	8 c	-0.03930	0.33285	0.20003
P2	8 c	0.69171	0.19521	0.08277
P3	8 c	0.46017	0.39871	0.68363
P4	8 c	0.75825	0.45521	0.11831
		Rb_3AlP_2		
Rb1	8 c	0.68215	0.32925	0.20663
Rb2	8 c	0.67373	0.43007	0.78617
Rb3	8 c	0.53858	0.29226	0.89469
Rb4	8 c	0.59597	0.47256	0.35483
Rb5	8 c	0.82576	0.27511	0.85745
Rb6	8 c	0.84427	0.41224	0.46959
Al1	8 c	0.54158	0.34207	0.52444
Al2	8 c	0.87956	0.40086	0.05161
P1	8 c	-0.04585	0.33454	0.19269
P2	8 c	0.67771	0.19377	0.07559
P3	8 c	0.45446	0.39633	0.67644
P4	8 c	0.76691	0.45510	0.11756
		K_3GaAs_2		
K1	8 c	0.69193	0.32748	0.21859
K2	8 c	0.68059	0.42901	0.78358
K3	8 c	0.55039	0.29416	-0.09914
K4	8 c	0.59749	0.46931	0.37200
K5	8 c	0.83923	0.27684	0.86843
K6	8 c	0.84712	0.41519	0.46751
Ga1	8 c	0.55283	0.33998	0.52911
Ga2	8 c	0.87907	0.40236	0.05388
As1	8 c	-0.03829	0.33220	0.20311
As2	8 c	0.69680	0.19518	0.08564
As3	8 c	0.46112	0.39995	0.68697
As4	8 c	0.75517	0.45633	0.11976
		Rb_3AlAs_2		
Rb1	8 c	0.68595	0.32887	0.20967
Rb2	8 c	0.67710	0.42891	0.78630
Rb3	8 c	0.54217	0.29241	0.89642

Table S1. Continued.

atom	Wyckoff position	x	y	z
Rb4	8 c	0.59522	0.47126	0.36128
Rb5	8 c	0.83044	0.27572	0.86299
Rb6	8 c	0.84506	0.41385	0.46854
Al1	8 c	0.54404	0.34188	0.52526
Al2	8 c	0.88031	0.40118	0.05196
As1	8 c	-0.04426	0.33365	0.19654
As2	8 c	0.68337	0.19391	0.07854
As3	8 c	0.45604	0.39756	0.68053
As4	8 c	0.76476	0.45606	0.11903
		Rb_3GaAs_2		
Rb1	8 c	0.68737	0.32851	0.21250
Rb2	8 c	0.67647	0.42894	0.78780
Rb3	8 c	0.54347	0.29255	0.89730
Rb4	8 c	0.59492	0.47055	0.36675
Rb5	8 c	0.83345	0.27655	0.86591
Rb6	8 c	0.84671	0.41378	0.46980
Ga1	8 c	0.54781	0.34104	0.52616
Ga2	8 c	0.87880	0.40186	0.05443
As1	8 c	-0.04258	0.33370	0.19595
As2	8 c	0.68707	0.19401	0.08150
As3	8 c	0.45779	0.39792	0.67733
As4	8 c	0.76282	0.45631	0.12183
		Cs_3AlP_2		
Cs1	4 e	0.08491	0.57201	0.74002
Cs2	4 e	0.35681	0.24309	0.79002
Cs3	4 e	0.73483	0.41055	0.57828
Cs4	4 e	0.37592	0.43080	0.61344
Cs5	4 e	0.29526	0.88892	0.66096
Cs6	4 e	0.85284	0.53289	0.03518
Al1	4 e	0.46335	0.55423	-0.07302
Al2	4 e	0.04209	0.38742	0.55546
P1	4 e	-0.09854	0.71412	0.85763
P2	4 e	0.40532	0.63822	0.81347
P3	4 e	0.48545	0.29670	-0.03122
P4	4 e	0.06812	0.65609	0.55937
		Cs_3GaP_2		
Cs1	4 e	0.37633	0.57117	0.61391

Table S1. Continued.

atom	Wyckoff position	x	y	z
Cs2	4 e	0.29658	0.11125	0.65959
Cs3	4 e	0.14722	-0.03416	0.46457
Cs4	4 e	0.26512	0.08981	-0.07658
Cs5	4 e	0.08435	0.42754	0.73858
Cs6	4 e	0.35735	0.75712	0.79174
Ga1	4 e	0.04404	0.61565	0.55741
Ga2	4 e	0.46111	0.44473	-0.07521
P1	4 e	0.10122	0.78984	0.64423
P2	4 e	-0.06658	0.65565	0.44133
P3	4 e	0.48360	0.70237	-0.03035
P4	4 e	0.40351	0.36016	0.81104
		Cs_3AlAs_2		
Cs1	4 e	0.08573	0.56913	0.73996
Cs2	4 e	0.35580	0.24249	0.78978
Cs3	4 e	0.73678	0.41160	0.57848
Cs4	4 e	0.37495	0.43154	0.61392
Cs5	4 e	0.29931	0.89036	0.66132
Cs6	4 e	0.14932	0.46828	-0.03355
Al1	4 e	0.46271	0.55461	-0.07337
Al2	4 e	0.04296	0.38669	0.55556
As1	4 e	-0.09954	0.70934	0.85644
As2	4 e	0.40415	0.63994	0.81126
As3	4 e	0.06959	0.66069	0.56041
As4	4 e	0.48231	0.29094	-0.03158
		Cs_3GaAs_2		
Cs1	4 e	0.37540	0.57081	0.61441
Cs2	4 e	0.30037	0.11059	0.65997
Cs3	4 e	0.15000	-0.03276	0.46678
Cs4	4 e	0.26251	0.08897	-0.07714
Cs5	4 e	0.08501	0.43121	0.73849
Cs6	4 e	0.35592	0.75793	0.79212
Ga1	4 e	0.04518	0.61670	0.55772
Ga2	4 e	0.46035	0.44433	-0.07589
As1	4 e	0.10179	0.79540	0.64521
As2	4 e	-0.06661	0.65950	0.44017
As3	4 e	0.48187	0.70721	-0.03048
As4	4 e	0.40274	0.35892	0.80860

Table S2. Calculated band gaps and transitions as well as an overview of the sampled reciprocal space defined by the Monkhorst-Pack-type *k*-point grid (SHRINK) and Brillouin Zone paths for all 3-1-2 compounds.

compound	band gap		transition	k-path	SHRINK
Li_3AlP_2	3.06	direct	Γ -> Γ	Γ-Y-T-Z-R-S-Γ|Σ_0-Γ-Z-A_0	4 4 6
Li_3GaP_2	2.83	direct	Γ -> Γ	-"-	-"-
Li_3AlAs_2	2.85	direct	Γ -> Γ	Γ—Y|S_0—Γ—Z—A_0|T—Y|Γ—S—R—Z—T	3 3 6
Li_3GaAs_2	2.43	direct	Γ -> Γ	-"-	4 4 5
Li_3InP_2	2.69	direct	Γ -> Γ	Γ-X-P-N-Γ-M-S|S_0-Γ|G-M	3 3 3
Li_3InAs_2	2.26	direct	Γ -> Γ	Γ—X—P—N—Γ—M—S|S_0—Γ|X—R|G—M	2 2 2
Na_3AlP_2	3.34	indirect	X-Γ -> G_0-X	Σ_0-Γ-Λ_0|G_0-X-Γ-R-W-S-Γ-T-W	4 4 4
Na_3GaP_2	2.88	indirect	Γ -> G_0-X	G_0-X-Γ-R-W-S-Γ-T-W	-"-
K_3InP_2	2.89	direct	Γ -> Γ	Γ—X—F_0|S_0—Γ—Λ_0|G_0—X|Γ—R—W—S—Γ—T—W	10 10 10
Na_3AlAs_2	2.98	indirect	Γ -> G0-X	-"-	4 4 4
K_3InAs_2	2.69	direct	Γ -> Γ	-"-	-"-
Cs_3InAs_2	2.24	direct	Γ -> Γ	-"-	10 10 10
Na_3InP_2	2.09	direct	Γ -> Γ	Γ—Z—D—B—Γ—A—E—Z—C_2—Y_2—Γ	4 5 2
Na_3InAs_2	1.69	direct	Γ -> Γ	-"-	-"-
Na_3GaAs_2	2.03	direct	Γ -> Γ	-"-	-"-
K_3AlP_2	2.42	direct	Γ -> Γ	Γ—X|Y—Γ—Z|R—Γ—T|U—Γ—V	8 6 4
Rb_3InP_2	1.73	direct	Γ -> Γ	-"-	-"-
K_3AlAs_2	2.27	direct	Γ -> Γ	-"-	-"-
Cs_3InP_2	1.99	direct	Γ -> Γ	Γ—X|Y—Γ—Z|R—Γ—T|U—Γ—V	8 6 4
Rb_3GaP_2	2.41	direct	Γ -> Γ	Γ—X—S—Y—Γ—Z—U—R—T—Z|X—U|Y—T|S—R	6 4 8
K_3GaP_2	2.98	direct	Γ -> Γ	-"-	-"-
Rb_3AlP_2	2.44	direct	Γ -> Γ	-"-	-"-
K_3GaAs_2	2.79	direct	Γ -> Γ	-"-	-"-
Rb_3AlAs_2	2.26	direct	Γ -> Γ	-"-	-"-
Rb_3GaAs_2	2.23	direct	Γ -> Γ	-"-	-"-
Cs_3AlP_2	2.54	pseudo-direct	Γ -> Γ	Γ—Z—D—B—Γ—A—E—Z—C_2—Y_2—Γ	6 8 4
Cs_3GaP_2	2.54	pseudo-direct	Γ -> Γ	-"-	-"-
Cs_3AlAs_2	2.26	pseudo-direct	Γ -> Γ	-"-	-"-
Cs_3GaAs_2	2.35	pseudo-direct	Γ -> Γ	-"-	-"-
Rb_3InAs_2	1.57	direct	Γ -> Γ	-"-	-"-

Table S3. (Extended version of Table 2) Energy comparison of different possible structures for all unknown phosphides and arsenides. The structure type (str. type) in the second column refers to the crystal structures shown in Figure 1. The energetically most stable structure was set to 0. The type of band gap is marked as follows: dir. = direct, indir. = indirect, p-dir. = pseudo-direct band gap (where the indirect band gap is less than 0.03 eV smaller than the direct).

	str. type	E/Z (AU)	ΔΔE (kJ/mol)	G/Z (AU)	ΔΔG (kJ/mol)	band gap (eV)
K_3GaP_2	**B**	-4406.360	0	-4406.366	0	2.98 (dir.)
	C	-4406.357	6.01	-4406.363	8.24	2.41 (dir.)
	E	-4406.353	18.15	-4406.356	25.72	3.12 (p-dir.)
Rb_3AlP_2	**B**	-997.300	0	-997.3090	0	2.44 (dir.)
	C	-997.299	4.32	-997.3066	6.39	1.85 (dir.)
	A	-997.298	6.21	-997.3068	5.87	2.37 (p-dir.)
Na_3GaAs_2	**H**	-6882.546	0	-6882.552	0	2.03 (dir.)
	F	-6882.540	17.02	-6882.544	20.09	2.30 (dir.)
	E	-6882.542	11.423	-6882.547	11.415	2.39 (indir.)
K_3GaAs_2	**B**	-8195.153	0	-8195.163	0	2.79 (dir.)
	C	-8195.151	5.41	-8195.160	7.22	2.17 (dir.)
	E	-8195.147	14.48	-8195.154	22.09	2.96 (indir.)
Rb_3AlAs_2	**B**	-4786.092	0	-4786.104	0	2.26 (dir.)
	C	-4786.091	3.44	-4786.102	4.88	1.73 (dir.)
	A	-4786.089	7.29	-4786.102	7.11	2.18 (dir.)
Rb_3GaAs_2	**B**	-6468.375	0	-6468.388	0	2.23 (dir.)
	A	-6468.372	7.94	-6468.386	7.44	2.15 (indir.)
	C	-6468.370	13.53	-6468.383	14.80	1.67 (dir.)
Rb_3InAs_2	***C***	-4733.9034	0	-4733.918	0.34	1.57 (dir.)
	A	-4733.9030	1.00	-4733.918	0	1.86 (indir.)
	E	-4733.9018	4.19	-4733.914	11.86	1.96 (indir.)
Cs_3InAs_2	**E**	-4722.079	0	-4722.097	0	2.24 (dir.)
	D	-4722.078	3.26	-4722.094	6.32	1.81 (dir.)
	A	-4722.076	7.09	-4722.094	7.01	2.15 (dir.)
	E	-4722.075	9.78	-4722.089	20.42	2.07 (indir.)

2 Structure F

2.1 Li_3AlP_2[1]

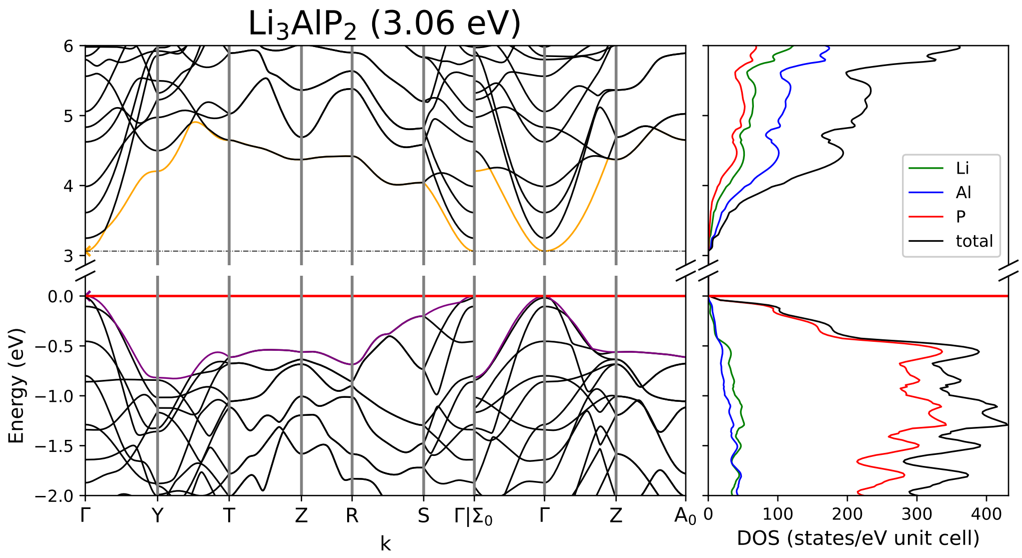

Figure S1. Band structure and DOS of Li_3AlP_2.

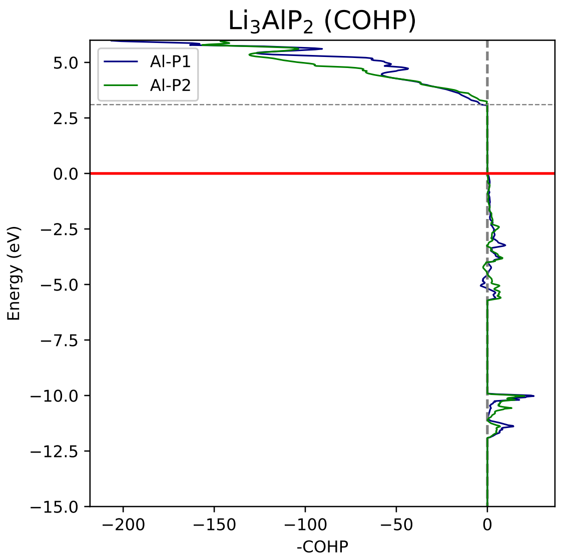

Figure S2. COHP of Al-P interactions in Li_3AlP_2.

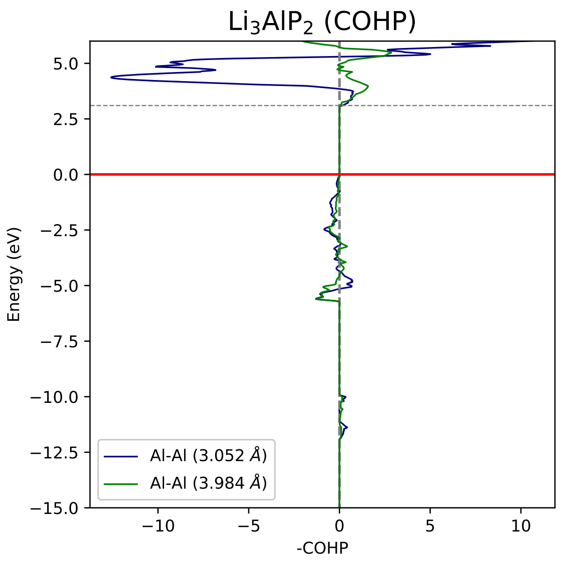

Figure S3. COHP of Al-Al interactions in Li_3AlP_2.

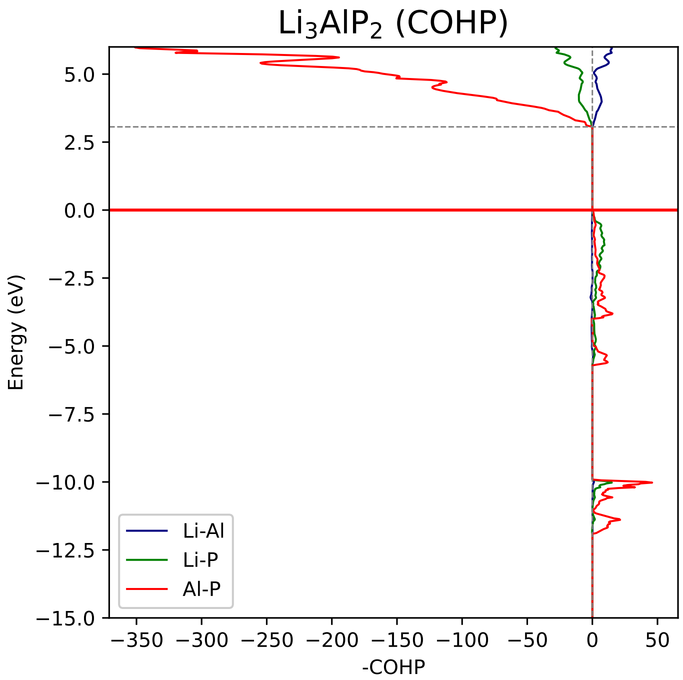

Figure S4. COHP of Li-Al, Li-P and Al-P interactions in Li_3AlP_2.

Table S4. Overlap population and interatomic distances of Li_3AlP_2.

Atom A	Atom B	r_{AB} / Å	overlap
Li1	P2	2.533	0.059
	P1	2.556	0.067
	Li1	2.651	0.01
	Li2	2.841	0.007
	Al1	2.918	0.013
	Al1	2.92	0.013
	Li2	3.956	0.001
Li2	P1	2.564	0.071
	P1	2.572	0.067
	P2	2.587	0.062
	P2	2.608	0.076
	Li2	2.858	0.007
	Li2	2.921	0.006
	Li2	2.93	0.006
	Al1	3.046	0.012
	Li2	3.939	0.001

Atom A	Atom B	r_{AB} / Å	overlap
Al1	P1	2.398	0.283
	P2	2.414	0.289
	Al1	3.052	−0.023
	Al1	3.984	−0.017
P1	P1	3.7	−0.056
	P2	3.938	−0.03
	P2	3.993	−0.033
	P1	4.257	−0.005
	P2	4.261	−0.004
	P2	4.268	−0.005
P2	P2	3.991	−0.034
	P2	4.291	−0.005

Table S5. Partial charges for each atom position in Li_3AlP_2.

Atom	Z	charge	part charge
Li1	3	2.423	0.577
Li2		2.426	0.574
Al1	13	12.655	0.345

Atom	Z	charge	partialcharge
P1	15	16.052	−1.052
P2		16.018	−1.018

2.2 Li_3GaP_2[1]

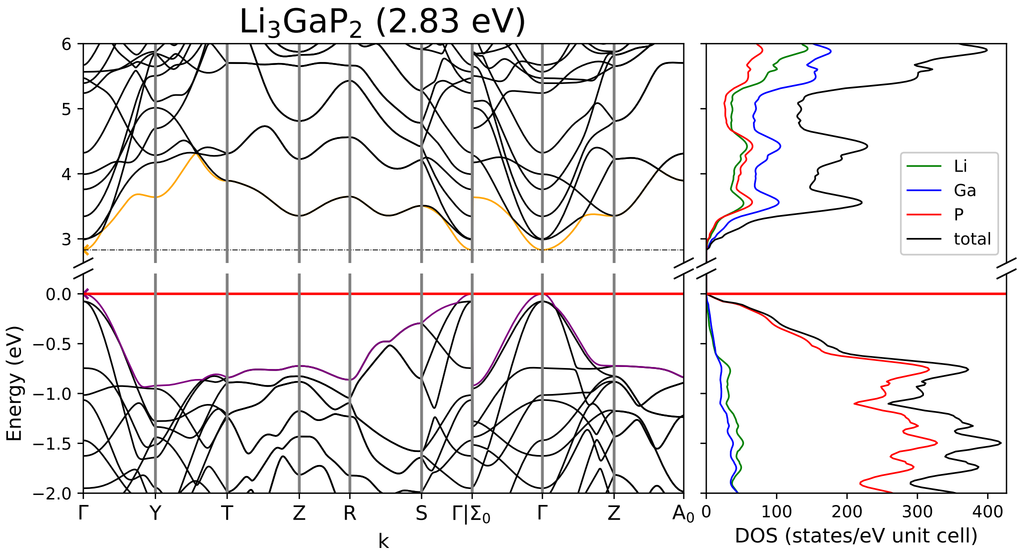

Figure S5. Band structure and DOS of Li_3GaP_2.

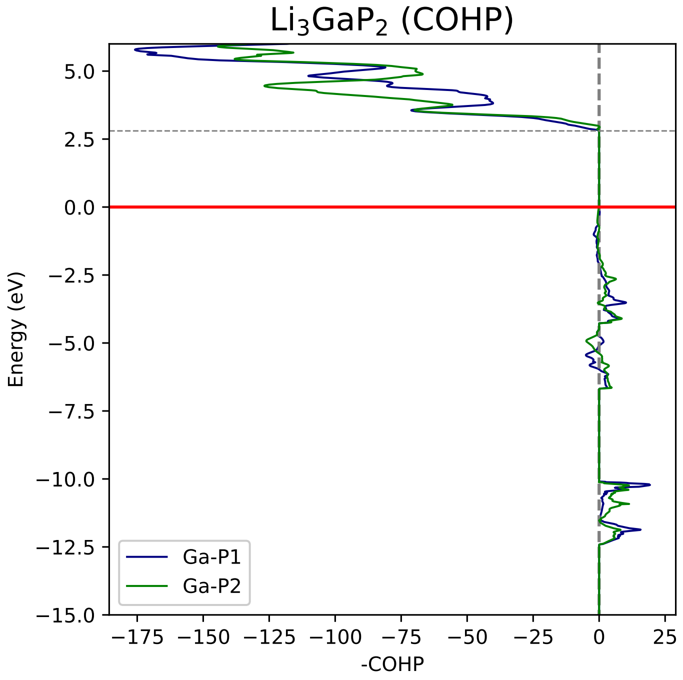

Figure S6. COHP of Ga-P interactions in Li_3GaP_2.

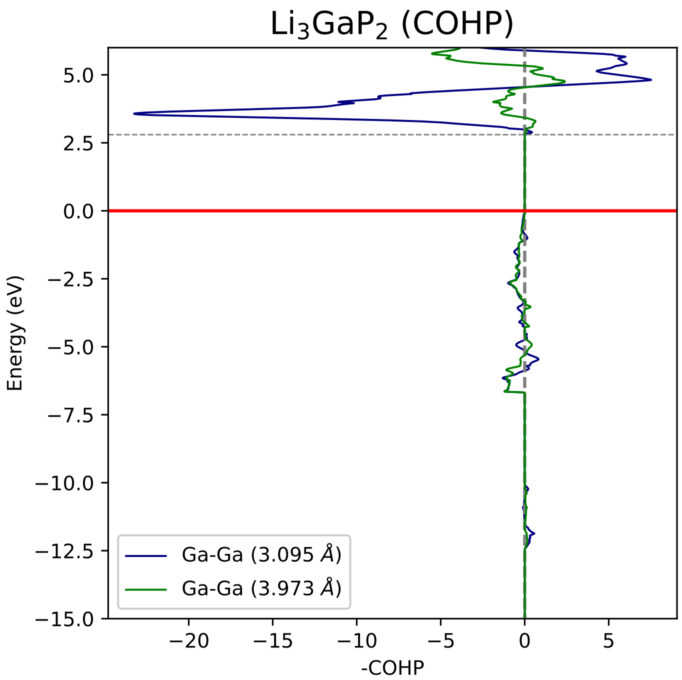

Figure S7. COHP of Ga-Ga interactions in Li_3GaP_2.

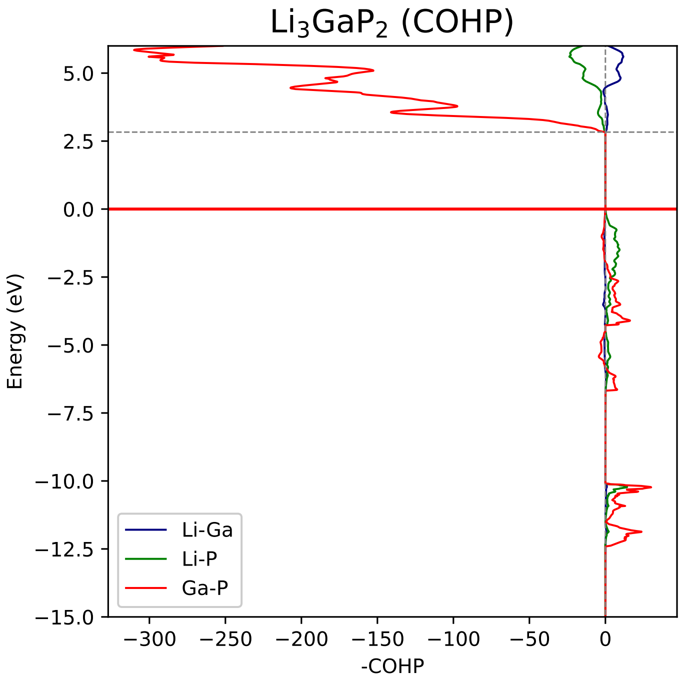

Figure S8. COHP of Li-Ga, Li-P and Ga-P interactions in Li_3GaP_2.

Table S6. Partial charges for each atom position in Li_3GaP_2.

Atom	Z	charge	part charge
Li1	3	2.442	0.558
Li2		2.427	0.573
Ga1	31	30.895	0.105

Atom	Z	charge	partialcharge
P1	15	15.913	−0.913
P2		15.881	−0.881

Table S7. Overlap population and interatomic distances of Li_3GaP_2.

Atom A	Atom B	r_{AB} / Å	overlap
Li1	P1	2.564	0.07
	P1	2.569	0.073
	P2	2.592	0.061
	P2	2.599	0.081
	Li1	2.841	0.008
	Li2	2.851	0.007
	Li1	2.921	0.006
	Li1	2.97	0.006
	Ga1	3.049	0.012
	Li2	3.967	0.0
Li2	P2	2.549	0.058
	P1	2.557	0.07
	Li2	2.679	0.01
	Ga1	2.908	0.012
	Ga1	2.922	0.013

Atom A	Atom B	r_{AB} / Å	overlap
Ga1	P1	2.419	0.248
	P2	2.427	0.255
	Ga1	3.095	−0.055
	Ga1	3.973	−0.035
P1	P1	3.718	−0.04
	P2	3.956	−0.027
	P2	4.024	−0.03
	P2	4.256	−0.005
	P1	4.266	−0.006
	P2	4.269	−0.006
P2	P2	4.034	−0.03
	P2	4.257	−0.005

2.3 Li_3AlAs_2[2]

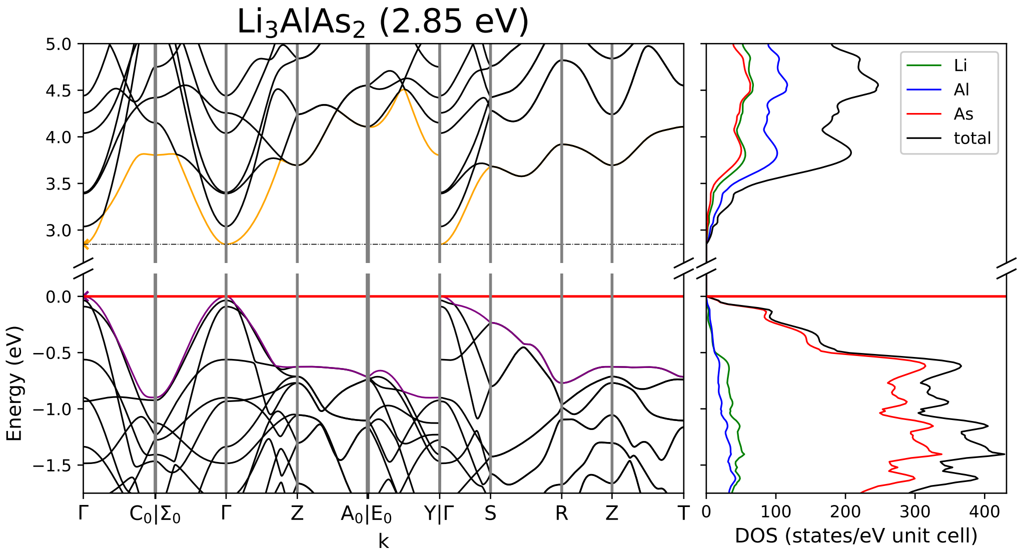

Figure S9. Band structure and DOS of Li_3AlAs_2.

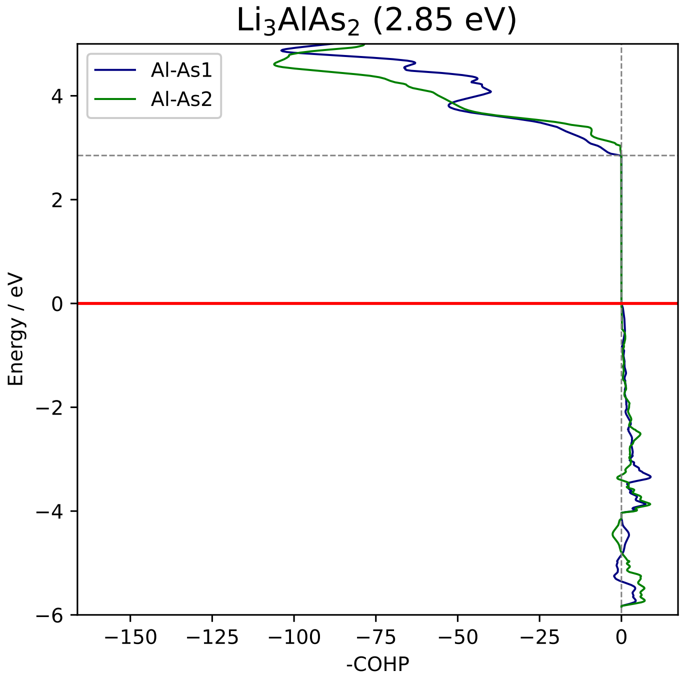

Figure S10. COHP of Al-As interactions in Li_3AlAs_2.

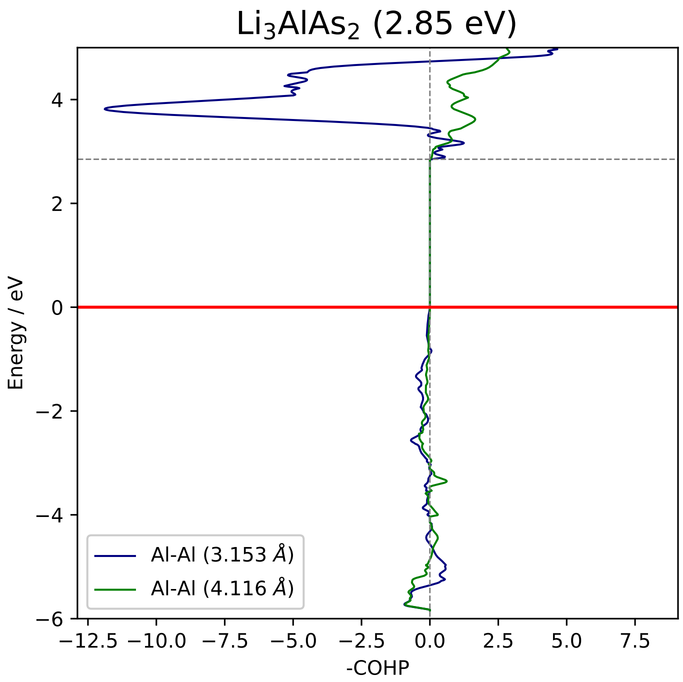

Figure S11. COHP of Al-Al interactions in Li_3AlAs_2.

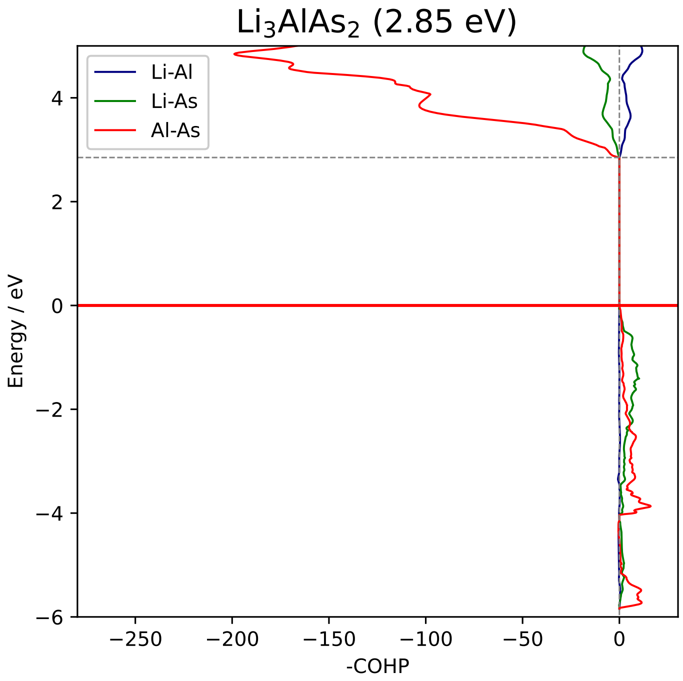

Figure S12. COHP of Li-Al, Li-As and Al-As interactions in Li_3AlAs_2.

Table S8. Overlap population and interatomic distances of Li_3AlAs_2.

Atom A	Atom B	r_{AB} / Å	overlap
As1	Al1	2.491	0.277
	Li1	2.64	0.07
	Li2	2.643	0.072
	Li2	2.644	0.072
	As1	3.857	−0.043
	As2	4.079	−0.026
	As2	4.148	−0.028
	As2	4.378	−0.004
	As1	4.379	−0.005
	As2	4.396	−0.005
As2	Al1	2.507	0.282
	Li1	2.62	0.061
	Li2	2.664	0.061
	Li2	2.676	0.083
	As2	4.15	−0.029
	As2	4.396	−0.005

Atom A	Atom B	r_{AB} / Å	overlap
Al1	Li1	3.007	0.013
	Li1	3.012	0.013
	Li2	3.138	0.01
	Al1	3.153	−0.014
	Al1	4.116	−0.006
Li1	Li1	2.765	0.01
	Li2	2.939	0.006
Li2	Li2	2.949	0.007
	Li2	3.012	0.005
	Li2	3.033	0.005

Table S9. Partial charges for each atom position in Li_3AlAs_2.

Atom	Z	charge	part charge
As1	33	34.126	−1.126
As2		34.086	−1.086
Al1	13	12.492	0.508

Atom	Z	charge	partialcharge
Li1	3	2.428	0.572
Li2		2.434	0.566

2.4 Li_3GaAs_2[2]

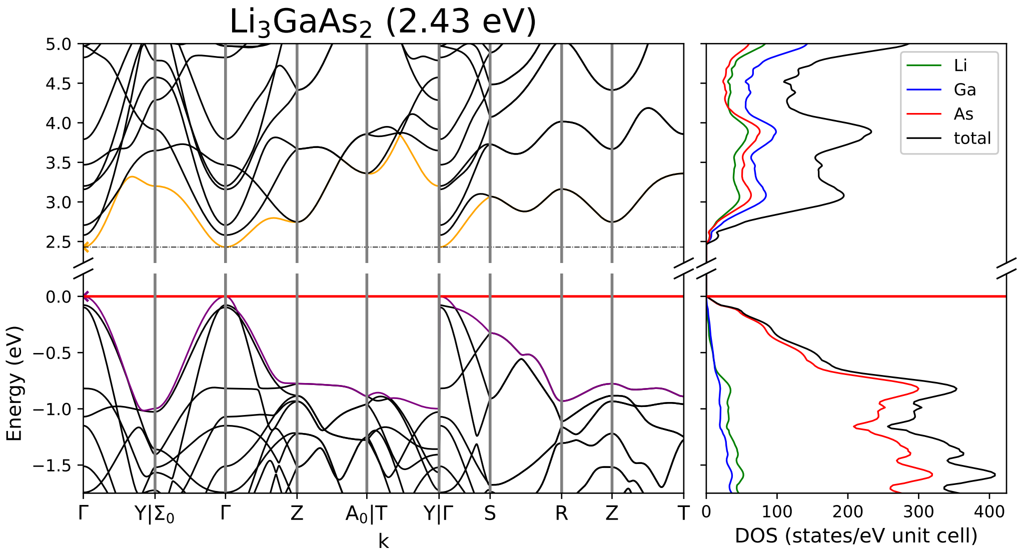

Figure S13. Band structure and DOS of Li_3GaAs_2.

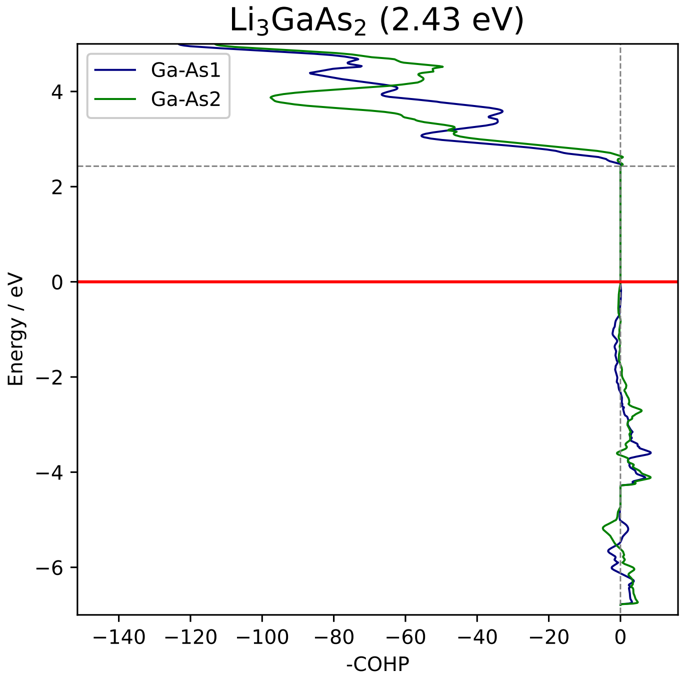

Figure S14. COHP of Ga-As interactions in Li_3GaAs_2.

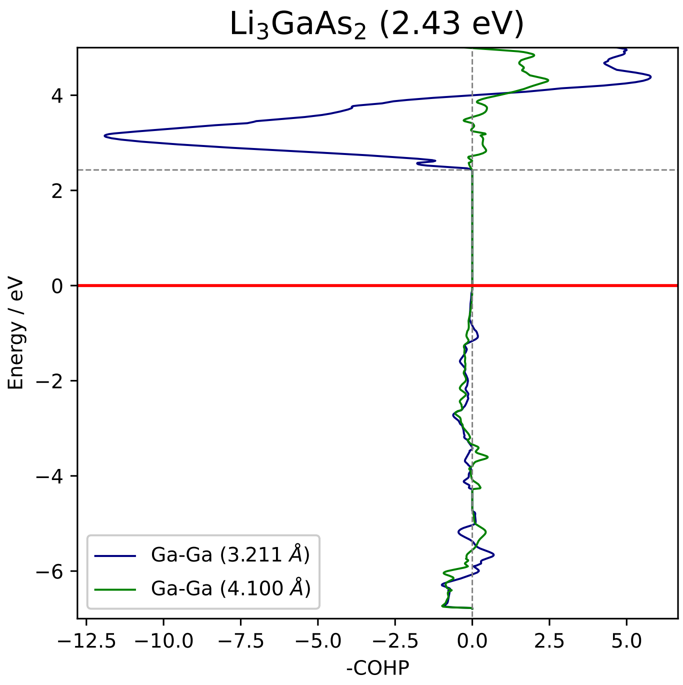

Figure S15. COHP of Ga-Ga interactions in Li_3GaAs_2.

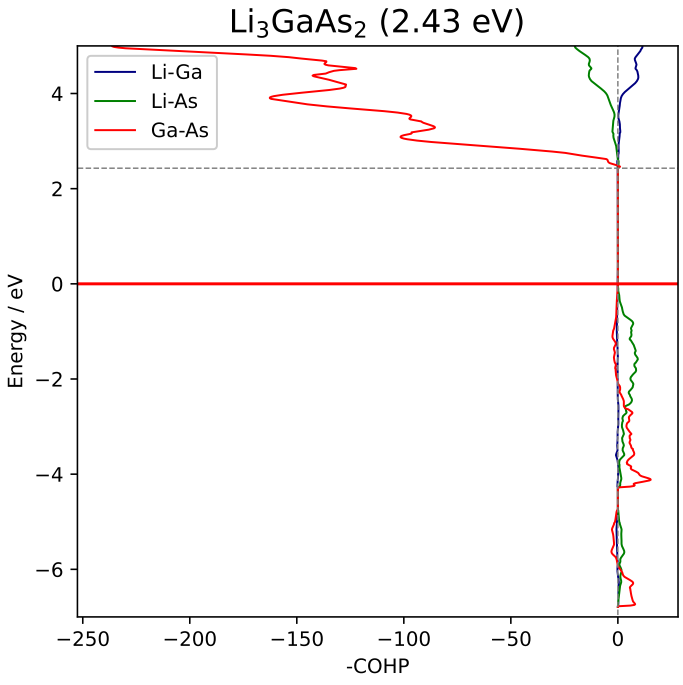

Figure S16. COHP of Li-Ga, Li-As and Ga-As interactions in Li_3GaAs_2.

Table S10. Overlap population and interatomic distances of Li_3GaAs_2.

Atom A	Atom B	r_{AB} / Å	overlap
Li1	As2	2.637	0.06
	As1	2.644	0.071
	Li1	2.796	0.01
	Li2	2.943	0.007
	Ga1	2.994	0.012
	Ga1	3.014	0.012
Li2	As1	2.637	0.074
	As1	2.654	0.071
	As2	2.668	0.086
	As2	2.675	0.059
	Li2	2.927	0.008
	Li2	3.014	0.006
	Li2	3.084	0.005
	Ga1	3.145	0.01

Atom A	Atom B	r_{AB} / Å	overlap
Ga1	As1	2.508	0.236
	As2	2.517	0.245
	Ga1	3.211	−0.033
	Ga1	4.1	−0.019
	As1	4.932	0.0
	As2	4.942	0.001
As1	As1	3.853	−0.032
	As2	4.095	−0.025
	As2	4.175	−0.029
	As2	4.379	−0.005
	As1	4.397	−0.006
	As2	4.405	−0.005
	As1	4.487	−0.008
As2	As2	4.192	−0.029
	As2	4.362	−0.005

Table S11. Partial charges for each atom position in Li_3GaAs_2.

Atom	Z	charge	part charge
Li1	3	2.425	0.575
Li2		2.44	0.56
Ga1	31	30.712	0.288

Atom	Z	charge	partialcharge
As1	33	34.012	−1.012
As2		33.97	−0.97

3 Structure type G

3.1 Li_3InP_2[3]

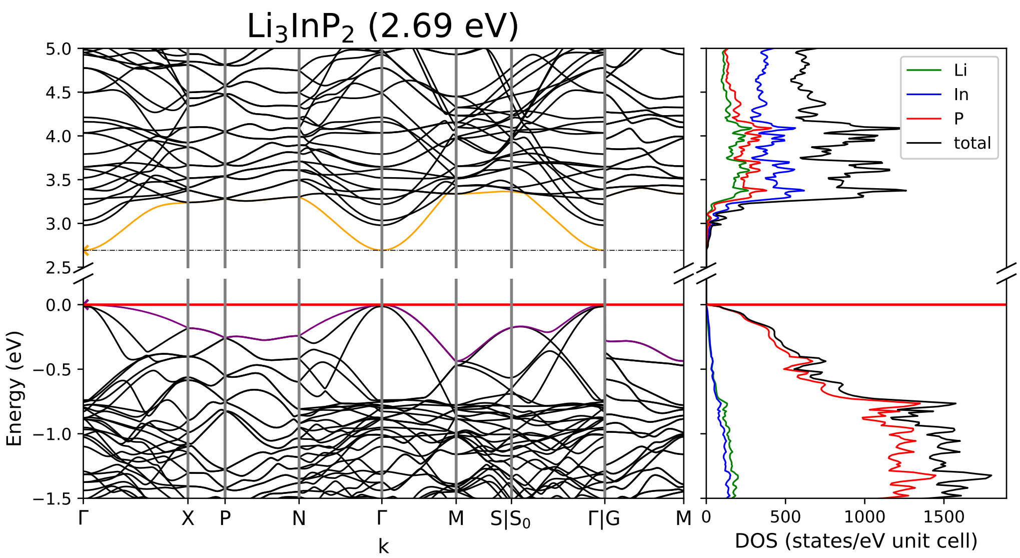

Figure S17. Band structure and DOS of Li_3InP_2.

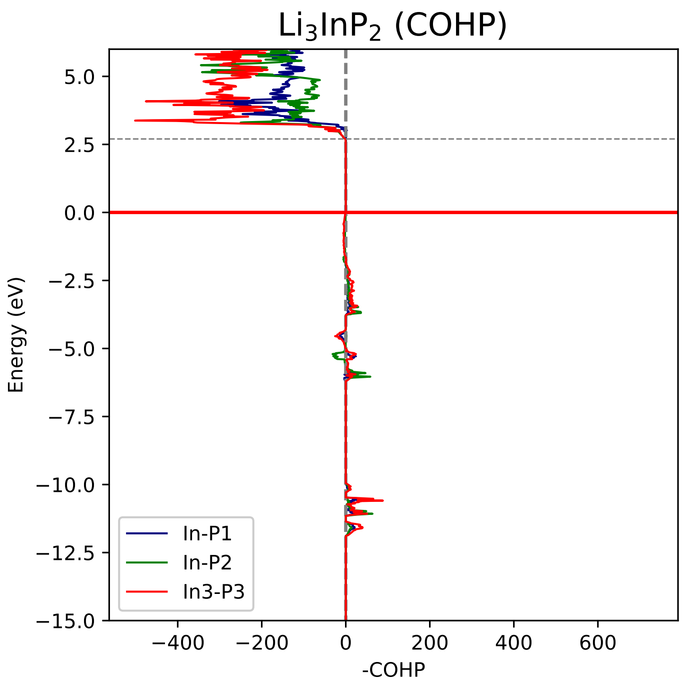

Figure S18. COHP of In-P interactions in Li_3InP_2.

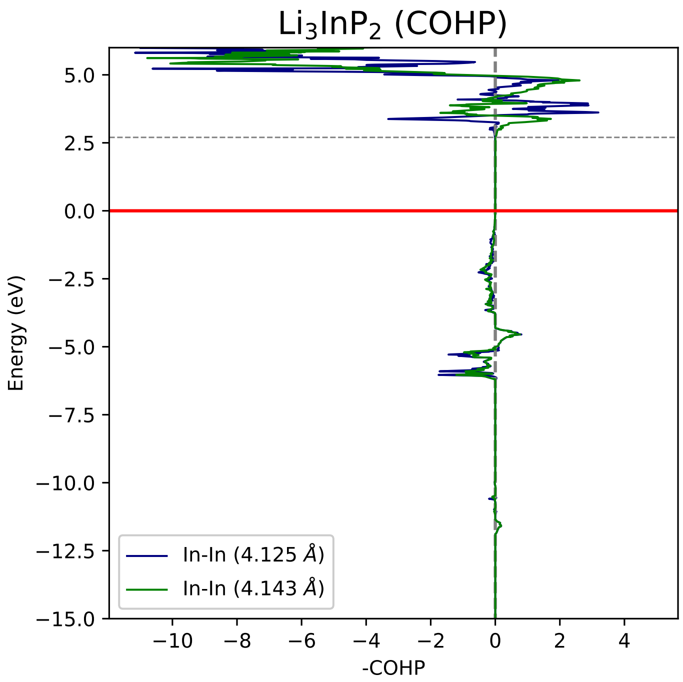

Figure S19. COHP of In-In interactions in Li_3InP_2.

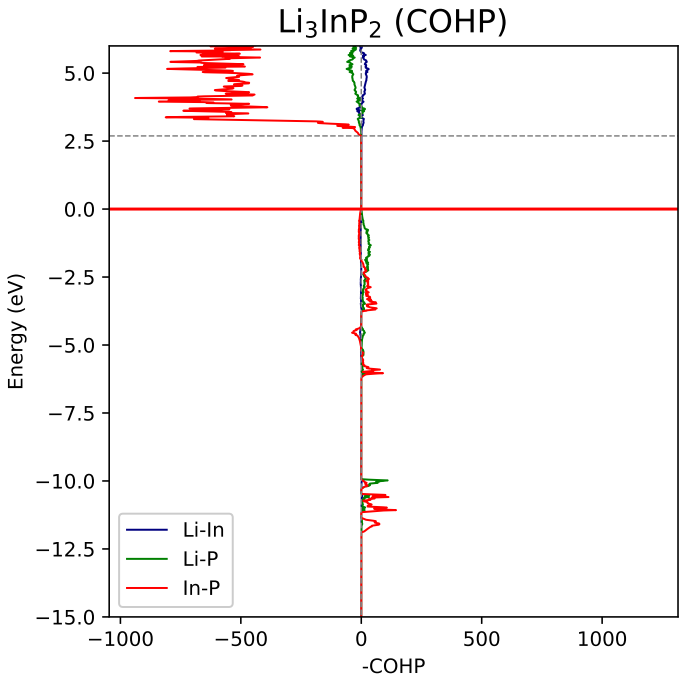

Figure S20. COHP of Li-In, Li-P and In-P interactions in Li_3InP_2.

Table S12. Overlap population and interatomic distances of Li_3InP_2.

Atom A	Atom B	r_{AB} / Å	overlap
Li1	P3	2.532	0.085
	P1	2.577	0.083
	P3	2.634	0.057
	P2	2.674	0.056
	Li3	2.903	0.007
	Li3	2.927	0.006
	Li2	2.967	0.005
	Li2	2.985	0.005
	Li1	3.063	0.004
	In1	3.151	0.01
Li2	P3	2.551	0.086
	P1	2.591	0.063
	P2	2.605	0.062
	P3	2.682	0.055
	Li3	2.857	0.007
	Li2	2.885	0.006
	In1	3.088	0.011
	In1	3.105	0.011
Li3	P2	2.535	0.086
	P3	2.635	0.058
	P3	2.658	0.056
	P1	2.682	0.054
	In1	3.048	0.012
	In1	3.061	0.012
	In1	3.064	0.012

Atom A	Atom B	r_{AB} / Å	overlap
In1	P1	2.574	0.225
	P3	2.577	0.223
	P2	2.594	0.231
	P3	2.595	0.232
	In1	4.125	−0.03
	In1	4.143	−0.031
P1	P2	4.16	−0.02
	P3	4.242	−0.004
	P3	4.246	−0.004
	P3	4.252	−0.022
	P3	4.257	−0.022
	P2	4.357	−0.007
P2	P3	4.197	−0.014
	P3	4.205	−0.019
	P3	4.297	−0.007
	P3	4.302	−0.01
P3	P3	4.16	−0.004
	P3	4.251	−0.023

Table S13. Partial charges for each atom position in Li_3InP_2.

Atom	Z	charge	part charge
Li1	3	2.426	0.574
Li2		2.418	0.582
Li3		2.418	0.582
In1	21	20.742	0.258

Atom	Z	charge	partialcharge
P1	15	16.004	−1.004
P2		15.988	−0.988
P3		16.0	−1.0

3.2 Li_3InAs_2[2]

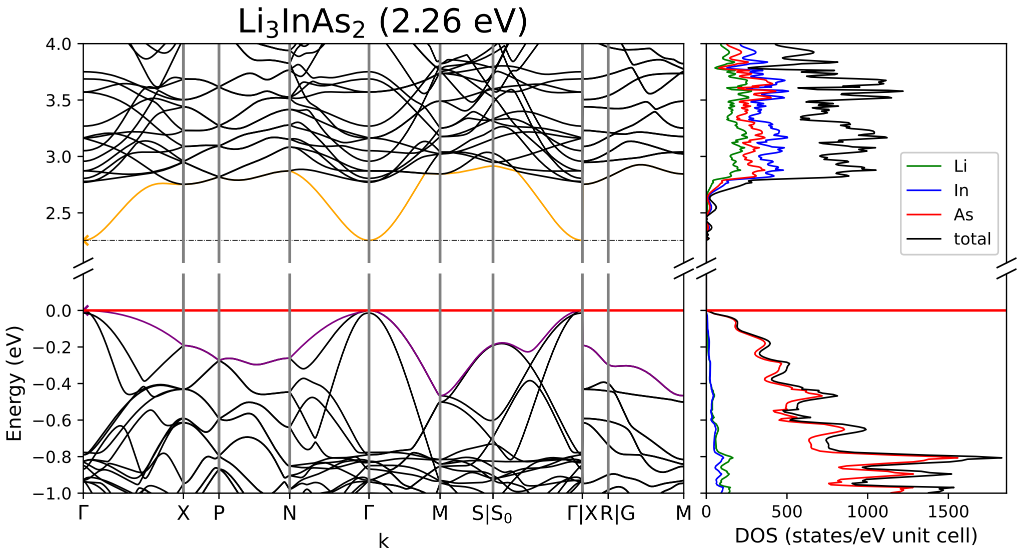

Figure S21. Band structure and DOS of Li_3InAs_2.

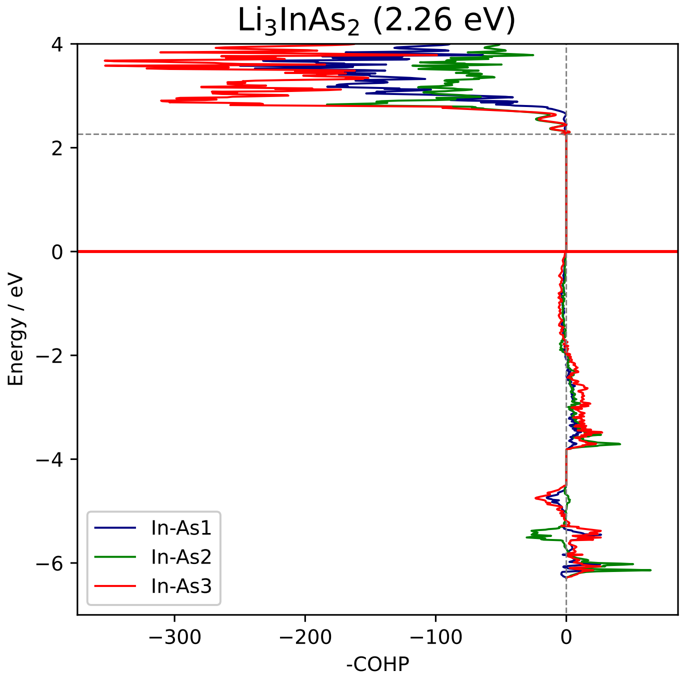

Figure S22. COHP of In-As interactions in Li_3InAs_2.

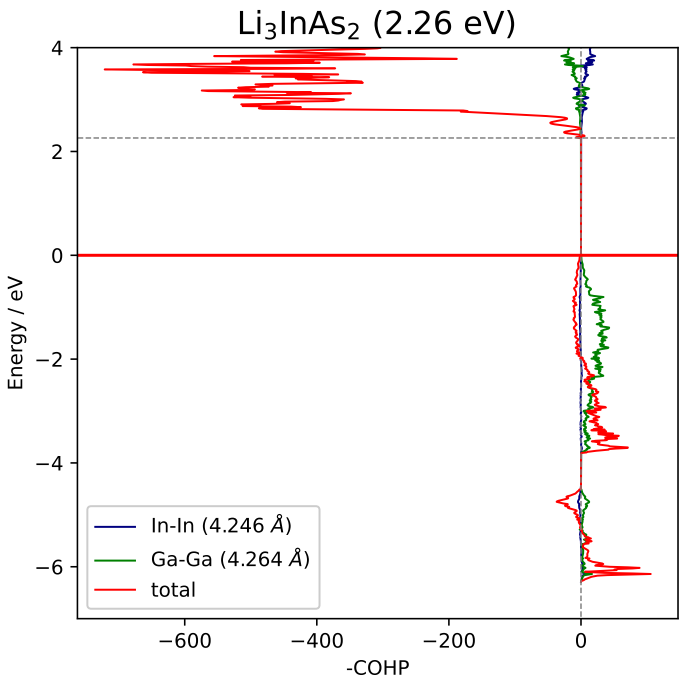

Figure S23. COHP of In-In interactions in Li_3InAs_2.

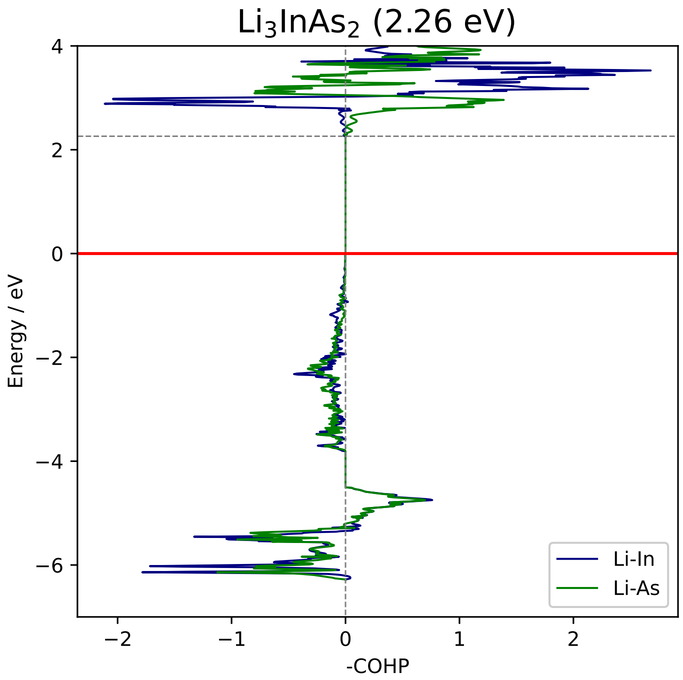

Figure S24. COHP of Li-In, Li-P and In-As interactions in Li_3InAs_2.

Table S14. Partial charges for each atom position in Li_3InAs_2.

Atom	Z	charge	part charge
Li1	3	2.42	0.58
Li2		2.423	0.577
Li3		2.43	0.57
In1	21	20.633	0.367

Atom	Z	charge	partialcharge
As1	33	34.055	−1.055
As2		34.038	−1.038
As3		34.048	−1.048

Table S15. Overlap population and interatomic distances of Li_3InAs_2.

Atom A	Atom B	r_{AB} / Å	overlap
Li1	As3	2.632	0.089
	As2	2.667	0.068
	As1	2.677	0.061
	As3	2.76	0.054
	Li2	2.943	0.007
	Li1	2.997	0.006
	Li3	3.082	0.005
	Li3	3.104	0.004
	In1	3.176	0.01
	In1	3.182	0.011
Li2	As2	2.634	0.085
	As3	2.717	0.06
	As3	2.733	0.059
	As1	2.76	0.057
	Li3	2.991	0.006
	Li3	3.025	0.006
	In1	3.12	0.012
	In1	3.134	0.012
	In1	3.138	0.011

Atom A	Atom B	r_{AB} / Å	overlap
Li3	As3	2.61	0.088
	As1	2.65	0.086
	As3	2.709	0.057
	As2	2.726	0.057
	Li3	3.192	0.004
	In1	3.239	0.009
In1	As1	2.66	0.218
	As3	2.664	0.216
	As3	2.684	0.226
	As2	2.687	0.228
	In1	4.246	−0.019
	In1	4.264	−0.02
As1	As2	4.298	−0.019
	As3	4.353	−0.004
	As3	4.354	−0.004
	As3	4.406	−0.02
	As3	4.407	−0.021
	As2	4.478	−0.006
As2	As3	4.325	−0.012
	As3	4.347	−0.017
	As3	4.413	−0.007
	As3	4.437	−0.009
As3	As3	4.257	−0.004
	As3	4.406	−0.021
	As3	4.483	−0.003

4 Structure type E

4.1 Na_3AlP_2[4]

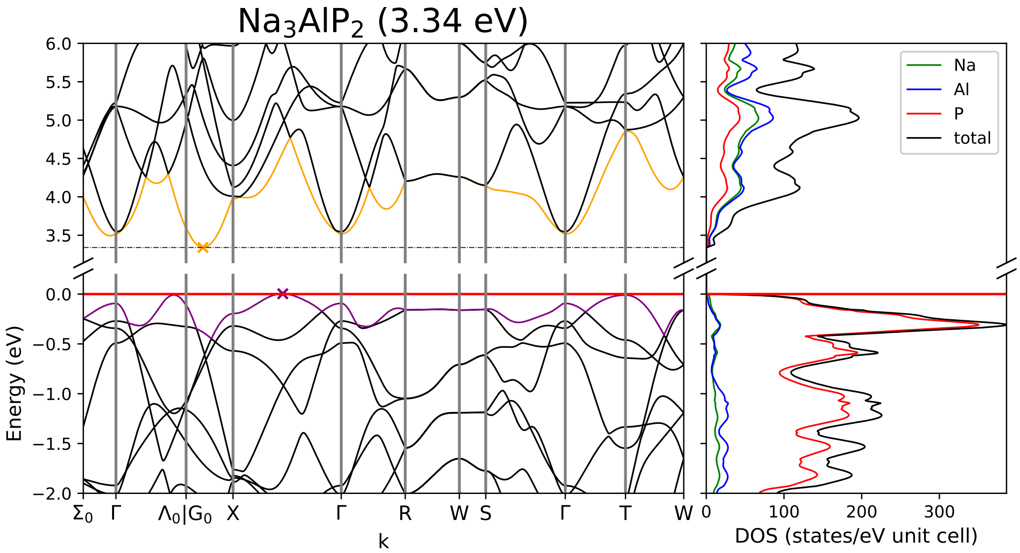

Figure S25. Band structure and DOS of Na_3AlP_2.

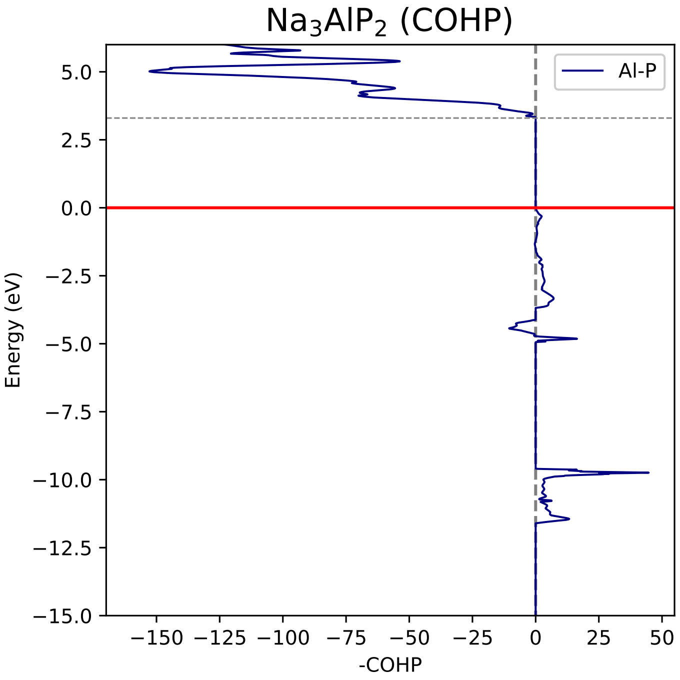

Figure S26. COHP of Al-P interactions in Na_3AlP_2.

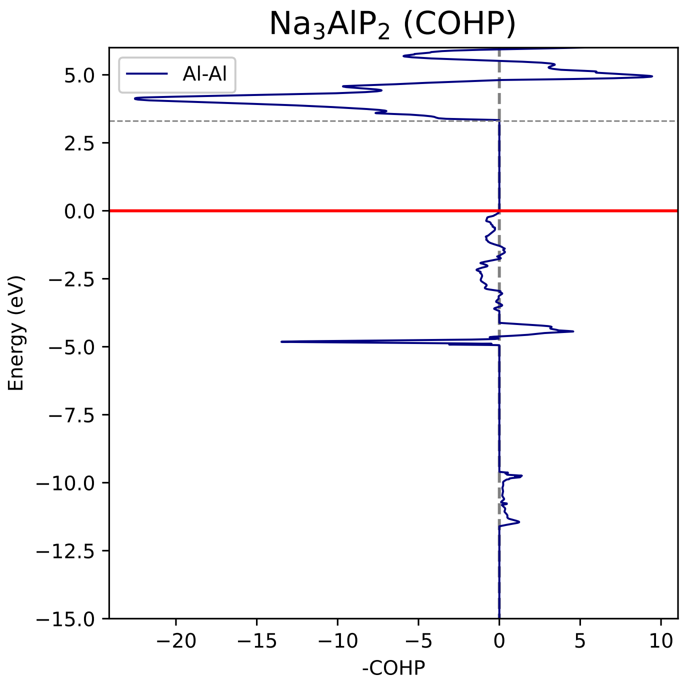

Figure S27. COHP of Al-Al interactions in Na_3AlP_2.

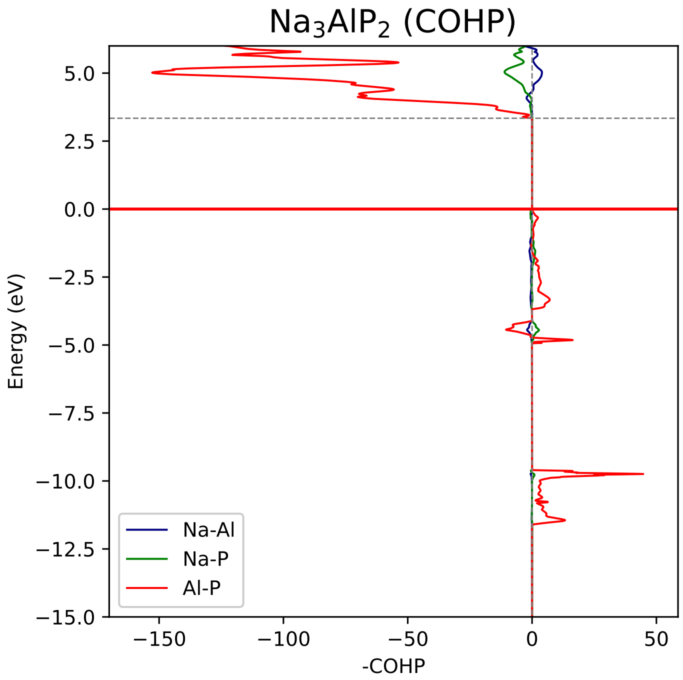

Figure S28. COHP of Na-Al, Na-P and Al-P interactions in Na_3AlP_2.

Table S16. Overlap population and interatomic distances of Na_3AlP_2.

Atom A	Atom B	r_{AB} / Å	overlap
Na1	P1	2.83	0.059
	P1	2.877	0.027
	P1	3.196	0.025
	Al1	3.198	0.003
	Na2	3.448	0.003
	Na1	3.481	0.003
	Na1	3.646	0.002
	P1	3.859	0.003
	P1	3.958	0.012
Na2	P1	2.812	0.04
	Na2	3.004	0.006
	Al1	3.234	0.005

Atom A	Atom B	r_{AB} / Å	overlap
Al1	P1	2.423	0.291
	Al1	3.004	0.008
	P1	4.891	−0.001
P1	P1	3.801	−0.072
	P1	3.975	−0.037
	P1	4.086	−0.034
	P1	4.755	−0.005
	P1	4.895	0.0
	P1	4.932	−0.001

Table S17. Partial charges for each atom position in Na_3AlP_2.

Atom	Z	charge	part charge
Na1	11	10.244	0.756
Na2		10.282	0.718
Al1	13	12.859	0.141

Atom	Z	charge	partialcharge
P1	15	16.185	−1.185

4.2 Na_3GaP_2[5]

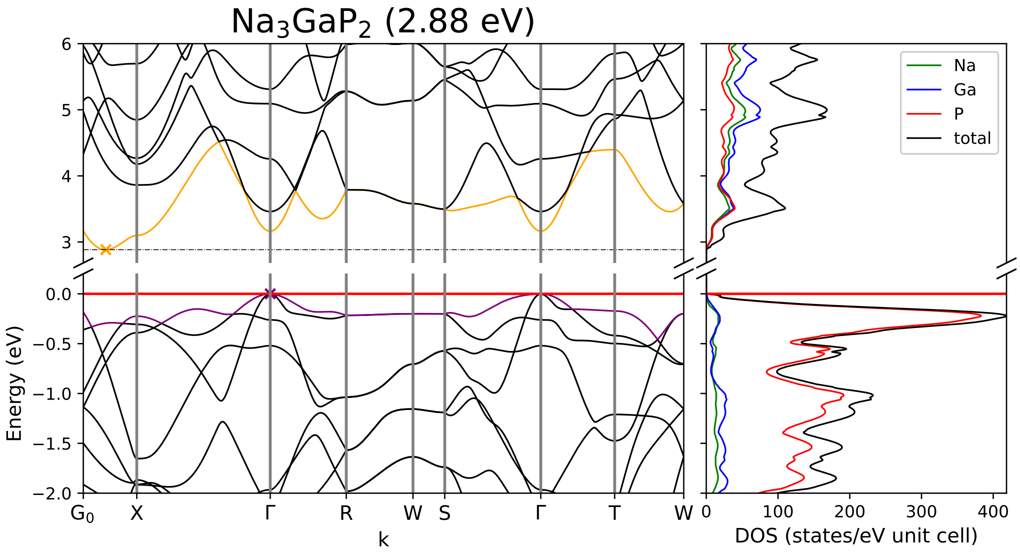

Figure S29. Band structure and DOS of Na_3GaP_2.

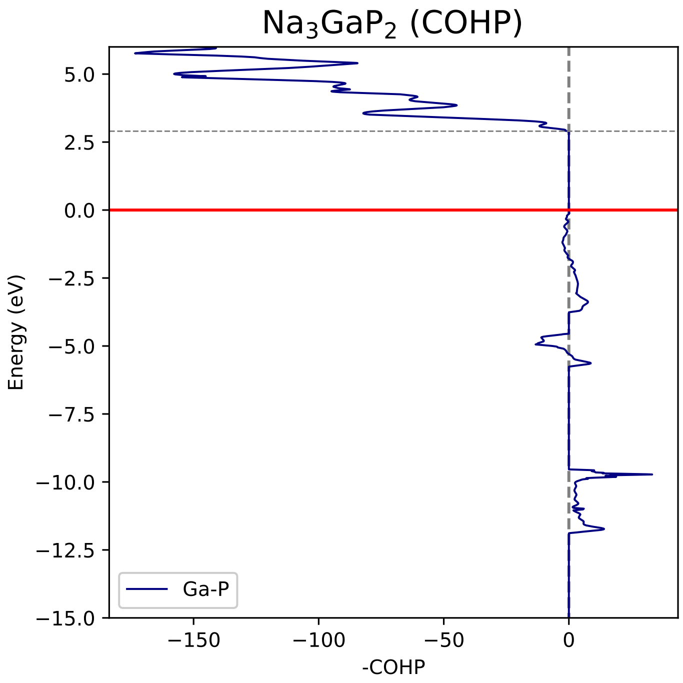

Figure S30. COHP of Ga-P interactions in Na_3GaP_2.

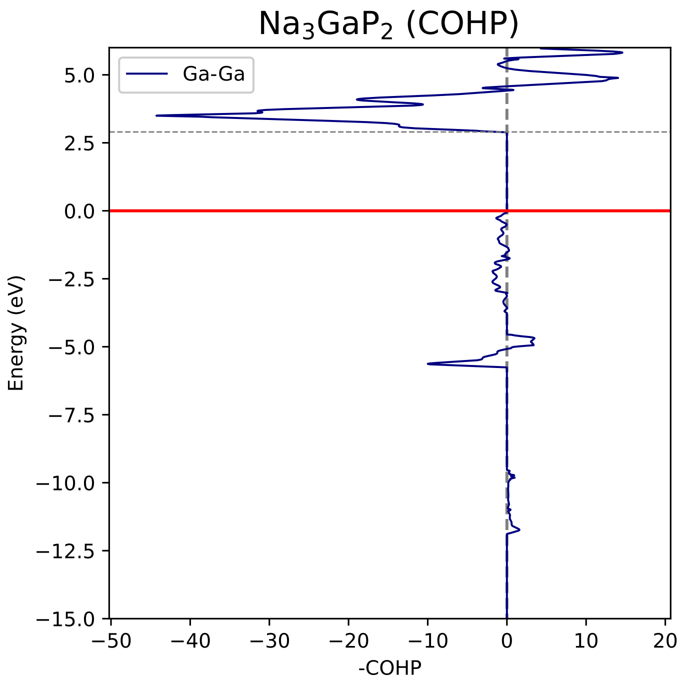

Figure S31. COHP of Ga-Ga interactions in Na_3GaP_2.

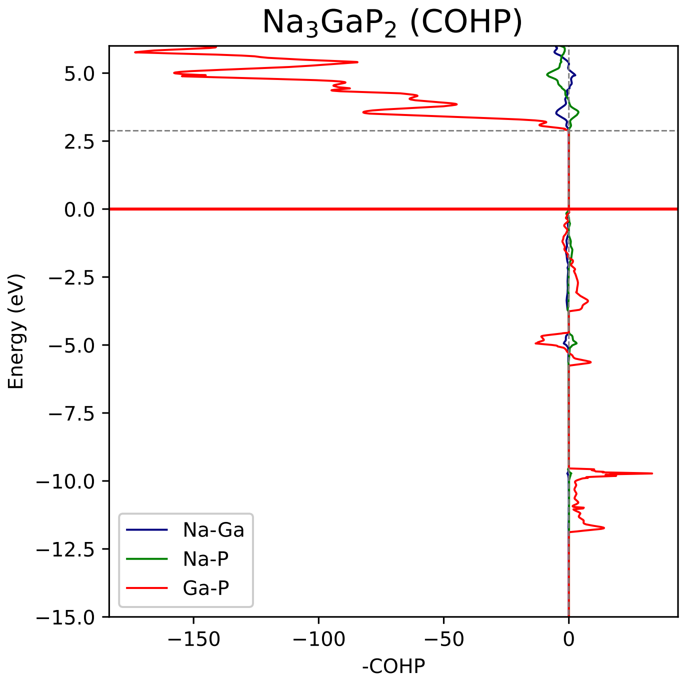

Figure S32. COHP of Na-Ga, Na-P and Ga-P interactions in Na_3GaP_2.

Table S18. Overlap population and interatomic distances of Na_3GaP_2.

Atom A	Atom B	r_{AB} / Å	overlap
Na1	P1	2.804	0.04
	Na1	3.061	0.006
	Ga1	3.17	0.002
	Na2	3.392	0.003
Na2	P1	2.834	0.061
	P1	2.88	0.028
	Ga1	3.21	0.001
	P1	3.237	0.021
	Na2	3.545	0.003
	Na2	3.619	0.002
	P1	3.802	0.017
	P1	3.861	0.003

Atom A	Atom B	r_{AB} / Å	overlap
Ga1	P1	2.446	0.267
	Ga1	3.061	−0.038
	P1	4.973	−0.002
P1	P1	3.816	−0.06
	P1	4.001	−0.036
	P1	4.159	−0.032
	P1	4.7	−0.006
	P1	4.851	0.0
	P1	4.944	0.0

Table S19. Partial charges for each atom position in Na_3GaP_2.

Atom	Z	charge	part charge
Na1	11	10.276	0.724
Na2		10.237	0.763
Ga1	31	31.13	−0.13

Atom	Z	charge	partialcharge
P1	15	16.06	−1.06

4.3 K_3InP_2[4]

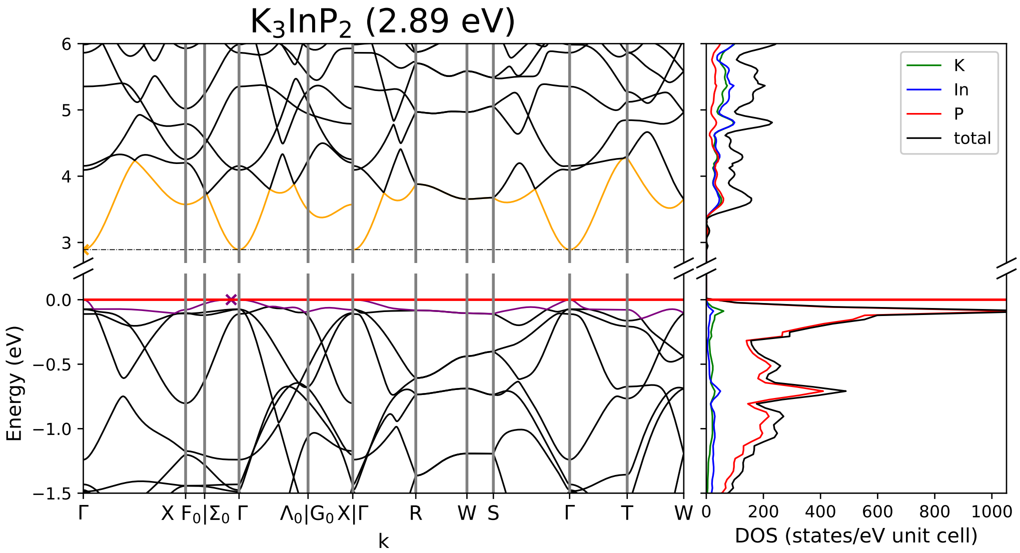

Figure S33. Band structure and DOS of K_3InP_2.

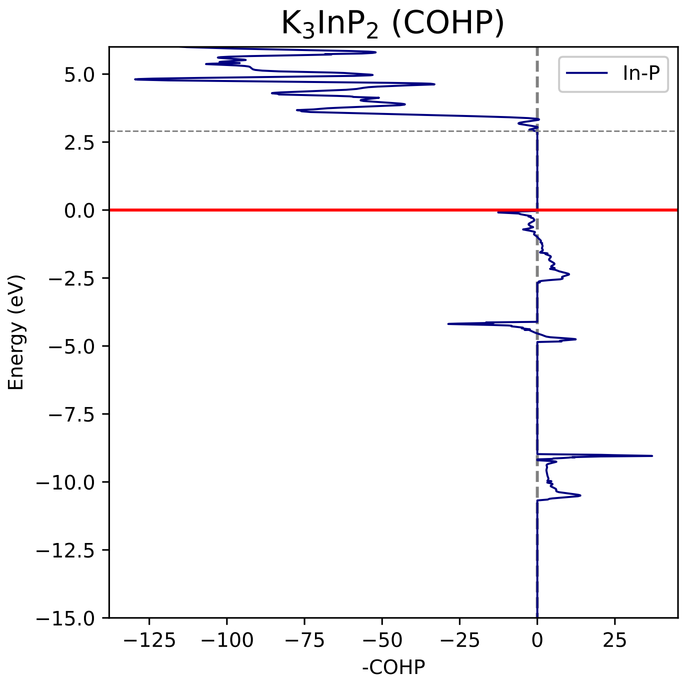

Figure S34. COHP of In-P interactions in K_3InP_2.

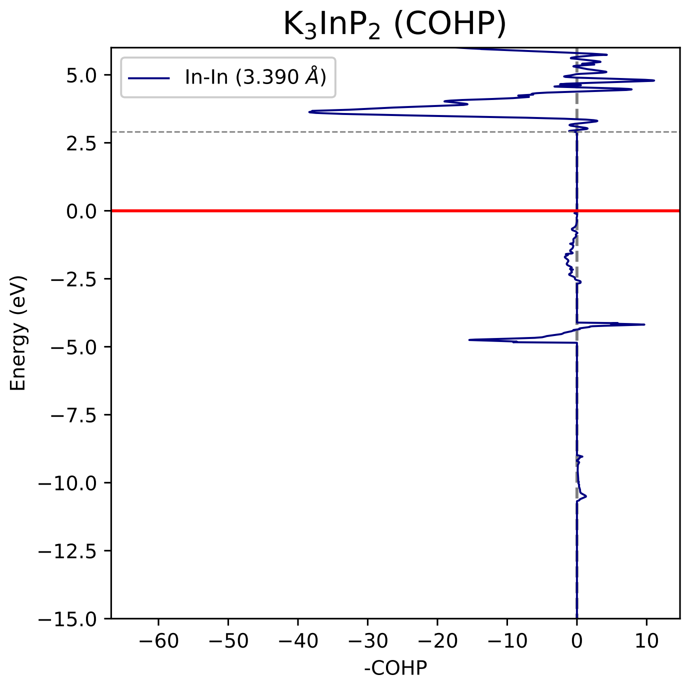

Figure S35. COHP of In-In interactions in K_3InP_2.

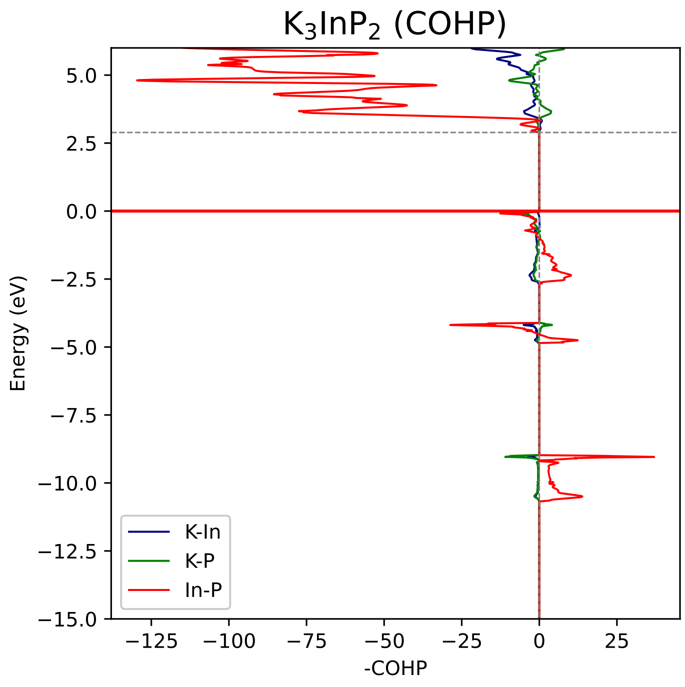

Figure S36. COHP of K-In, K-P and In-P interactions in K_3InP_2.

Table S20. Partial charges for each atom position in K_3InP_2.

Atom	Z	charge	part charge
K1	19	18.226	0.774
K2		18.26	0.74
In1	21	20.914	0.086

Atom	Z	charge	partialcharge
P1	15	16.187	−1.187

Table S21. Overlap population and interatomic distances of K_3InP_2.

Atom A	Atom B	r_{AB} / Å	overlap
K1	P1	3.251	0.003
	P1	3.263	0.038
	In1	3.517	−0.01
	P1	3.605	0.014
	K2	3.825	0.001
	K1	3.963	0.002
K2	P1	3.202	0.016
	K2	3.39	−0.002
	In1	3.749	−0.009

Atom A	Atom B	r_{AB} / Å	overlap
In1	P1	2.646	0.261
	In1	3.39	−0.048
P1	P1	4.063	−0.052
	P1	4.438	−0.022
	P1	4.45	−0.02

4.4 Na_3AlAs_2[6]

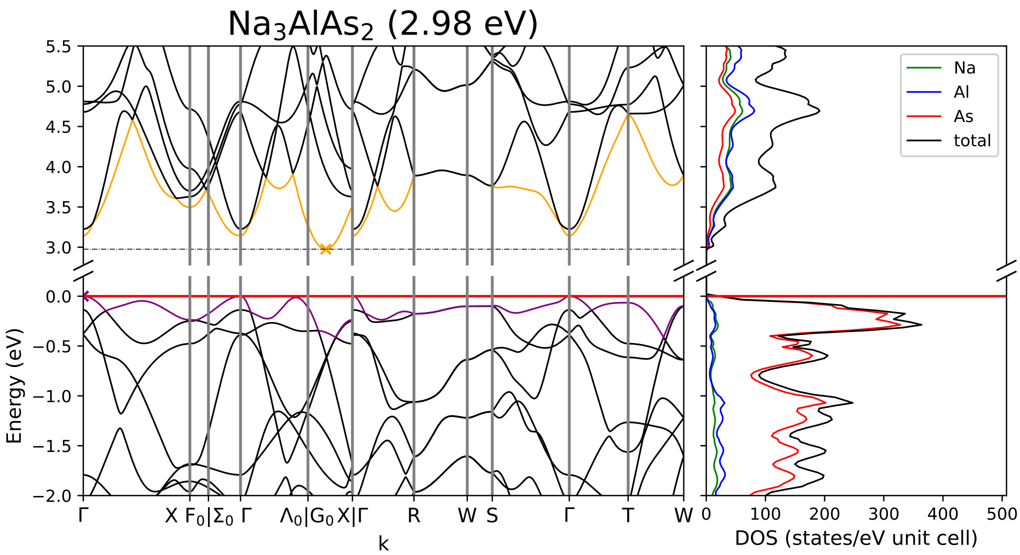

Figure S37. Band structure and DOS of Na_3AlAs_2.

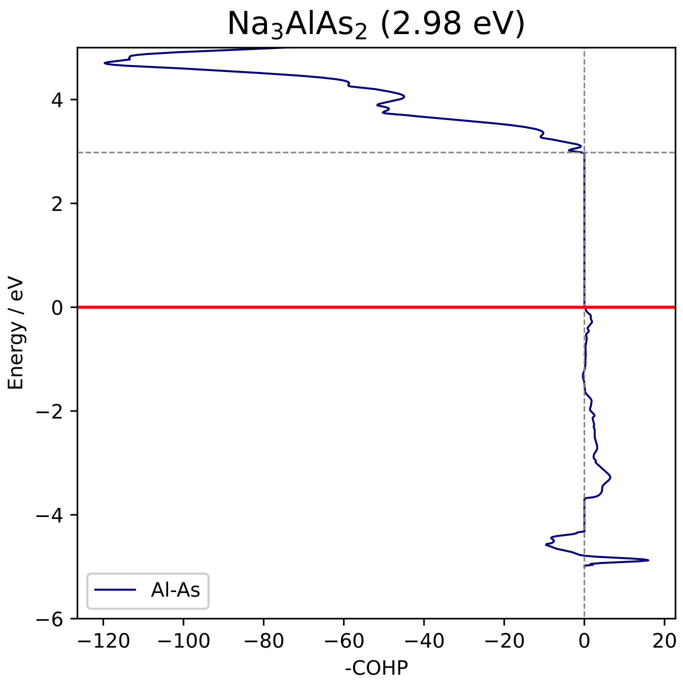

Figure S38. COHP of Al-As interactions in Na_3AlAs_2.

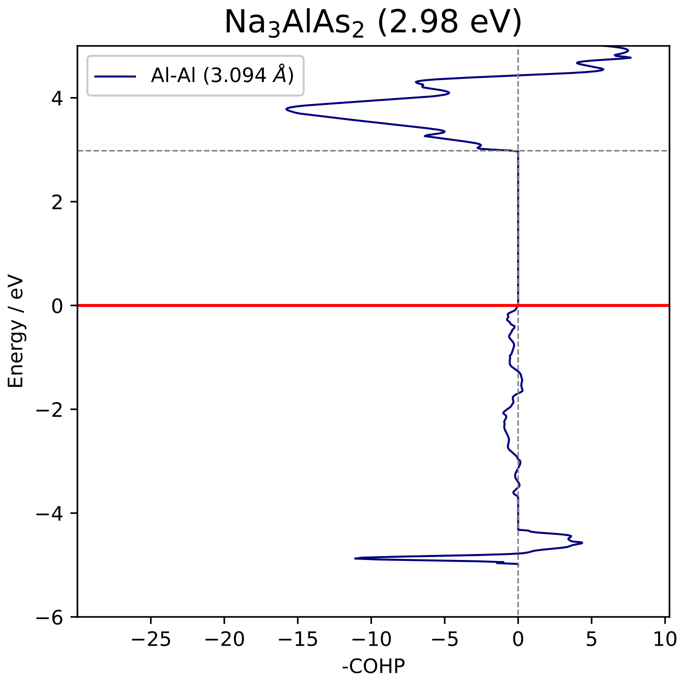

Figure S39. COHP of Al-Al interactions in Na_3AlAs_2.

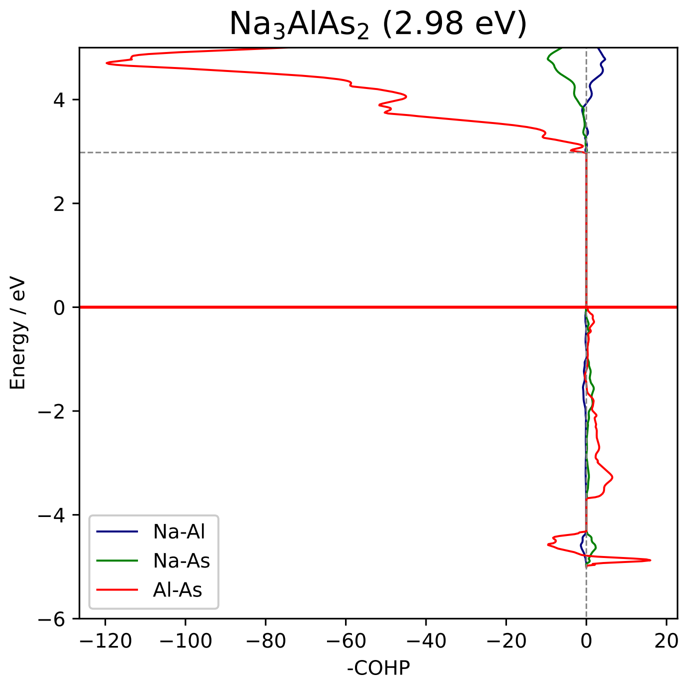

Figure S40. COHP of Na-Al, Na-As and Al-As interactions in Na_3AlAs_2.

Table S22. Overlap population and interatomic distances of Na_3AlAs_2.

Atom A	Atom B	r_{AB} / Å	overlap
Na1	As1	2.912	0.062
	As1	2.991	0.032
	As1	3.261	0.026
	Al1	3.298	0.006
	Na2	3.416	0.004
	Na1	3.622	0.002

Atom A	Atom B	r_{AB} / Å	overlap
Na2	As1	2.87	0.045
	Na2	3.094	0.006
	Al1	3.229	0.008
Al1	As1	2.52	0.283
	Al1	3.094	0.012

Table S23. Partial charges for each atom position in Na_3AlAs_2.

Atom	Z	charge	part charge
Na1	11	10.27	0.73
Na2		10.319	0.681
Al1	13	12.743	0.257

Atom	Z	charge	partialcharge
As1	33	34.199	−1.199

4.5 K_3InAs_2[7]

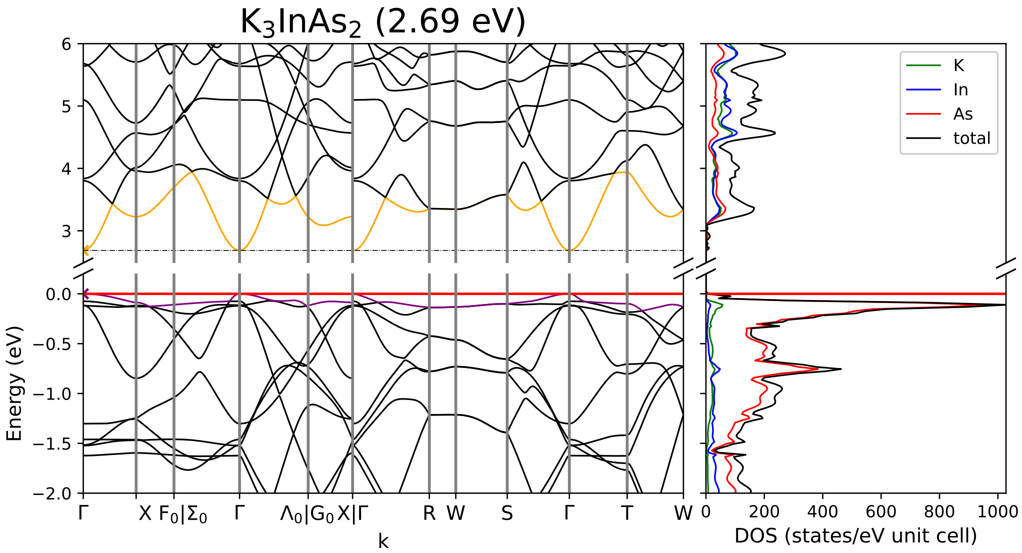

Figure S41. Band structure and DOS of K_3InAs_2.

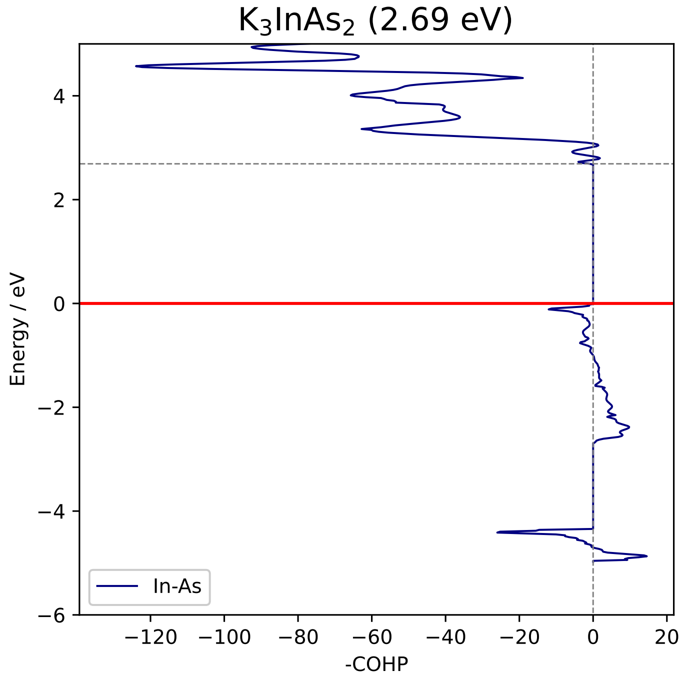

Figure S42. COHP of In-As interactions in K_3InAs_2.

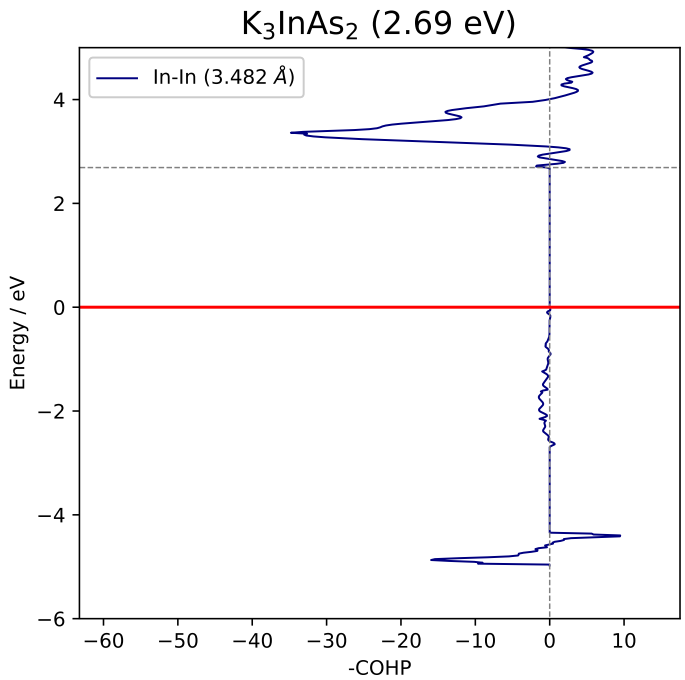

Figure S43. COHP of In-In interactions in K_3InAs_2.

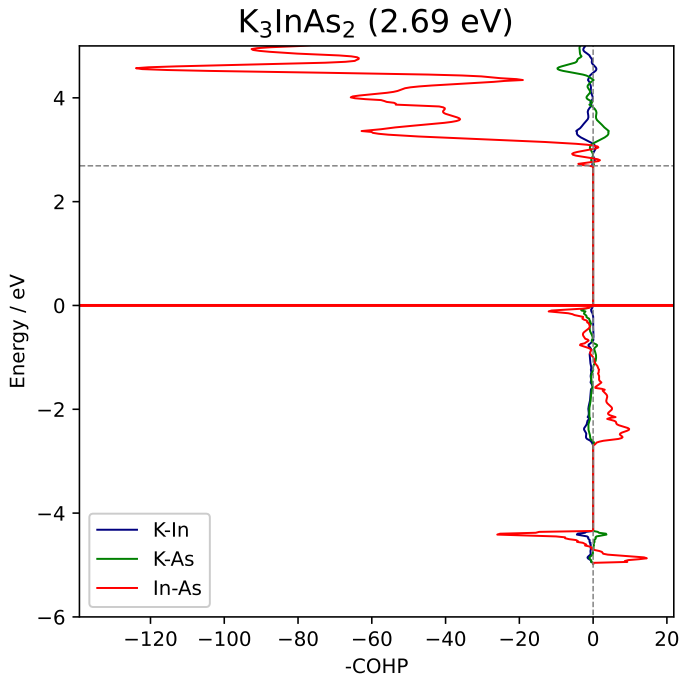

Figure S44. COHP of K-In, K-As and In-As interactions in K_3InAs_2.

Table S24. Overlap population and interatomic distances of K_3InAs_2.

Atom A	Atom B	r_{AB} / Å	overlap
K1	As1	3.333	0.01
	As1	3.339	0.043
	In1	3.572	−0.006
	As1	3.678	0.015
	K2	3.853	0.001

Atom A	Atom B	r_{AB} / Å	overlap
K2	As1	3.256	0.021
	K2	3.482	0.0
	In1	3.807	−0.007
In1	As1	2.728	0.254
	In1	3.482	−0.035

Table S25. Partial charges for each atom position in K_3InAs_2.

Atom	Z	charge	part charge
K1	19	18.243	0.757
K2		18.286	0.714

Atom	Z	charge	partialcharge
In1	21	20.831	0.169
As1	33	34.198	−1.198

5 Structure type H

5.1 Na_3InP_2[8]

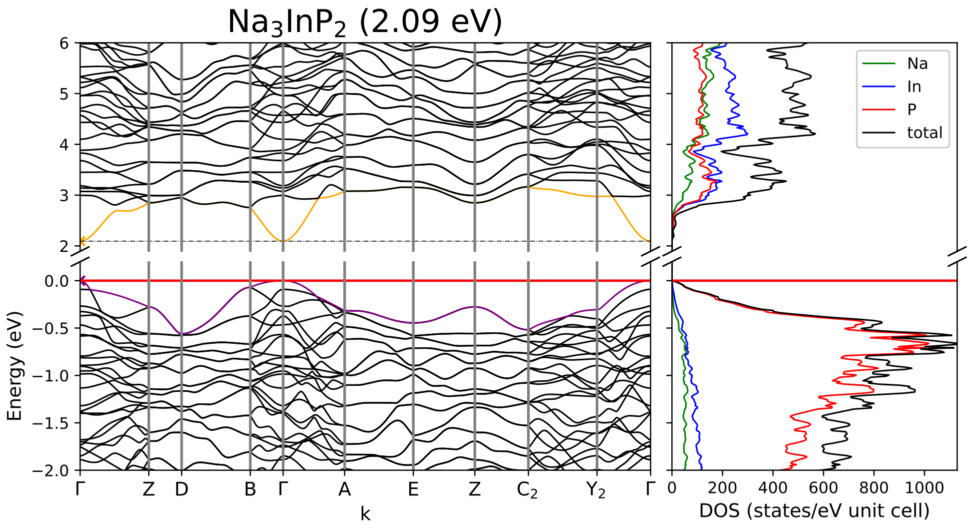

Figure S45. Band structure and DOS of Na_3InP_2.

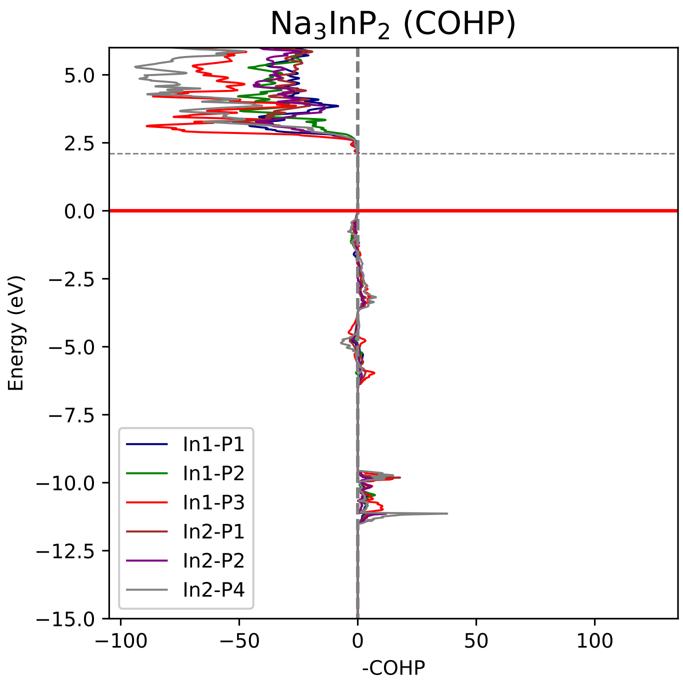

Figure S46. COHP of In-P interactions in Na_3InP_2.

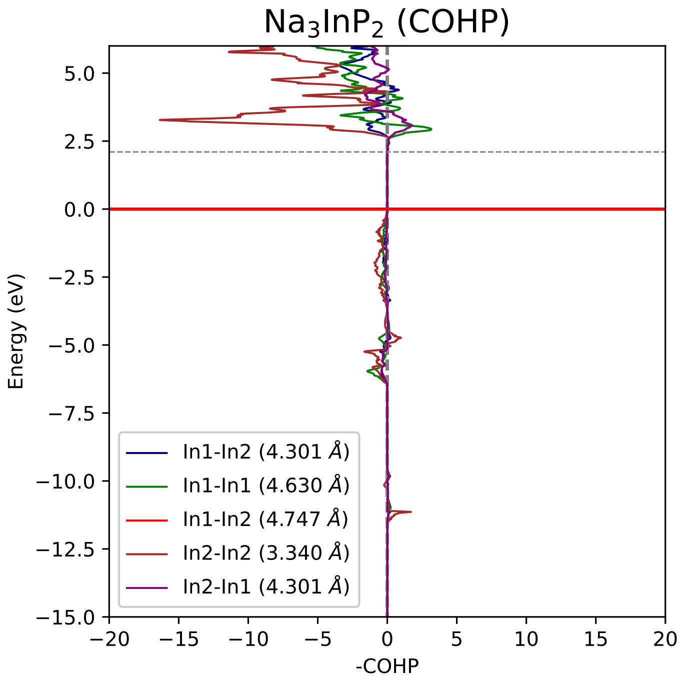

Figure S47. COHP of In-In interactions in Na_3InP_2.

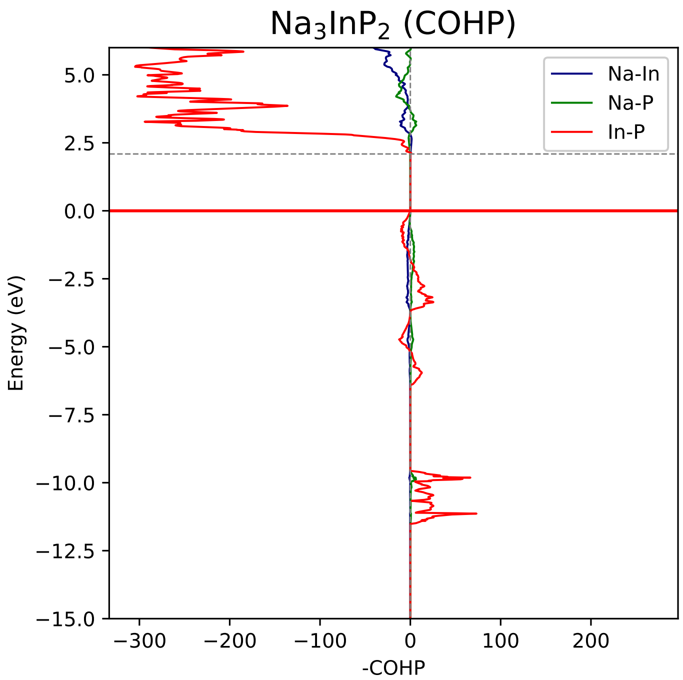

Figure S48. COHP of Na-In, Na-P and In-P interactions in Na_3InP_2.

Table S26. Partial charges for each atom position in Na_3InP_2.

Atom	Z	charge	part charge
Na1	11	10.266	0.734
Na2		10.232	0.768
Na3		10.242	0.758
Na4		10.305	0.695
Na5		10.227	0.773
Na6		10.231	0.769
In1	21	21.159	−0.159

Atom	Z	charge	partialcharge
In2		21.047	−0.047
P1	15	16.062	−1.062
P2		16.067	−1.067
P3		16.048	−1.048
P4		16.116	−1.116

Table S27. Overlap population and interatomic distances of Na_3InP_2.

Atom A	Atom B	r_{AB} / Å	overlap
Na1	P4	2.779	0.042
	P3	2.831	0.043
	P2	2.86	0.052
	P2	2.867	0.023
	Na3	3.222	0.006
	Na5	3.237	0.004
	In2	3.264	−0.001
	Na5	3.266	0.005
	Na3	3.282	0.003
	Na6	3.319	0.005
	In1	3.39	−0.002
	Na2	3.536	0.001
Na2	P1	2.884	0.048
	P3	2.993	0.021
	P1	3.077	0.033
	P3	3.176	0.027
	In1	3.181	0.003
	Na4	3.219	0.006
	In2	3.247	0.004
	Na4	3.378	0.004
	In1	3.404	0.004
	Na2	3.412	0.005
	P4	3.482	0.014
	Na3	3.528	0.002
	Na6	3.799	0.001
	P2	3.87	−0.001
	Na6	3.902	0.0
Na3	P4	3.028	0.028
	P3	3.032	0.052
	P1	3.086	0.017
	P2	3.103	0.031
	In2	3.26	0.003
	Na4	3.274	0.005
	P2	3.345	0.024
	In1	3.381	0.005
	In2	3.515	0.001
	Na6	3.534	0.002
	Na5	3.779	0.002
	Na5	3.971	0.0
Na4	P3	2.785	0.041
	P4	2.817	0.052
	P1	2.85	0.057
	P1	2.86	0.04
	Na6	3.091	0.005
	Na5	3.324	0.003
	Na6	3.344	0.003
	In1	3.368	−0.002
	Na4	3.37	0.003
Na5	P4	2.933	0.037
	P4	3.007	0.033
	P2	3.067	0.034
	P1	3.182	0.013
	P2	3.329	0.022
	In2	3.347	0.007
	In1	3.4	0.004
	In2	3.478	0.003
	Na5	3.547	0.0
	Na6	3.621	0.002
Na6	P2	2.843	0.027
	P3	3.081	0.033
	P4	3.199	0.036
	P3	3.204	0.016
	P1	3.242	0.026
	In1	3.315	0.003
	In1	3.413	0.004
	In2	3.445	0.004
	P1	3.896	0.004
In1	P2	2.6	0.256
	P3	2.604	0.274
	P1	2.649	0.249
	P3	2.675	0.235
	In2	4.301	−0.03
	In1	4.63	−0.027
	P3	4.665	0.005
	In2	4.747	−0.018
In2	P4	2.585	0.249
	P2	2.624	0.246
	P1	2.625	0.261
	P4	2.641	0.228
	In2	3.34	−0.075
	P4	4.657	0.005
P1	P4	4.211	−0.026
	P2	4.287	−0.019
	P3	4.323	−0.018
	P3	4.331	−0.018
	P2	4.408	−0.015
	P4	4.435	−0.005
	P4	4.475	−0.018
	P3	4.589	−0.003
	P1	4.61	−0.005
P2	P4	4.128	−0.023
	P3	4.291	−0.022
	P4	4.364	−0.023
	P3	4.405	−0.02
	P2	4.546	−0.003
	P4	4.813	−0.003
	P4	4.839	−0.002
P3	P3	4.125	−0.022
	P4	4.531	−0.002
	P4	4.726	−0.003
P4	P4	4.019	−0.046
	P4	4.766	−0.002

5.2 Na_3InAs_2[9]

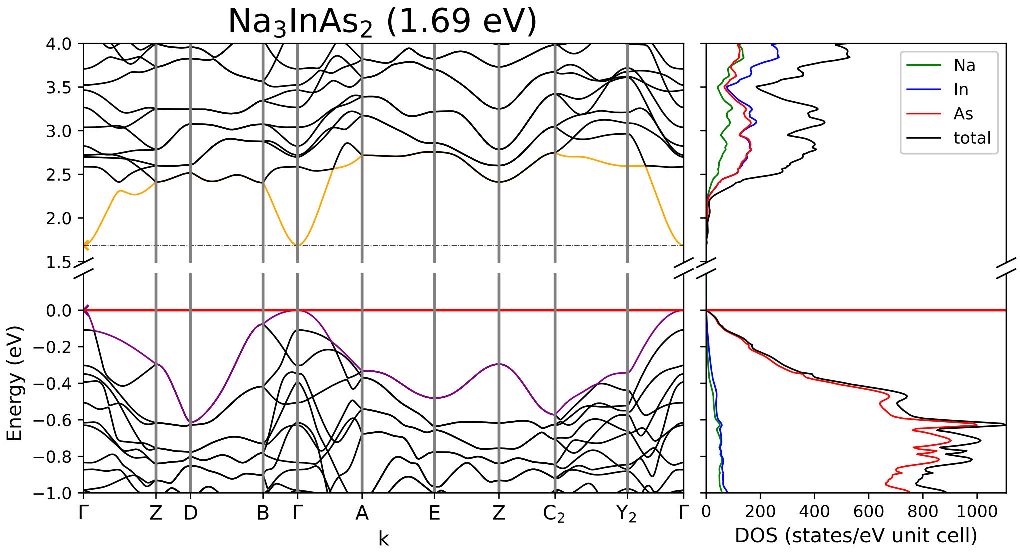

Figure S49. Band structure and DOS of Na_3InAs_2.

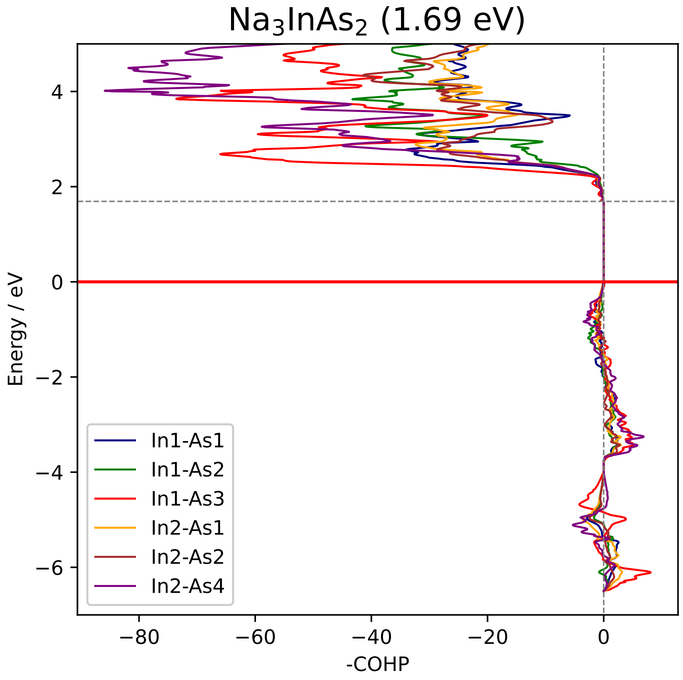

Figure S50. COHP of In-As interactions in Na_3InAs_2.

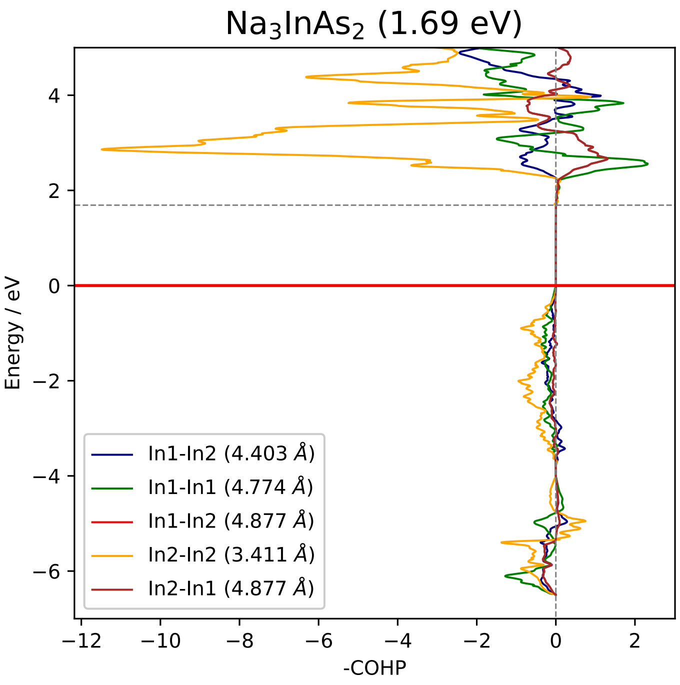

Figure S51. COHP of In-In interactions in Na_3InAs_2.

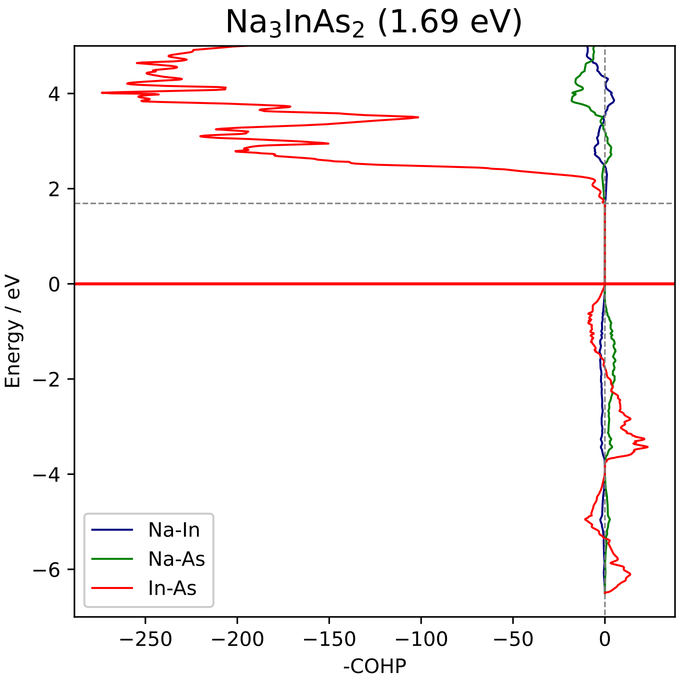

Figure S52. COHP of Na-In, Na-As and In-As interactions in Na_3InAs_2.

Table S28. Partial charges for each atom position in Na_3InAs_2.

Atom	Z	charge	part charge
Na1	11	10.293	0.707
Na2		10.26	0.74
Na3		10.269	0.731
Na4		10.332	0.668
Na5		10.251	0.749
Na6		10.257	0.743
In1	21	21.07	−0.07

Atom	Z	charge	partialcharge
In2		20.95	0.05
As1	33	34.078	−1.078
As2		34.067	−1.067
As3		34.049	−1.049
As4		34.125	−1.125

Table S29. Overlap population and interatomic distances of Na_3InAs_2.

Atom A	Atom B	r_{AB} / Å	overlap
Na1	As4	2.851	0.047
	As3	2.899	0.047
	As2	2.927	0.055
	As2	2.943	0.028
	Na3	3.316	0.006
	In2	3.323	0.003
	Na5	3.333	0.005
	Na5	3.335	0.004
	Na3	3.391	0.003
	Na6	3.414	0.005
	In1	3.45	0.002
	Na2	3.613	0.001
Na2	As1	2.969	0.051
	As3	3.073	0.026
	As1	3.127	0.035
	As3	3.226	0.03
	In1	3.267	0.006
	Na4	3.302	0.006
	In2	3.344	0.006
	Na4	3.467	0.004
	In1	3.495	0.006
	Na2	3.518	0.005
	As4	3.568	0.014
	Na3	3.625	0.002
	Na6	3.909	0.001
	Na6	3.965	0.0
Na3	As4	3.094	0.033
	As3	3.104	0.053
	As1	3.158	0.021
	As2	3.159	0.033
	In2	3.339	0.006
	Na4	3.353	0.005
	As2	3.428	0.024
	In1	3.457	0.007
	In2	3.607	0.003
	Na6	3.637	0.002
	Na5	3.894	0.001
Na4	As3	2.856	0.045
	As4	2.881	0.056
	As1	2.908	0.06
	As1	2.927	0.045
	Na6	3.19	0.006
	Na6	3.42	0.003
	In1	3.423	0.002
	Na5	3.424	0.003
	Na4	3.443	0.003
Na5	As4	3.01	0.04
	As4	3.078	0.036
	As2	3.141	0.034
	As1	3.267	0.016
	As2	3.372	0.025
	In2	3.415	0.01
	In1	3.464	0.007
	In2	3.585	0.004
	Na5	3.666	0.0
	Na6	3.745	0.002
Na6	As2	2.929	0.032
	As3	3.138	0.035
	As4	3.279	0.035
	As3	3.281	0.018
	As1	3.291	0.029
	In1	3.414	0.006
	In1	3.48	0.006
	In2	3.518	0.006
In1	As2	2.682	0.253
	As3	2.689	0.268
	As1	2.733	0.248
	As3	2.763	0.242
	In2	4.403	−0.019
	As3	4.748	0.005
	In1	4.774	−0.02
	In2	4.877	−0.01
In2	As4	2.671	0.245
	As1	2.707	0.253
	As2	2.707	0.248
	As4	2.724	0.227
	In2	3.411	−0.066
	As4	4.741	0.006
As1	As4	4.311	−0.024
	As2	4.412	−0.018
	As3	4.466	−0.016
	As3	4.477	−0.015
	As4	4.512	−0.005
	As2	4.545	−0.014
	As4	4.637	−0.016
	As3	4.673	−0.003
	As1	4.711	−0.004
As2	As4	4.232	−0.021
	As3	4.447	−0.018
	As4	4.516	−0.02
	As3	4.563	−0.017
	As2	4.612	−0.004
	As4	4.916	−0.003
	As4	4.958	−0.002
As3	As3	4.222	−0.018
	As4	4.632	−0.002
	As4	4.837	−0.003
As4	As4	4.18	−0.038
	As4	4.861	−0.001

6 Structure type C

6.1 K_3AlP_2[10]

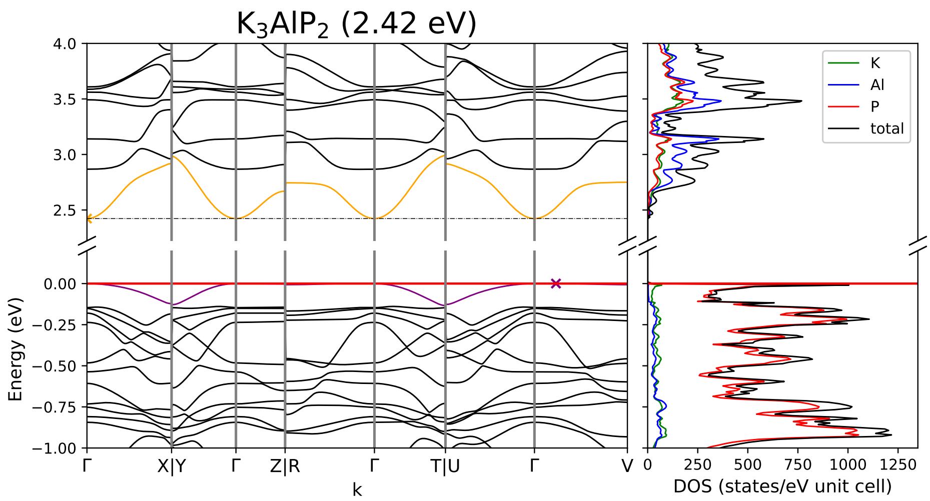

Figure S53. Band structure and DOS of K_3AlP_2.

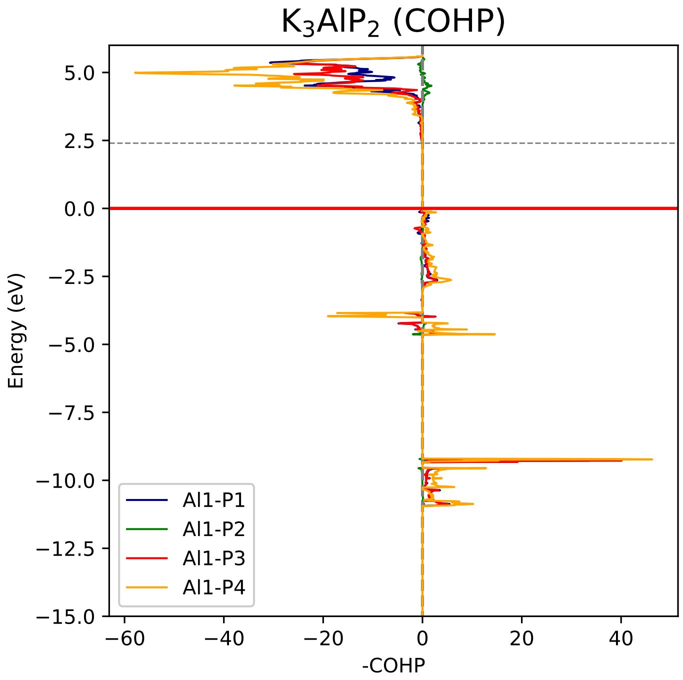

Figure S54. COHP of Al1-P interactions in K_3AlP_2.

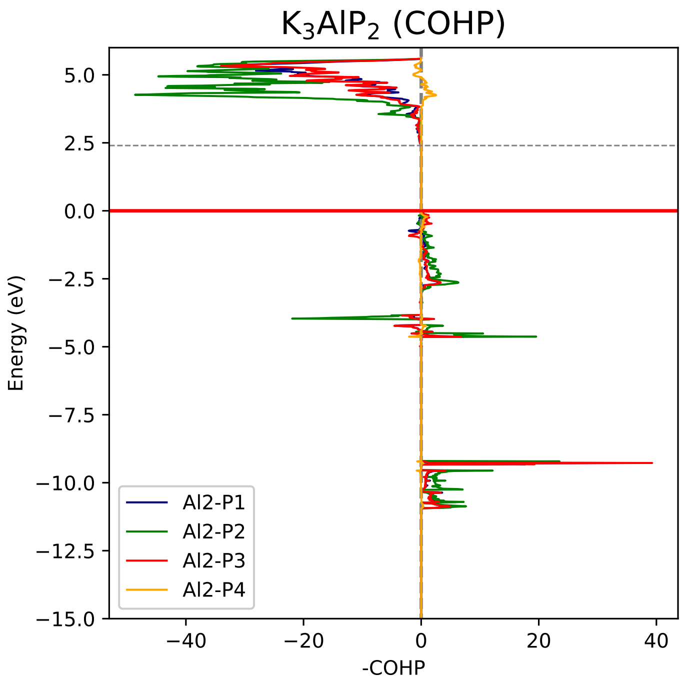

Figure S55. COHP of Al2-P interactions in K_3AlP_2.

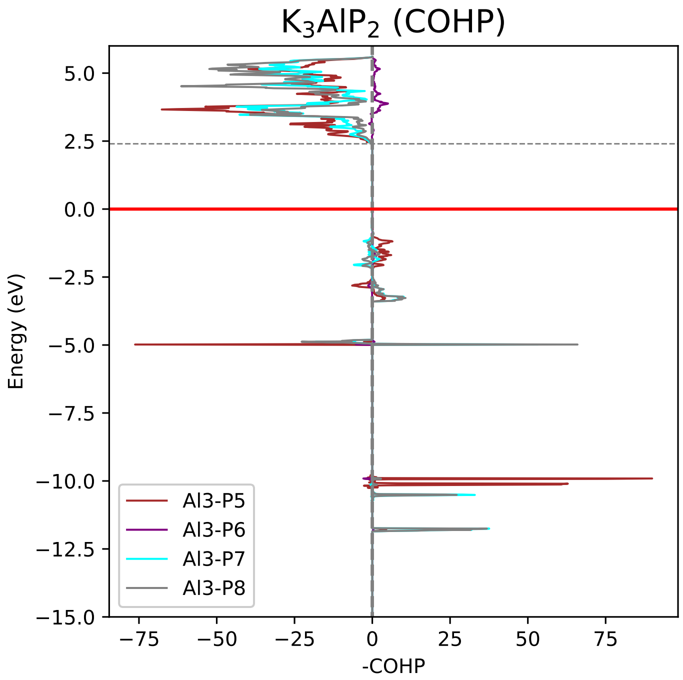

Figure S56. COHP of Al3-P interactions in K_3AlP_2.

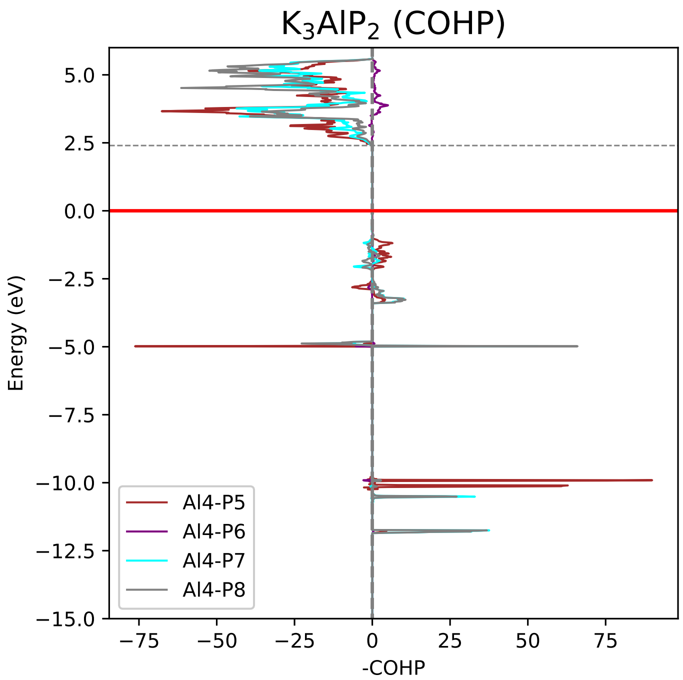

Figure S57. COHP of Al4-P interactions in K_3AlP_2.

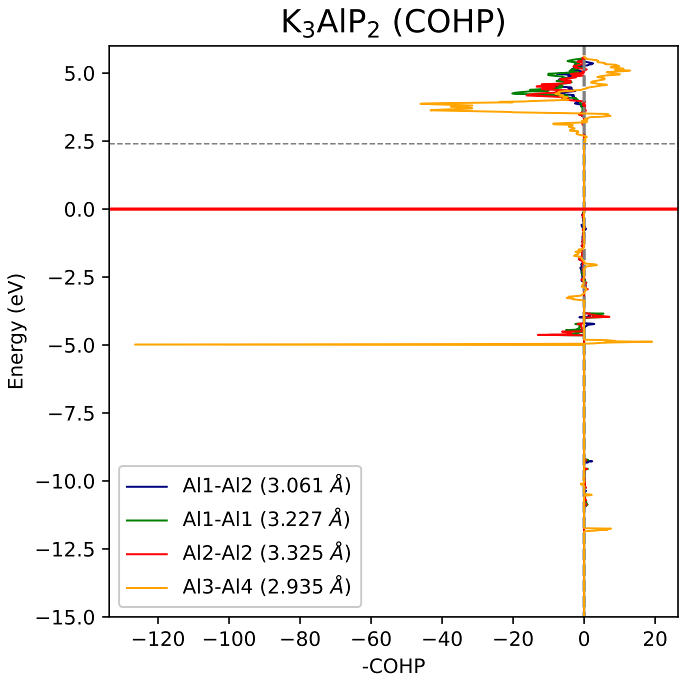

Figure S58. COHP of Al-Al interactions in K_3AlP_2.

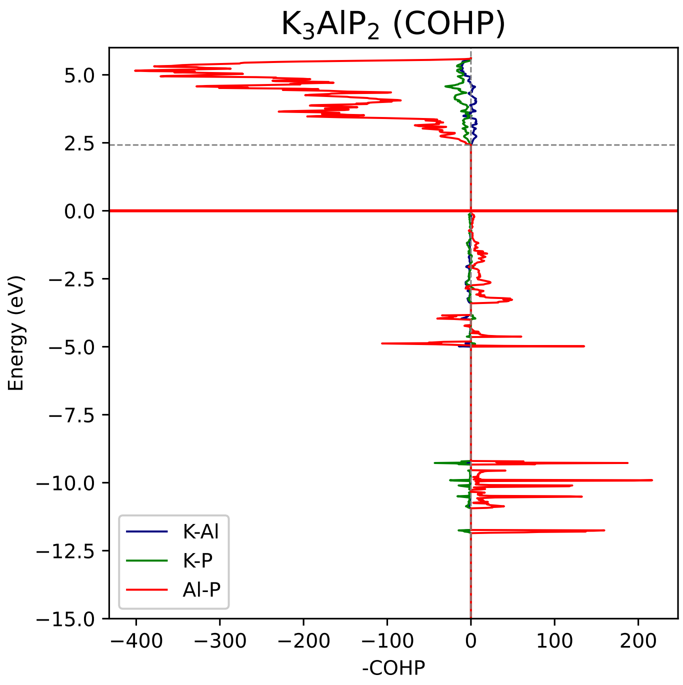

Figure S59. COHP of K-Al, K-P and Al-P interactions in K_3AlP_2.

Table S30. Overlap population and interatomic distances of K_3AlP_2.

Atom A	Atom B	r_{AB} / Å	overlap
K1	K4	3.112	−0.006
	P8	3.17	−0.001
	Al2	3.269	−0.015
	P1	3.309	0.009
	Al2	3.325	−0.013
	K7	3.34	−0.003
	P2	3.38	0.004
	P2	3.397	0.005
	P5	3.446	0.013
	P3	3.522	0.014
	Al3	3.567	−0.007
	K9	3.65	0.0
	K6	3.766	0.001
	K3	3.871	0.001
	K8	3.998	0.001
K2	P3	3.302	0.011
	Al1	3.338	−0.011
	P4	3.398	0.009
	Al1	3.418	−0.008
	P6	3.494	0.02
	P1	3.499	0.018
	K5	3.532	0.0
	P4	3.577	0.009
	P7	3.592	0.01
	K10	3.629	0.001
	K8	3.666	0.0
	Al4	3.769	−0.001
	K9	3.864	0.001
	K6	3.934	0.002
K3	P3	3.164	0.013
	P2	3.217	0.023
	P5	3.362	0.025
	P4	3.365	0.025
	K4	3.478	−0.001
	Al2	3.614	−0.009
	Al1	3.739	−0.004
	K8	3.884	0.002
	K7	3.956	0.001
K4	P3	3.076	0.014
	P4	3.092	0.026
	P2	3.272	0.019
	P5	3.338	0.024
	Al2	3.681	−0.006
	K10	3.694	0.0
	K6	3.731	0.001
	K5	3.858	0.002
K5	P1	3.217	0.018
	P6	3.235	0.026
	P7	3.364	0.022
	P4	3.397	0.032
	K10	3.478	0.0
	Al1	3.902	−0.001
	K12	3.984	0.001
K6	P1	3.227	0.016
	P2	3.246	0.024
	P4	3.469	0.027
	P2	3.571	0.036
	Al2	3.721	−0.006
	Al1	3.825	−0.003
	P5	3.959	0.02
	K7	3.974	0.002
K7	P6	3.155	0.025
	P2	3.265	0.027
	P8	3.356	0.02
	P1	3.391	0.022
	K10	3.616	0.0
	K11	3.899	0.001
	Al2	3.912	−0.001
K8	P6	3.281	0.02
	P3	3.33	0.041
	Al3	3.397	−0.001
	Al4	3.529	−0.002
	P8	3.561	0.013
	P7	3.789	0.013
	Al4	3.89	0.001
	P7	3.941	0.008
K9	P1	3.199	0.038
	P5	3.427	0.022
	P7	3.465	0.014
	Al3	3.498	−0.002
	P8	3.524	0.016
	Al4	3.591	0.002
	Al3	3.884	0.002
	K11	3.962	0.001
K10	K10	3.278	−0.001
	P1	3.424	0.016
	P3	3.445	0.013
	P4	3.495	0.029
	Al1	3.636	−0.003
	P3	3.655	0.022
	P6	3.691	0.021
	Al2	3.736	−0.002
K11	P5	3.231	0.026
	K11	3.253	−0.002
	K12	3.298	−0.002
	P8	3.334	0.019
	P6	3.4	0.029
	P8	3.563	0.011
	Al4	3.84	0.0
	Al3	3.846	0.004
	Al4	3.953	0.005
K12	P7	3.219	0.024
	P5	3.302	0.026
	P6	3.406	0.027
	P7	3.626	0.012
	Al3	3.677	−0.001
	K12	3.715	0.0
	Al4	3.811	0.005
Al1	P4	2.43	0.324
	P1	2.432	0.308
	P3	2.441	0.303
	P4	2.454	0.302
	Al2	3.061	−0.013
	Al1	3.227	−0.014
Al2	P1	2.434	0.308
	P2	2.443	0.33
	P3	2.453	0.309
	P2	2.512	0.285
	Al2	3.325	−0.029
Al3	P5	2.251	0.505
	P7	2.359	0.333
	P8	2.383	0.308
	Al4	2.935	−0.032
Al4	P6	2.232	0.503
	P8	2.324	0.333
	P7	2.374	0.307
P1	P3	3.689	−0.075
	P4	4.098	−0.028
	P2	4.123	−0.026
	P4	4.162	−0.025
	P2	4.18	−0.027
	P6	4.726	0.0
P2	P2	3.673	−0.097
	P3	4.166	−0.025
	P3	4.234	−0.024
P3	P4	4.124	−0.029
	P4	4.124	−0.025
	P6	4.836	0.0
P4	P4	3.666	−0.093
P5	P8	4.119	−0.035
	P7	4.218	−0.027
P6	P8	4.075	−0.034
	P7	4.166	−0.03
P7	P8	3.694	−0.096

Table S31. Partial charges for each atom position in K_3AlP_2.

Atom	Z	charge	part charge
K1	19	18.218	0.782
K2		18.229	0.771
K3		18.256	0.744
K4		18.282	0.718
K5		18.259	0.741
K6		18.286	0.714
K7		18.267	0.733
K8		18.25	0.75
K9		18.255	0.745
K10		18.256	0.744
K11		18.256	0.744
K12		18.251	0.749
Al1	13	12.755	0.245

Atom	Z	charge	partialcharge
Al2		12.807	0.193
Al3		12.975	0.025
Al4		12.941	0.059
P1	15	16.211	−1.211
P2		16.219	−1.219
P3		16.212	−1.212
P4		16.193	−1.193
P5		16.256	−1.256
P6		16.263	−1.263
P7		16.054	−1.054
P8		16.05	−1.05

6.2 Rb_3InP_2[11]

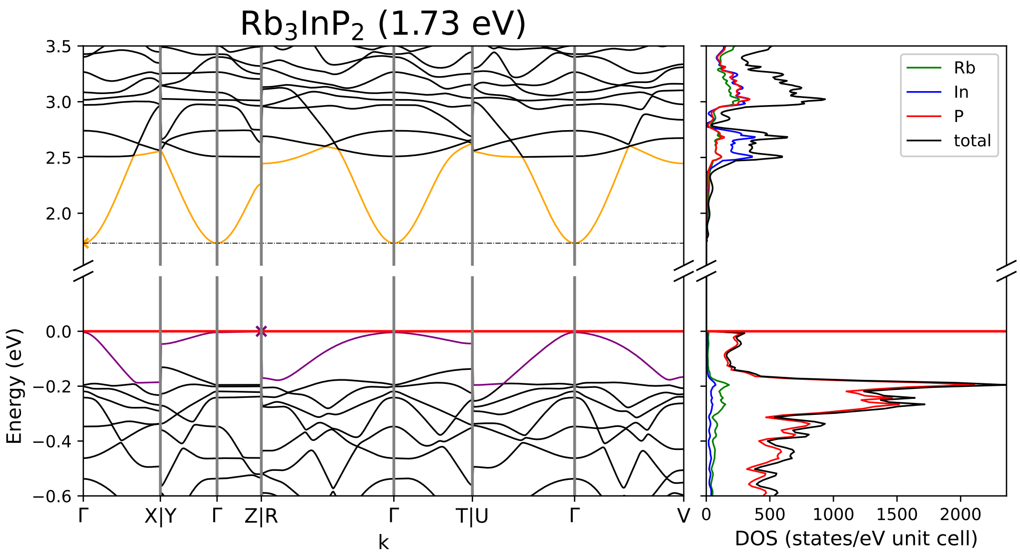

Figure S60. Band structure and DOS of Rb_3InP_2.

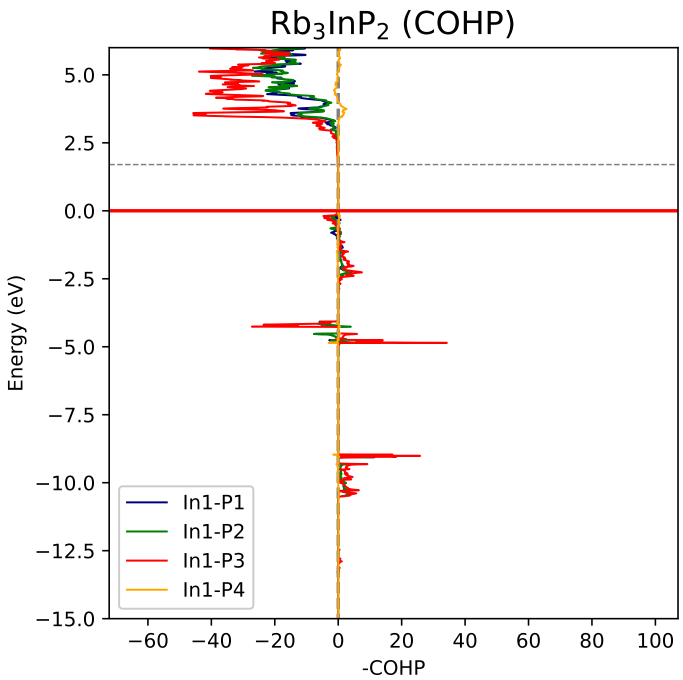

Figure S61. COHP of In1-P interactions in Rb_3InP_2.

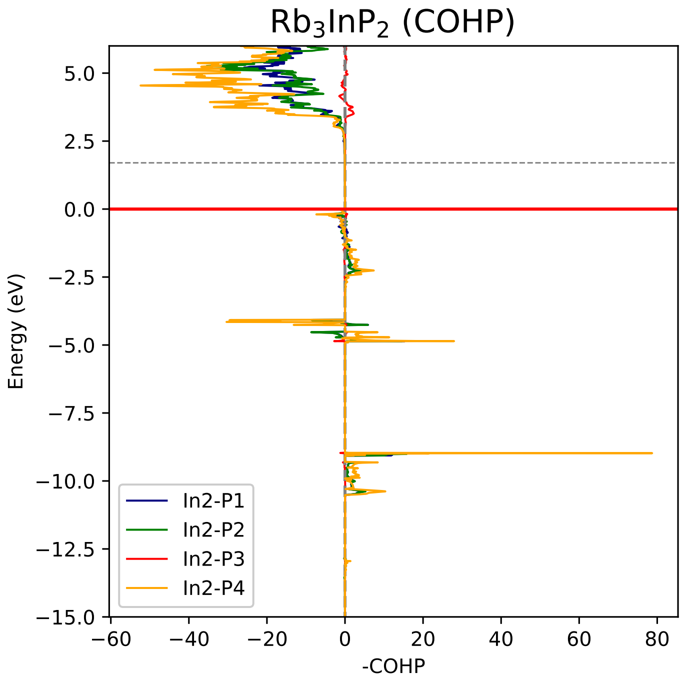

Figure S62. COHP of In2-P interactions in Rb_3InP_2.

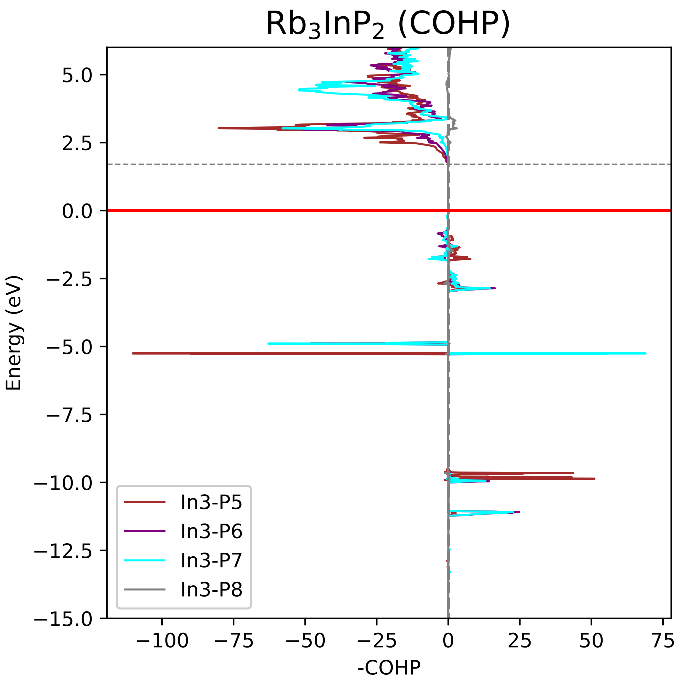

Figure S63. COHP of In3-P interactions in Rb_3InP_2.

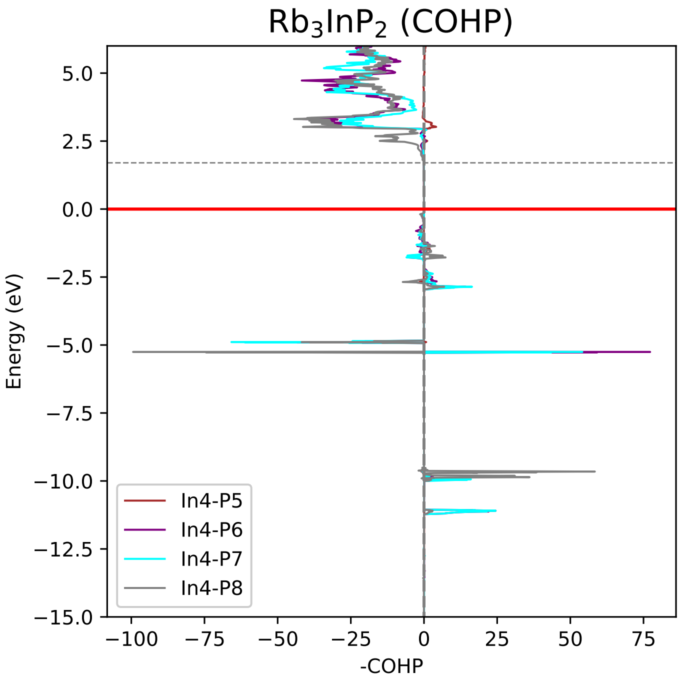

Figure S64. COHP of In4-P interactions in Rb_3InP_2.

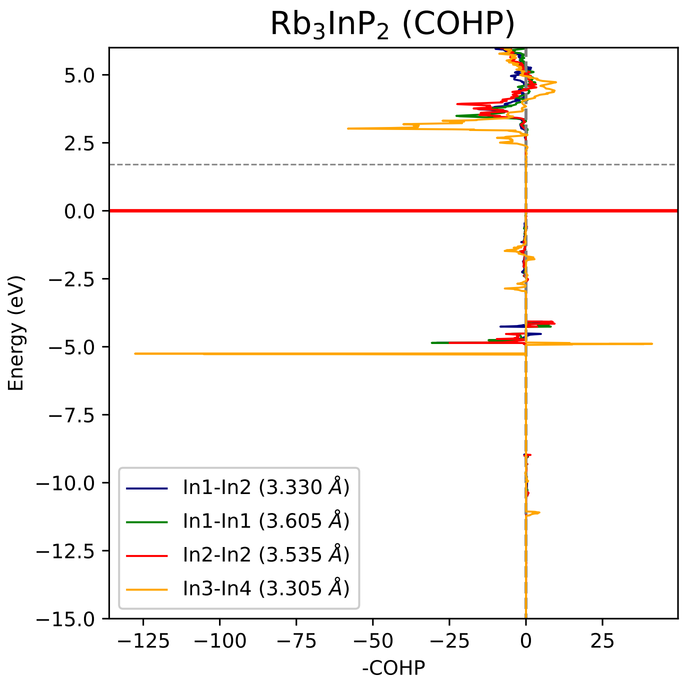

Figure S65. COHP of In-In interactions in Rb_3InP_2.

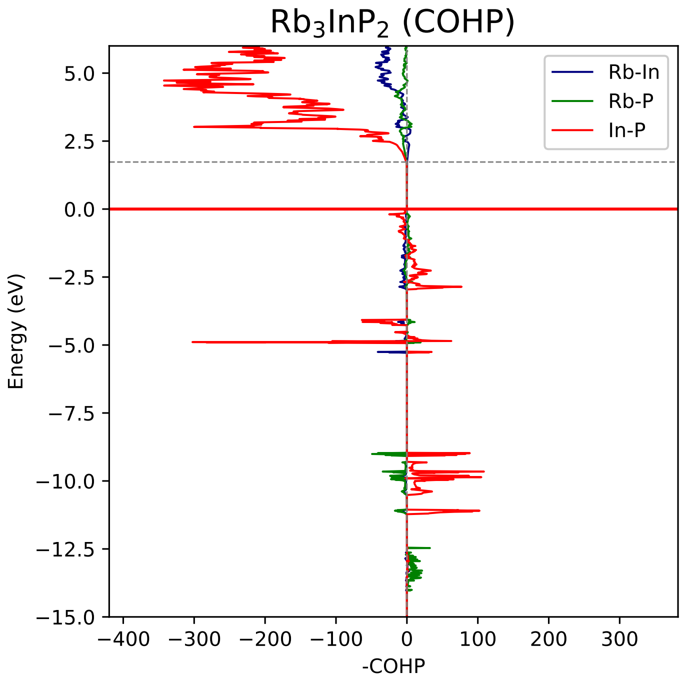

Figure S66. COHP of Rb-In, Rb-P and In-P interactions in Rb_3InP_2.

Table S32. Overlap population and interatomic distances of Rb_3InP_2.

Atom A	Atom B	r_{AB} / Å	overlap
In1	P2	2.634	0.259
	P3	2.647	0.269
	P1	2.649	0.255
	P3	2.71	0.244
	In2	3.33	−0.042
	Rb1	3.465	−0.021
	Rb1	3.54	−0.016
	In1	3.605	−0.059
	Rb5	3.835	−0.012
	Rb2	3.883	−0.011
	Rb10	3.964	−0.004
	Rb6	3.992	−0.007
In2	P2	2.631	0.257
	P4	2.633	0.264
	P4	2.646	0.254
	P1	2.648	0.253
	In2	3.535	−0.042
	Rb3	3.545	−0.013
	Rb3	3.646	−0.01
	Rb10	3.922	−0.004
	Rb5	3.986	−0.006
In3	P5	2.434	0.415
	P6	2.56	0.296
	P7	2.596	0.272
	In4	3.305	−0.096
	Rb4	3.66	−0.006
	Rb1	3.729	−0.016
	Rb7	3.743	−0.007
	Rb12	3.791	−0.01
	Rb7	3.981	−0.003
In4	P8	2.414	0.391
	P7	2.525	0.295
	P6	2.584	0.271
	Rb4	3.801	−0.008
	Rb7	3.884	−0.003
	Rb3	3.924	−0.005
	Rb11	3.927	−0.008
Rb1	P7	3.341	0.003
	Rb2	3.357	−0.013
	P2	3.518	0.012
	Rb8	3.545	−0.007
	P3	3.596	0.006
	P3	3.6	0.008
	P5	3.663	0.013
	P1	3.857	0.013
	Rb7	3.89	−0.001
Rb2	P4	3.253	0.028
	P1	3.287	0.021
	P3	3.477	0.026
	P5	3.512	0.028
	Rb5	3.755	−0.003
	Rb10	3.929	−0.001
Rb3	P1	3.562	0.014
	P4	3.603	0.013
	Rb9	3.751	−0.002
	P8	3.76	0.019
	P6	3.777	0.012
	P4	3.784	0.012
	P2	3.823	0.017
	Rb10	3.824	−0.001
	Rb4	3.943	0.0
Rb4	P8	3.448	0.027
	P1	3.506	0.045
	P7	3.858	0.013
Rb5	P1	3.396	0.022
	P3	3.413	0.03
	P5	3.555	0.026
	P4	3.586	0.029
Rb6	P3	3.45	0.031
	P2	3.459	0.024
	P4	3.725	0.029
	P3	3.816	0.033
Rb7	P2	3.373	0.044
	P5	3.66	0.024
	P6	3.677	0.016
	P7	3.746	0.018
Rb8	P8	3.354	0.034
	P3	3.486	0.033
	P7	3.55	0.025
	P2	3.612	0.027
	Rb10	3.816	−0.002
Rb9	P8	3.372	0.027
	P2	3.374	0.025
	P6	3.551	0.029
	P4	3.624	0.034
	Rb10	3.749	−0.003
Rb10	Rb10	3.533	−0.005
	P2	3.644	0.018
	P1	3.66	0.018
	P4	3.692	0.031
	P1	3.798	0.024
	P8	3.835	0.022
Rb11	P5	3.381	0.032
	Rb11	3.483	−0.008
	P7	3.507	0.028
	Rb12	3.545	−0.006
	P8	3.565	0.03
	P7	3.696	0.012
Rb12	P6	3.383	0.033
	P5	3.523	0.029
	P8	3.561	0.034
	P6	3.811	0.011
	Rb12	3.879	−0.002
P1	P2	3.971	−0.042
	P4	4.463	−0.017
	P4	4.489	−0.016
	P3	4.531	−0.014
	P3	4.585	−0.014
P2	P3	4.468	−0.017
	P4	4.476	−0.017
	P3	4.496	−0.016
	P4	4.497	−0.016
	P8	4.874	0.0
P3	P3	3.963	−0.053
P4	P4	3.921	−0.05
P5	P7	4.471	−0.02
	P6	4.622	−0.015
P6	P7	3.925	−0.065
	P8	4.542	−0.017
P7	P8	4.459	−0.02

Table S33. Partial charges for each atom position in Rb_3InP_2.

Atom	Z	charge	part charge
In1	21	20.945	0.055
In2		20.891	0.109
In3		21.155	−0.155
In4		21.13	−0.13
Rb1	9	8.26	0.74
Rb2		8.346	0.654
Rb3		8.269	0.731
Rb4		8.294	0.706
Rb5		8.312	0.688
Rb6		8.339	0.661
Rb7		8.3	0.7
Rb8		8.33	0.67
Rb9		8.317	0.683

Atom	Z	charge	partialcharge
Rb10		8.295	0.705
Rb11		8.304	0.696
Rb12		8.306	0.694
P1	15	16.055	−1.055
P2		16.052	−1.052
P3		16.072	−1.072
P4		16.057	−1.057
P5		16.072	−1.072
P6		15.918	−0.918
P7		15.918	−0.918
P8		16.064	−1.064

6.3 K_3AlAs_2[12]

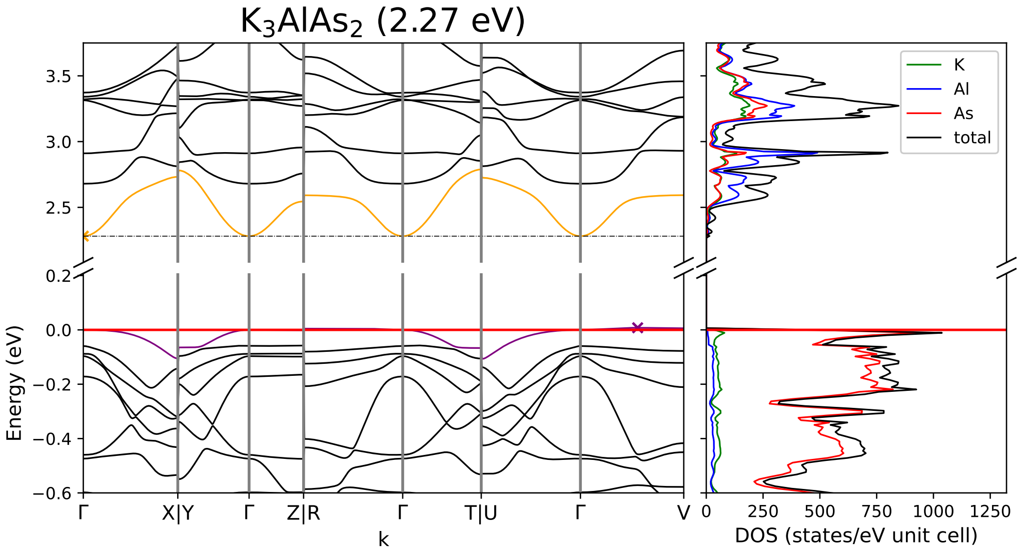

Figure S67. Band structure and DOS of K_3AlAs_2.

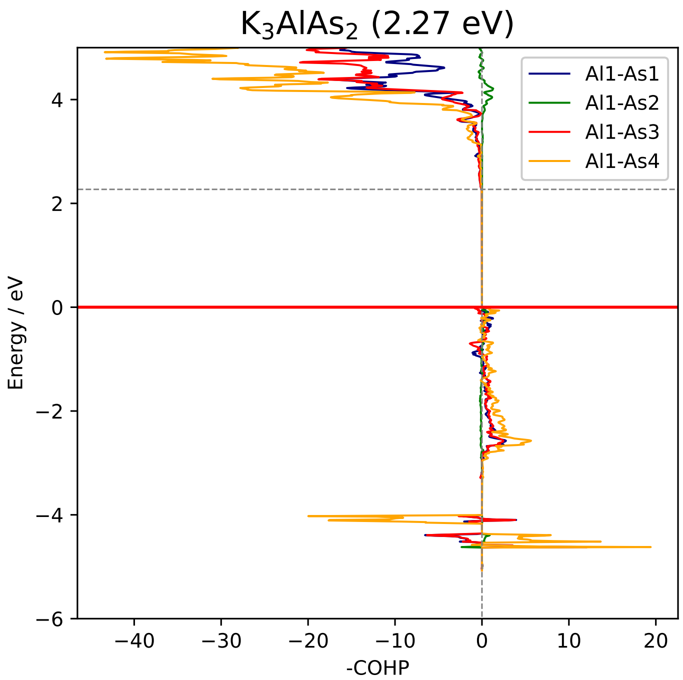

Figure S68. COHP of Al1-As interactions in K_3AlAs_2.

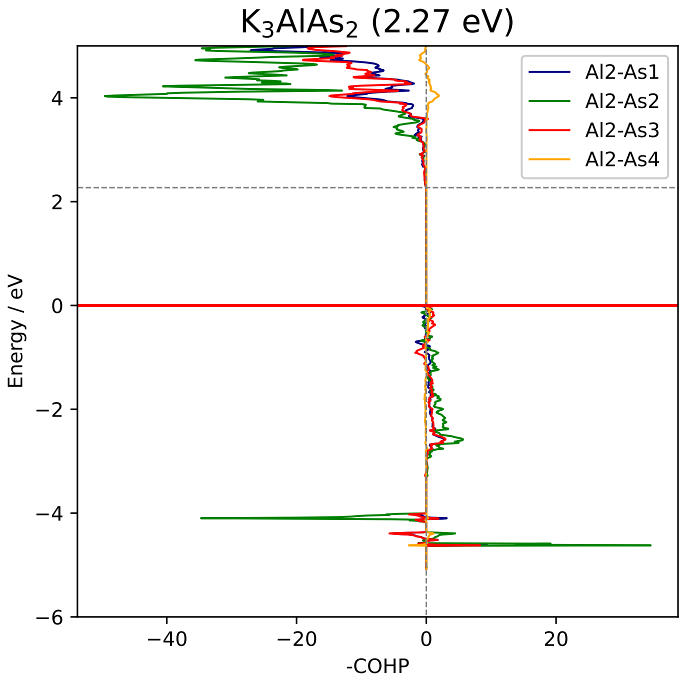

Figure S69. COHP of Al2-As interactions in K_3AlAs_2.

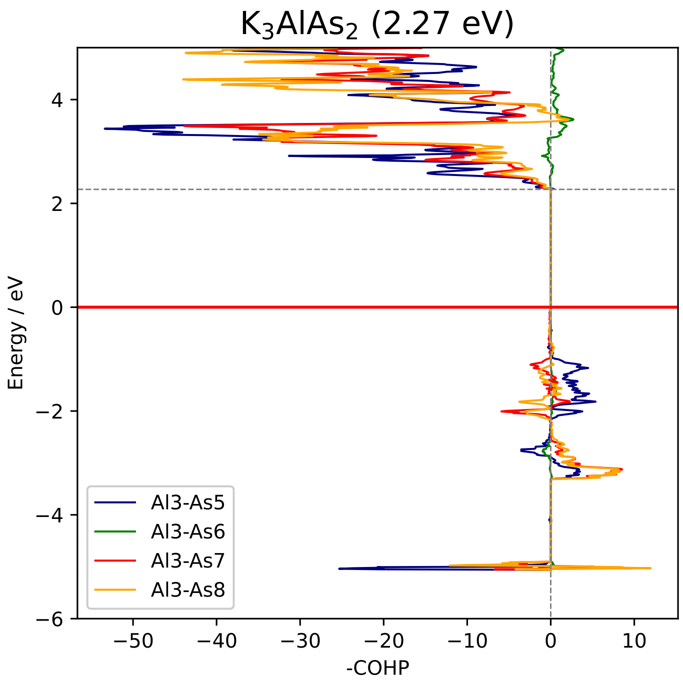

Figure S70. COHP of Al3-As interactions in K_3AlAs_2.

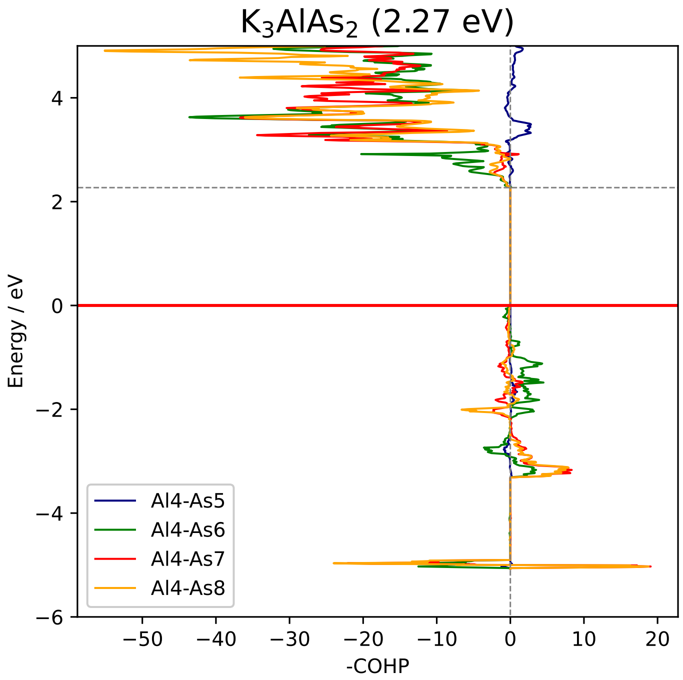

Figure S71. COHP of Al4-P interactions in K_3AlP_2.

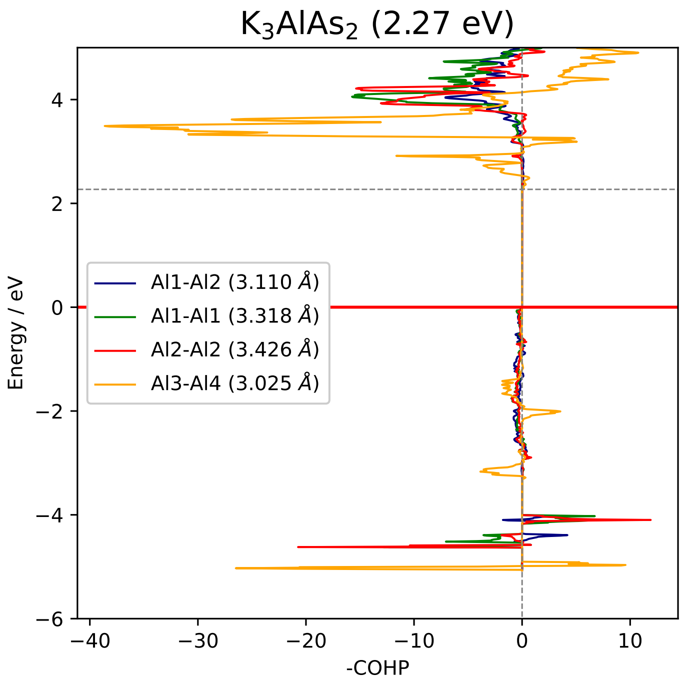

Figure S72. COHP of Al-Al interactions in K_3AlAs_2.

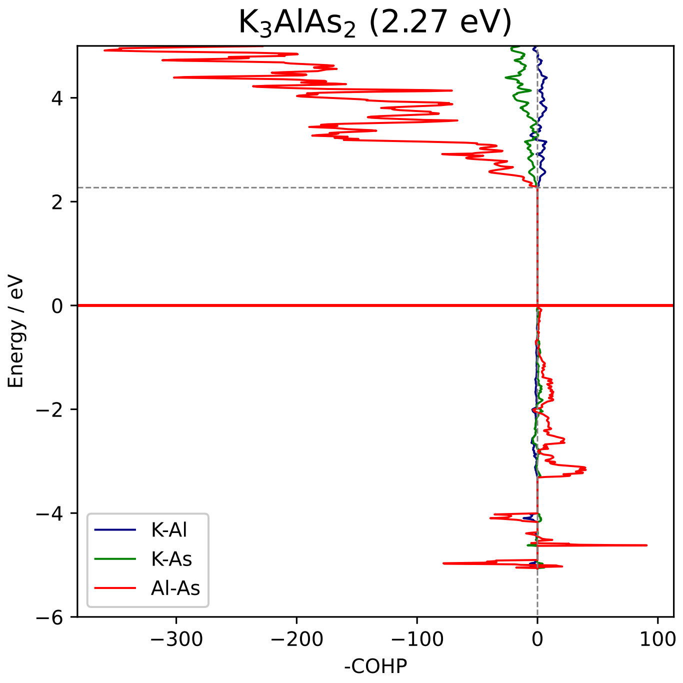

Figure S73. COHP of K-Al, K-As and Al-As interactions in K_3AlAs_2.

Table S34. Overlap population and interatomic distances of K_3AlAs_2.

Atom A	Atom B	r_{AB} / Å	overlap
K1	K4	3.174	−0.005
	As8	3.222	0.002
	Al2	3.321	−0.01
	As1	3.364	0.012
	Al2	3.373	−0.008
	As2	3.451	0.009
	As2	3.459	0.01
	K7	3.471	−0.001
	As5	3.558	0.017
	As3	3.615	0.017
K2	As3	3.379	0.015
	Al1	3.399	−0.006
	Al1	3.472	−0.004
	As4	3.496	0.013
	As1	3.545	0.021
	As6	3.549	0.025
	K5	3.627	0.001
	As4	3.631	0.013
	K10	3.718	0.002
	As7	3.731	0.012
K3	As3	3.244	0.022
	As2	3.274	0.026
	As4	3.424	0.029
	As5	3.452	0.029
	K4	3.541	0.0
	Al2	3.686	−0.005
	Al1	3.828	−0.002
	K8	3.917	0.003
	K7	3.977	0.002
K4	As3	3.146	0.019
	As4	3.155	0.033
	As2	3.326	0.024
	As5	3.398	0.029
	Al2	3.757	−0.004
	K6	3.772	0.002
	K10	3.8	0.001
	K5	3.942	0.002
K5	As6	3.288	0.029
	As1	3.29	0.023
	As7	3.446	0.027
	As4	3.449	0.037
	K10	3.546	0.001
	Al1	3.999	0.0
K6	As2	3.289	0.028
	As1	3.339	0.023
	As4	3.519	0.03
	As2	3.632	0.037
	Al2	3.81	−0.003
	Al1	3.924	−0.001
	As5	3.928	0.023
	K7	3.943	0.002
K7	As6	3.235	0.032
	As2	3.334	0.033
	As8	3.393	0.022
	As1	3.528	0.025
	K10	3.751	0.001
K8	As6	3.372	0.026
	As3	3.404	0.045
	Al3	3.459	0.002
	As8	3.578	0.017
	Al4	3.581	0.002
	As7	3.923	0.013
	Al4	3.932	0.003
	As7	3.983	0.01
K9	As1	3.25	0.042
	As5	3.453	0.027
	As7	3.557	0.018
	Al3	3.562	0.002
	Al4	3.611	0.005
	As8	3.689	0.018
	K11	3.956	0.002
	Al3	3.999	0.004
K10	K10	3.375	0.0
	As1	3.473	0.02
	As3	3.492	0.018
	As4	3.518	0.033
	Al1	3.696	−0.002
	As3	3.703	0.023
	As6	3.758	0.023
K11	As5	3.313	0.032
	K11	3.337	0.0
	K12	3.352	−0.001
	As8	3.399	0.026
	As6	3.449	0.036
	As8	3.739	0.011
	Al3	3.87	0.006
	Al4	3.993	0.002
K12	As7	3.304	0.032
	As5	3.393	0.031
	As6	3.505	0.031
	As7	3.631	0.014
	Al3	3.731	0.0
	Al4	3.843	0.007
	K12	3.924	0.001
Al1	As1	2.517	0.3
	As4	2.524	0.309
	As3	2.533	0.294
	As4	2.539	0.296
	Al2	3.11	−0.004
	Al1	3.318	−0.016
Al2	As1	2.523	0.3
	As2	2.532	0.317
	As3	2.552	0.3
	As2	2.606	0.278
	Al2	3.426	−0.025
Al3	As5	2.347	0.482
	As7	2.457	0.323
	As8	2.481	0.3
	Al4	3.025	−0.023
Al4	As6	2.321	0.484
	As8	2.412	0.327
	As7	2.469	0.296
As1	As3	3.862	−0.059
As2	As2	3.829	−0.075
As4	As4	3.823	−0.073
As7	As8	3.858	−0.078

Table S35. Partial charges for each atom position in K_3AlAs_2.

Atom	Z	charge	part charge
K1	19	18.245	0.755
K2		18.257	0.743
K3		18.281	0.719
K4		18.3	0.7
K5		18.277	0.723
K6		18.31	0.69
K7		18.288	0.712
K8		18.275	0.725
K9		18.278	0.722
K10		18.279	0.721
K11		18.283	0.717
K12		18.274	0.726
Al1	13	12.638	0.362

Atom	Z	charge	partialcharge
Al2		12.705	0.295
Al3		12.868	0.132
Al4		12.82	0.18
As1	33	34.232	−1.232
As2		34.233	−1.233
As3		34.23	−1.23
As4		34.219	−1.219
As5		34.253	−1.253
As6		34.261	−1.261
As7		34.1	−1.1
As8		34.095	−1.095

7 Structure type D

Cs_3InP_2[13]

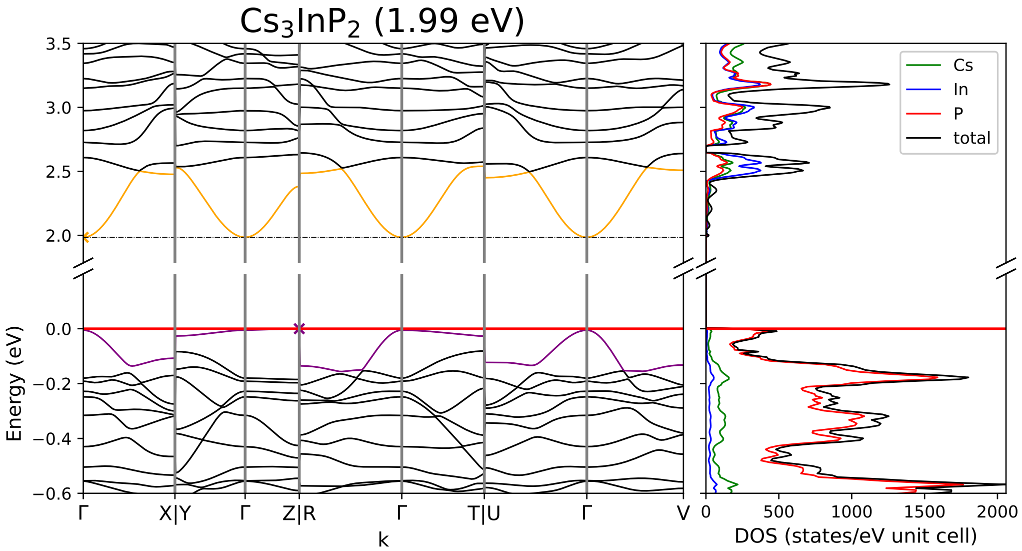

Figure S74. Band structure and DOS of Cs_3InP_2.

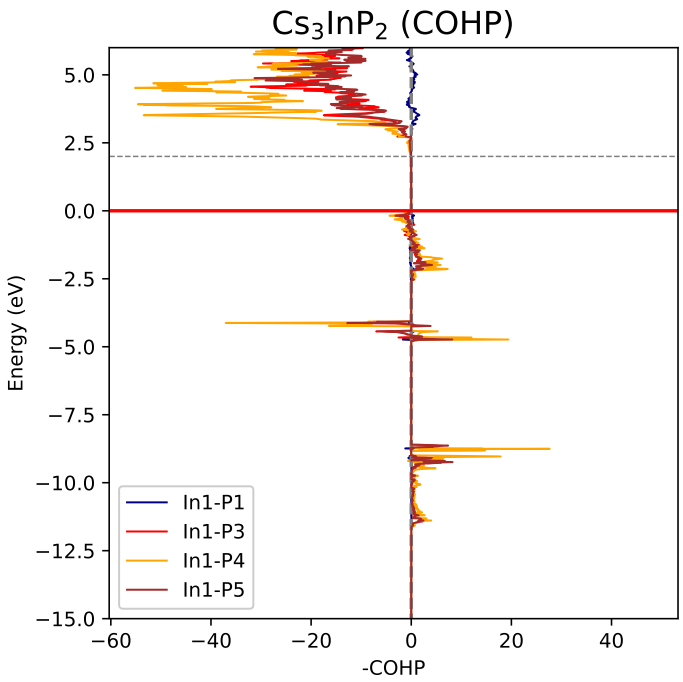

Figure S75. COHP of In1-P interactions in Cs_3InP_2.

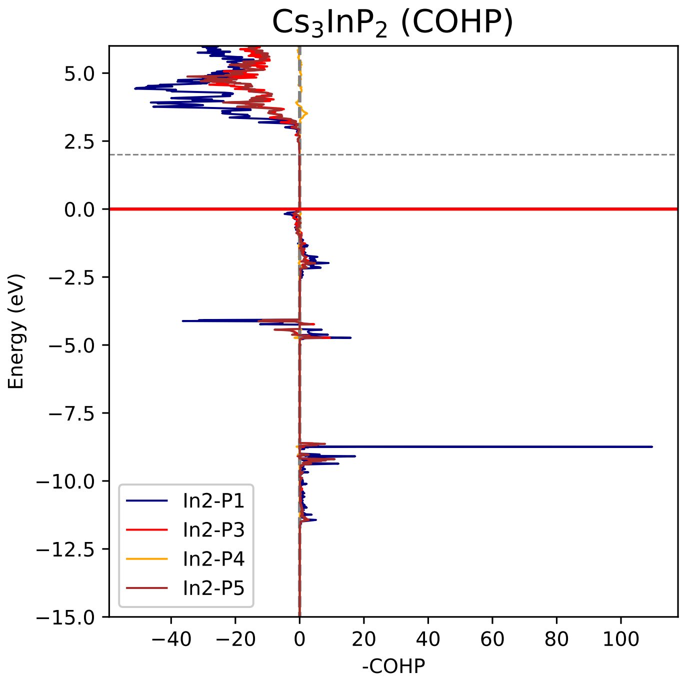

Figure S76. COHP of In2-P interactions in Cs_3InP_2.

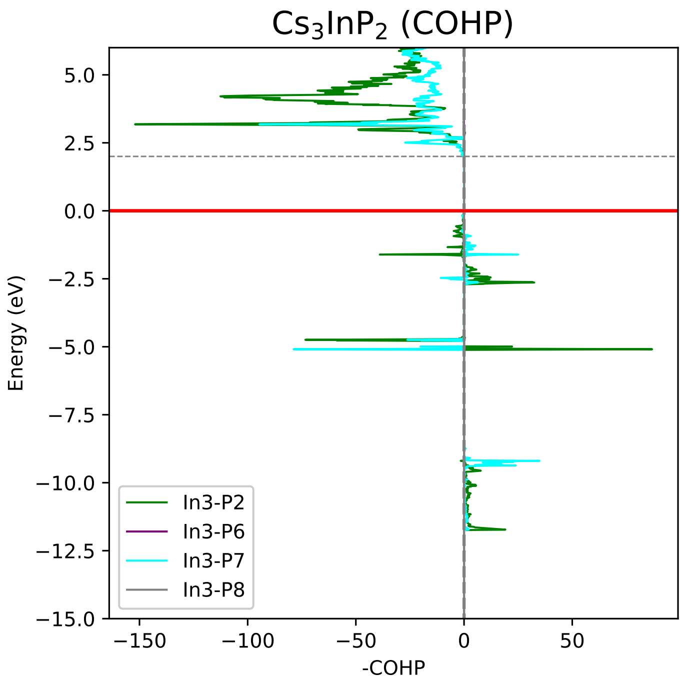

Figure S77. COHP of In3-P interactions in Cs_3InP_2.

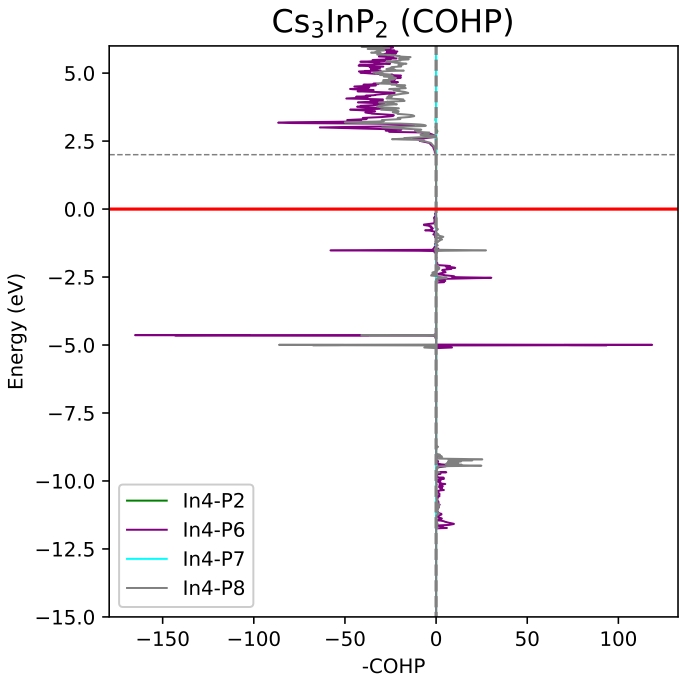

Figure S78. COHP of In4-P interactions in Cs_3InP_2.

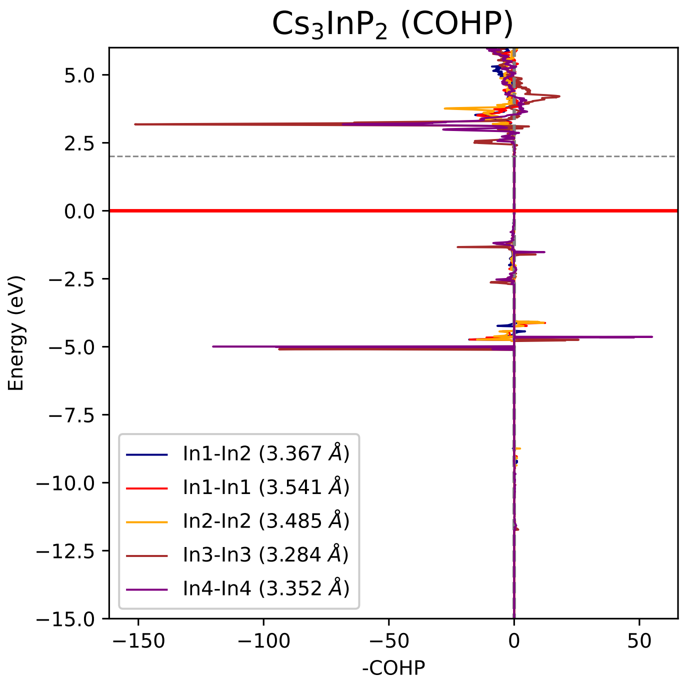

Figure S79. COHP of In-In interactions in Cs_3InP_2.

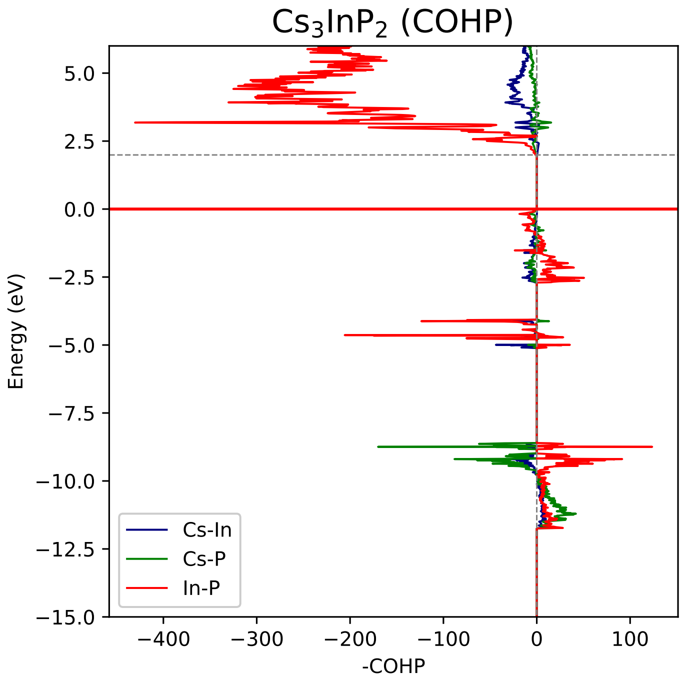

Figure S80. COHP of Cs-In, Cs-P and In-P interactions in Cs_3InP_2.

Table S36. Overlap population and interatomic distances of Cs_3InP_2.

Atom A	Atom B	r_{AB} / Å	overlap
Cs1	P5	3.482	0.009
	P6	3.492	0.004
	In1	3.577	−0.019
	Cs7	3.634	0.006
	P4	3.702	0.008
	In1	3.769	−0.009
	Cs11	3.879	0.005
	P4	3.881	0.007
	P8	3.911	0.014
	In4	3.946	−0.011
	P3	4.006	0.013
Cs2	P3	3.535	0.012
	P1	3.623	0.009
	In2	3.655	−0.014
	P2	3.771	0.01
	In2	3.774	−0.009
	Cs6	3.877	0.006
	P7	3.9	0.018
	P5	3.942	0.016
	Cs12	3.959	0.006
	Cs4	3.975	0.005
Cs3	P3	3.438	0.014
	P4	3.516	0.027
	P1	3.548	0.028
	Cs7	3.822	0.006
	In1	3.927	−0.011
	P8	3.93	0.02
	In2	3.997	−0.007
Cs4	P3	3.565	0.04
	P7	3.577	0.025
	P2	3.788	0.017
	In3	3.836	−0.009
	In4	3.858	0.005
	P6	3.969	0.015
Cs5	P5	3.486	0.017
	P4	3.513	0.03
	P1	3.817	0.027
	P4	4.049	0.027
Cs6	P5	3.419	0.022
	P7	3.504	0.024
	P1	3.63	0.031
	P2	3.68	0.026
	Cs12	3.847	0.006
	Cs8	3.992	0.007
Cs7	P3	3.367	0.02
	P1	3.501	0.027
	P4	3.628	0.029
	P8	3.811	0.028
Cs8	P7	3.507	0.027
	P7	3.736	0.032
	P6	3.785	0.022
	In3	3.901	−0.009
	P2	3.927	0.011
Cs9	P5	3.614	0.038
	In4	3.754	−0.007
	P8	3.774	0.025
	P2	3.872	0.014
	P6	3.887	0.019
	In3	3.967	0.002
Cs10	P2	3.512	0.028
	Cs10	3.653	0.007
	P6	3.72	0.019
	P8	3.759	0.035
	In4	3.805	−0.006
	P8	3.889	0.022
Cs11	P7	3.593	0.035
	P6	3.755	0.029
	P8	3.856	0.03
	P4	3.899	0.029
Cs12	P5	3.622	0.013
	P3	3.627	0.013
	Cs12	3.864	0.007
	In2	3.902	−0.006
	In1	3.93	−0.005
	P1	3.999	0.025
	P7	4.04	0.02
	P3	4.295	0.017
	P1	4.405	0.005
	P4	4.491	0.004
	In2	4.913	0.001
In1	P4	2.633	0.275
	P3	2.644	0.254
	P5	2.66	0.261
	P4	2.716	0.244
	In2	3.367	−0.06
	In1	3.541	−0.058
In2	P5	2.632	0.26
	P1	2.644	0.267
	P3	2.656	0.25
	P1	2.661	0.255
	In2	3.485	−0.045
In3	P7	2.431	0.387
	P2	2.548	0.292
	P2	2.587	0.279
	In3	3.284	−0.102
In4	P8	2.444	0.416
	P6	2.545	0.298
	P6	2.616	0.247
	In4	3.352	−0.1
P1	P1	3.999	−0.047
	P3	4.453	−0.016
	P3	4.483	−0.017
	P5	4.489	−0.017
	P5	4.516	−0.016
P2	P2	3.948	−0.068
	P7	4.463	−0.02
	P7	4.58	−0.016
P3	P5	3.963	−0.044
	P4	4.489	−0.016
	P4	4.584	−0.014
P4	P4	4.01	−0.051
	P5	4.48	−0.015
	P5	4.522	−0.016
P6	P6	3.925	−0.064
	P8	4.549	−0.017
	P8	4.563	−0.018

Table S37. Partial charges for each atom position in Cs_3InP_2.

Atom	Z	charge	part charge
Cs1	9	8.239	0.761
Cs2		8.246	0.754
Cs3		8.301	0.699
Cs4		8.288	0.712
Cs5		8.327	0.673
Cs6		8.307	0.693
Cs7		8.334	0.666
Cs8		8.281	0.719
Cs9		8.268	0.732
Cs10		8.292	0.708
Cs11		8.311	0.689
Cs12		8.263	0.737
In1	21	20.944	0.056
In2		20.909	0.091
In3		21.13	−0.13
In4		21.15	−0.15
P1	15	16.081	−1.081
P2		15.947	−0.947
P3		16.091	−1.091
P4		16.079	−1.079
P5		16.077	−1.077
P6		15.95	−0.95
P7		16.089	−1.089
P8		16.096	−1.096

8 Structure type B

Rb_3GaP_2[14]

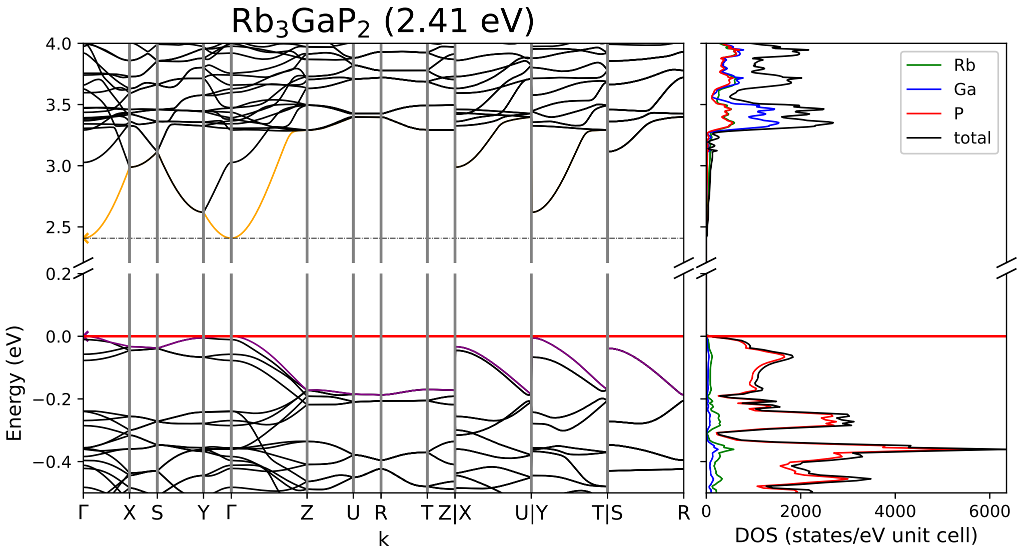

Figure S81. Band structure and DOS of Rb_3GaP_2.

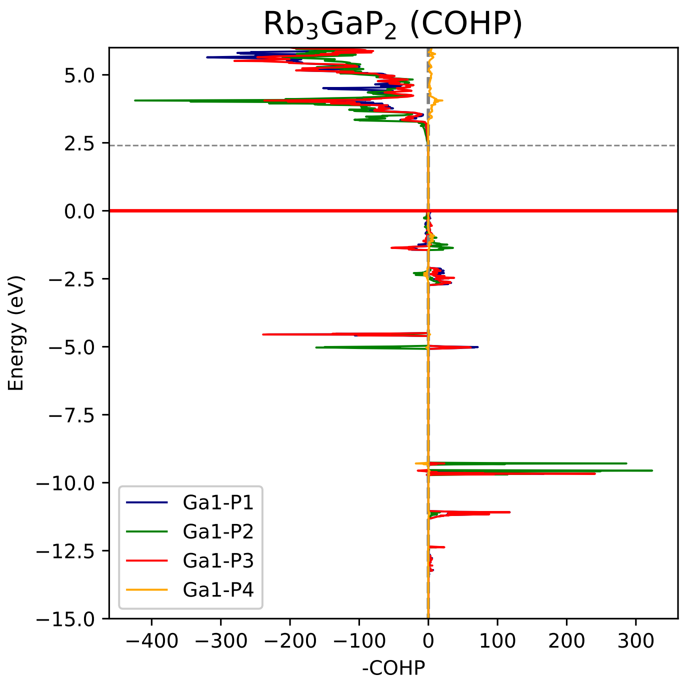

Figure S82. COHP of Ga1-P interactions in Rb_3GaP_2.

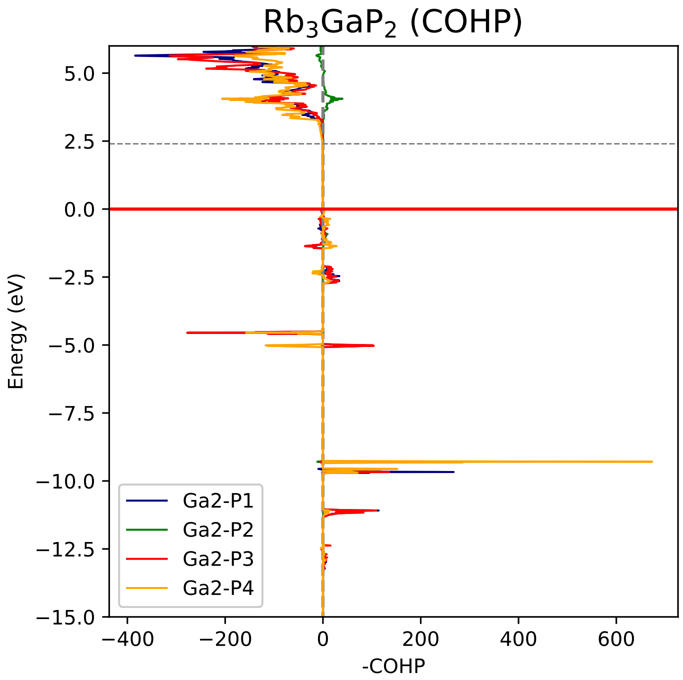

Figure S83. COHP of Ga2-P interactions in Rb_3GaP_2.

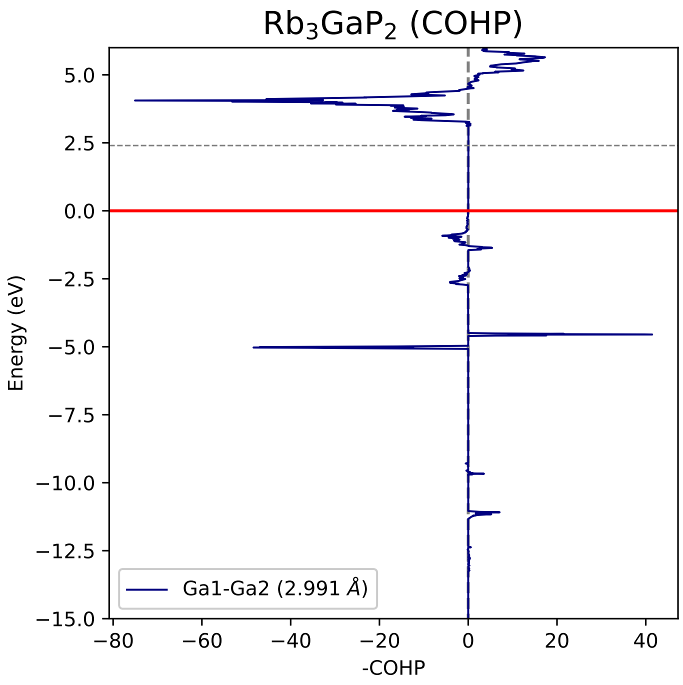

Figure S84. COHP of Ga-Ga interactions in Rb_3GaP_2.

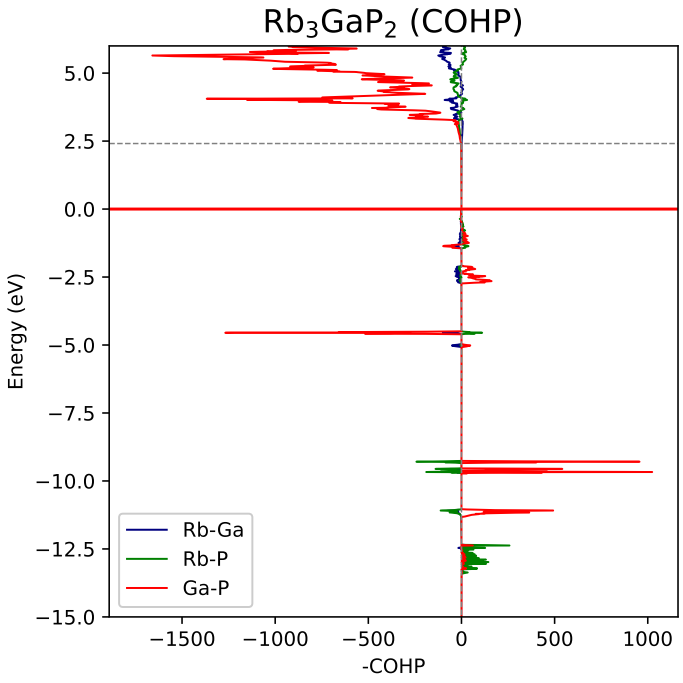

Figure S85. COHP of Rb-Ga, Rb-P and Ga-P interactions in Rb_3GaP_2.

Table S38. Overlap population and interatomic distances of Rb_3GaP_2.

Atom A	Atom B	r_{AB} / Å	overlap
Rb1	P2	3.412	0.018
	P4	3.476	0.03
	P1	3.521	0.013
	Ga1	3.559	−0.015
	P2	3.568	0.029
	Rb5	3.663	−0.004
	Rb3	3.693	−0.003
	Ga2	3.709	−0.004
	Rb6	3.978	−0.001
Rb2	P4	3.363	0.032
	P4	3.389	0.029
	P3	3.496	0.018
	P2	3.63	0.024
	Ga1	3.771	−0.011
	Rb6	3.895	−0.001
	Ga2	3.979	−0.002
Rb3	P1	3.491	0.016
	P3	3.52	0.016
	Ga1	3.536	−0.012
	Ga1	3.589	−0.011
	P2	3.594	0.028
	P2	3.642	0.022
	Rb5	3.942	−0.002
Rb4	P4	3.368	0.032
	P3	3.372	0.017
	Ga2	3.634	−0.01
	P4	3.641	0.022
	Ga1	3.664	−0.003
	Ga2	3.77	−0.001

Atom A	Atom B	r_{AB} / Å	overlap
Rb5	P2	3.468	0.033
	P3	3.571	0.014
	P2	3.603	0.037
	Ga2	3.662	−0.007
	P1	3.677	0.028
	Ga1	3.762	−0.006
	P1	3.854	0.015
Rb6	P4	3.58	0.031
	P1	3.59	0.018
	P3	3.652	0.028
	P2	3.734	0.032
	Ga2	3.842	−0.006
	P4	3.927	0.026
Ga1	P2	2.27	0.423
	P3	2.347	0.325
	P1	2.4	0.308
	Ga2	2.991	−0.087
Ga2	P4	2.243	0.46
	P3	2.384	0.318
	P1	2.392	0.299
P1	P3	3.687	−0.081
	P4	4.196	−0.029
	P2	4.239	−0.025
P2	P3	4.144	−0.029
P3	P4	4.18	−0.026

Table S39. Partial charges for each atom position in Rb_3GaP_2.

Atom	Z	charge	part charge
Rb1	9	8.3	0.7
Rb2		8.31	0.69
Rb3		8.288	0.712
Rb4		8.268	0.732
Rb5		8.312	0.688
Rb6		8.306	0.694
Ga1	31	31.246	−0.246
Ga2		31.192	−0.192
P1	15	15.848	−0.848
P2		16.086	−1.086
P3		15.798	−0.798
P4		16.047	−1.047

9 Structure type A

9.1 Cs_3AlP_2[15]

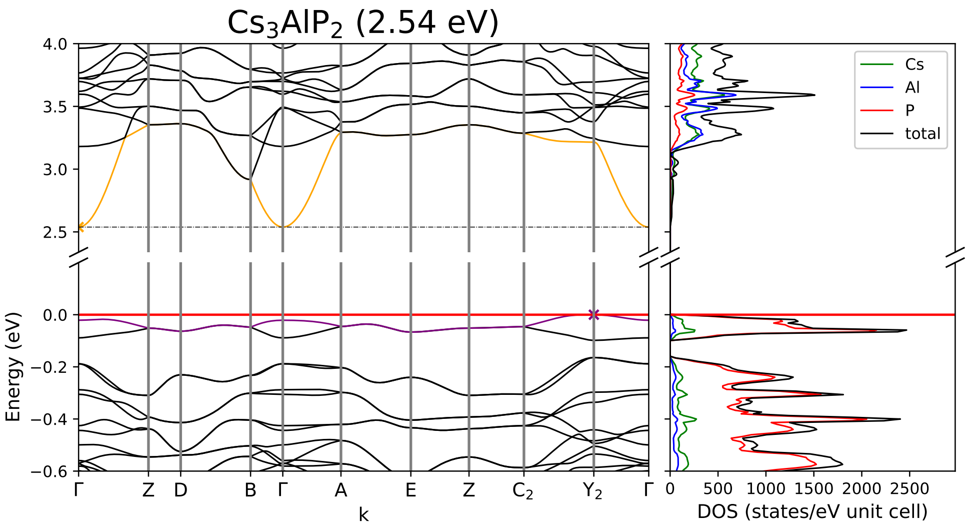

Figure S86. Band structure and DOS of Cs_3AlP_2.

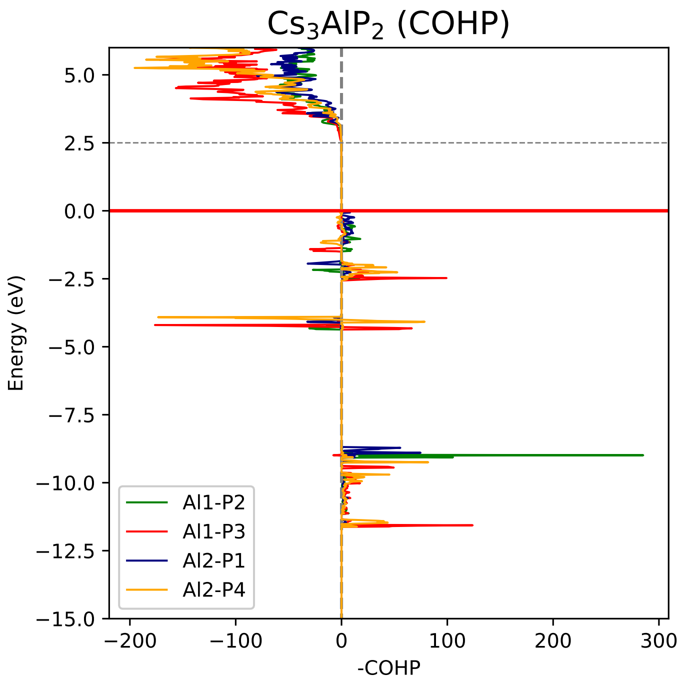

Figure S87. COHP of Al-P interactions in Cs_3AlP_2.

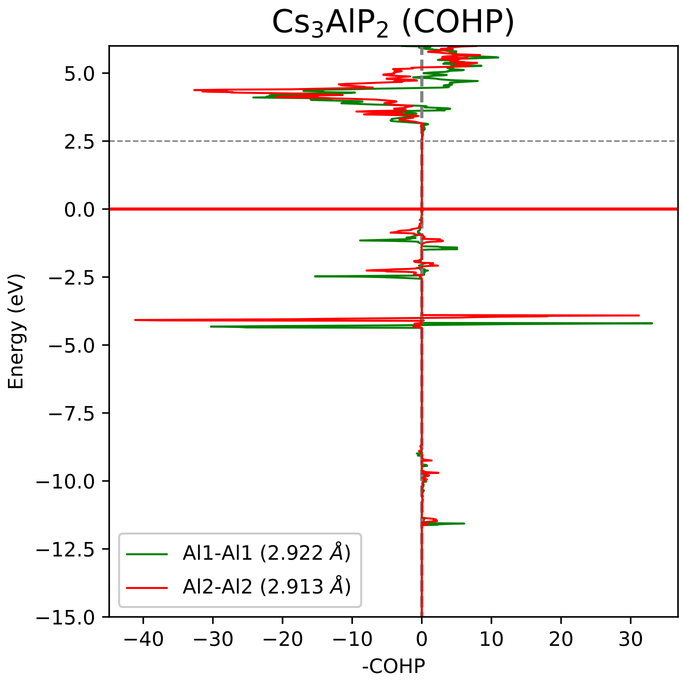

Figure S88. COHP of Al-Al interactions in Cs_3AlP_2.

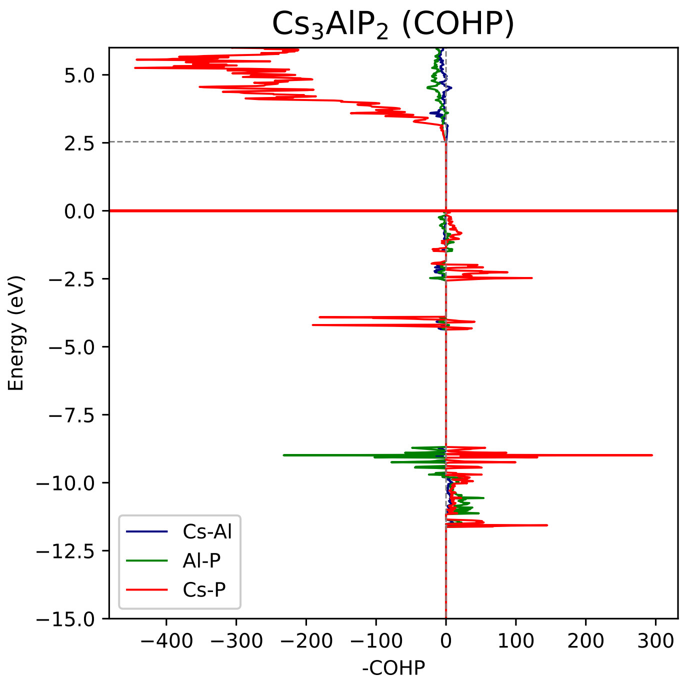

Figure S89. COHP of Cs-Al, Cs-P and Al-P interactions in Cs_3AlP_2.

Table S40. Overlap population and interatomic distances of Cs_3AlP_2.

Atom A	Atom B	r_{AB} / Å	overlap
Cs1	P4	3.511	0.016
	P1	3.555	0.034
	P1	3.647	0.029
	P2	3.659	0.032
	Al2	3.833	−0.003
Cs2	P2	3.49	0.022
	P3	3.491	0.012
	P1	3.662	0.033
	P2	3.734	0.024
	Al1	3.801	−0.006
	Cs4	3.801	0.006
	Cs5	3.927	0.007
Cs3	Al2	3.572	0.007
	P3	3.646	0.016
	P2	3.692	0.03
	P4	3.8	0.012
	Al1	3.804	−0.002
	Cs4	3.904	0.006
Cs4	P2	3.63	0.024
	Al2	3.729	0.004
	P1	3.774	0.022
	P3	3.797	0.014
	Al1	3.886	−0.001
	P4	3.942	0.011
	P3	3.994	0.019

Atom A	Atom B	r_{AB} / Å	overlap
Cs5	P4	3.54	0.021
	P1	3.564	0.029
	P2	3.669	0.028
	Al1	3.744	0.003
	P3	3.905	0.014
	P2	3.957	0.017
	Cs6	4.015	0.005
Cs6	P4	3.599	0.017
	P1	3.784	0.028
	Al2	3.831	0.001
	Al1	3.839	0.009
	Cs6	3.848	0.005
	P1	3.883	0.02
	P4	3.924	0.013
Al1	P2	2.272	0.471
	P3	2.36	0.34
	P3	2.373	0.327
	Al1	2.922	−0.04
Al2	P1	2.246	0.497
	P4	2.345	0.328
	P4	2.351	0.316
	Al2	2.913	−0.037
P1	P4	4.125	−0.03
	P4	4.145	−0.031
P2	P3	4.163	−0.028
	P3	4.179	−0.027
P3	P3	3.723	−0.08
P4	P4	3.683	−0.09

Table S41. Partial charges for each atom position in Cs_3AlP_2.

Atom	Z	charge	part charge
Cs1	9	8.318	0.682
Cs2		8.3	0.7
Cs3		8.271	0.729
Cs4		8.292	0.708
Cs5		8.294	0.706
Cs6		8.269	0.731
Al1	13	12.909	0.091
Atom	Z	charge	partialcharge
Al2		12.926	0.074
P1	15	16.213	−1.213
P2		16.233	−1.233
P3		15.989	−0.989
P4		15.985	−0.985

9.2 Cs_3GaP_2[16]

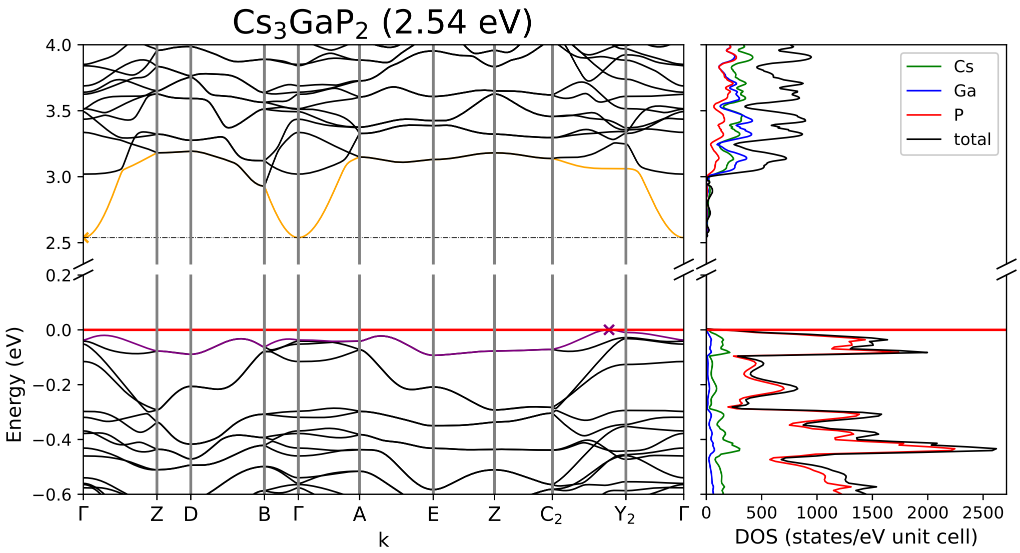

Figure S90. Band structure and DOS of Cs_3GaP_2.

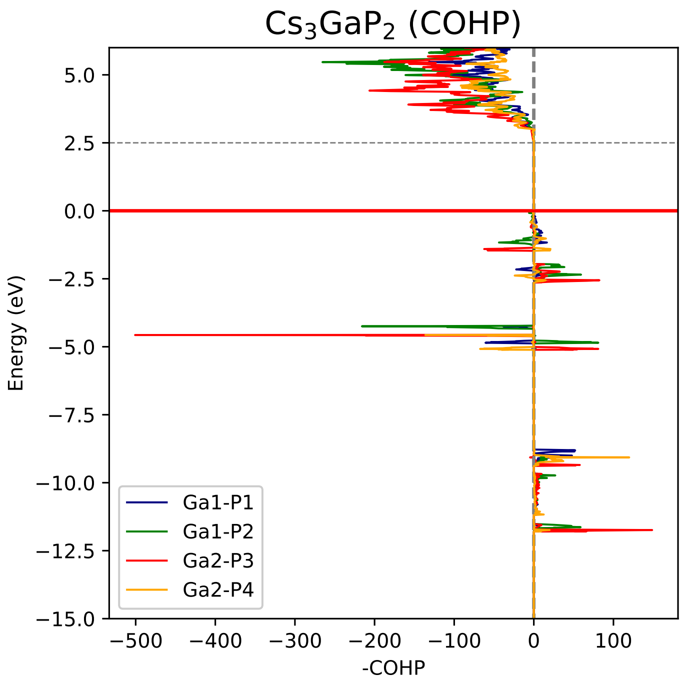

Figure S91. COHP of Ga-P interactions in Cs_3GaP_2.

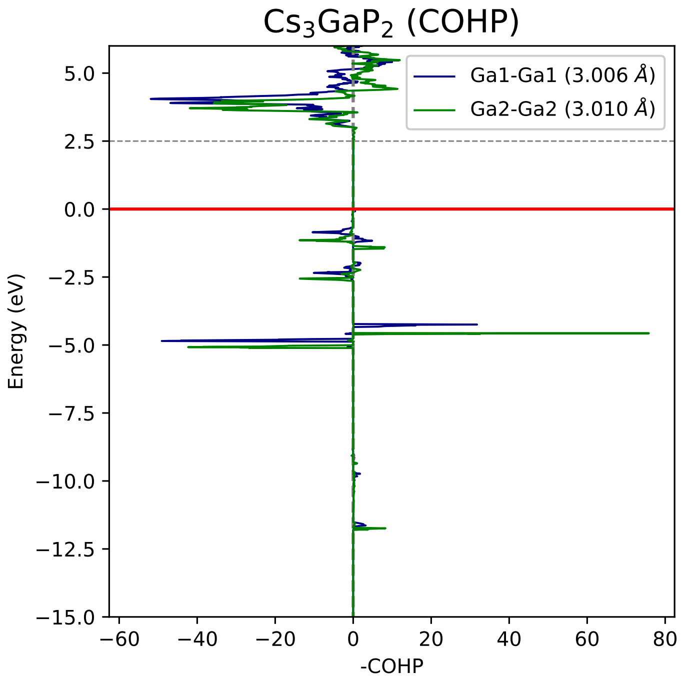

Figure S92. COHP of Ga-Ga interactions in Cs_3GaP_2.

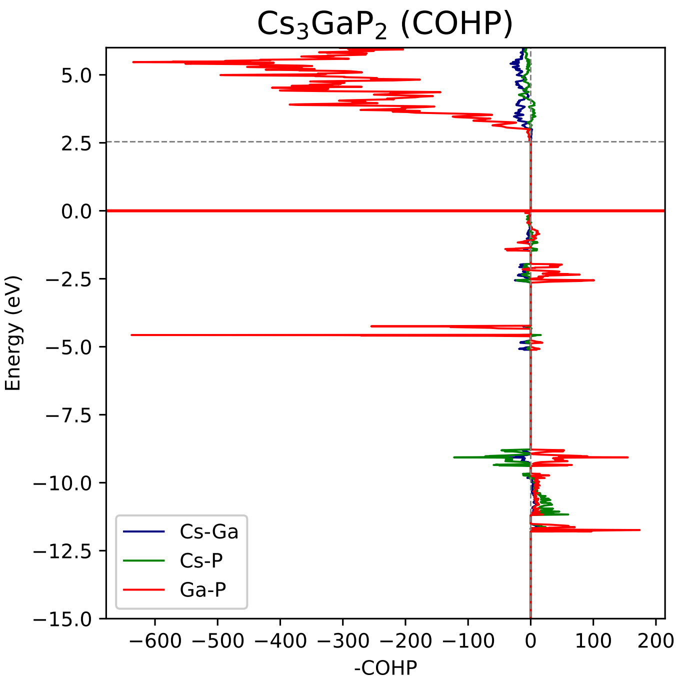

Figure S93. COHP of Cs-Ga, Cs-P and Ga-P interactions in Cs_3GaP_2.

Table S42. Overlap population and interatomic distances of Cs_3GaP_2.

Atom A	Atom B	r_{AB} / Å	overlap
Cs1	P4	3.625	0.023
	Ga1	3.71	−0.003
	P1	3.764	0.02
	P3	3.769	0.014
	Cs6	3.814	0.006
	Ga2	3.859	−0.008
	Cs4	3.891	0.006
	P2	3.967	0.011
Cs2	P1	3.527	0.029
	P2	3.556	0.024
	P4	3.637	0.027
	Ga2	3.711	−0.003
	P3	3.893	0.015
	Cs6	3.959	0.007
	P4	3.964	0.015
Cs3	P2	3.579	0.019
	P1	3.803	0.026
	Ga1	3.809	−0.005
	Ga2	3.826	0.004
	Cs3	3.851	0.005
	P1	3.889	0.018
	P2	3.932	0.013
Cs4	Ga1	3.569	0.0
	P3	3.642	0.016
	P4	3.716	0.029
	P2	3.768	0.013
	Ga2	3.782	−0.009

Atom A	Atom B	r_{AB} / Å	overlap
Cs5	P2	3.493	0.018
	P1	3.547	0.034
	P4	3.644	0.033
	P1	3.648	0.028
	Ga1	3.787	−0.011
Cs6	P3	3.473	0.012
	P4	3.491	0.021
	P1	3.65	0.034
	P4	3.727	0.025
	Ga2	3.754	−0.014
Ga1	P1	2.251	0.463
	P2	2.363	0.313
	P2	2.366	0.301
	Ga1	3.006	−0.08
Ga2	P4	2.277	0.423
	P3	2.375	0.328
	P3	2.39	0.315
	Ga2	3.01	−0.082
P1	P2	4.159	−0.03
	P2	4.187	−0.031
P2	P2	3.651	−0.08
P3	P3	3.694	−0.071

Table S43. Partial charges for each atom position in Cs_3GaP_2.

Atom	Z	charge	part charge
Cs1	9	8.281	0.719
Cs2		8.291	0.709
Cs3		8.25	0.75
Cs4		8.256	0.744
Cs5		8.315	0.685
Cs6		8.298	0.702
Ga1	31	31.203	−0.203

Atom	Z	charge	partialcharge
Ga2		31.197	−0.197
P1	15	16.109	−1.109
P2		15.837	−0.837
P3		15.836	−0.836
P4		16.126	−1.126

9.3 Cs_3AlAs_2[17]

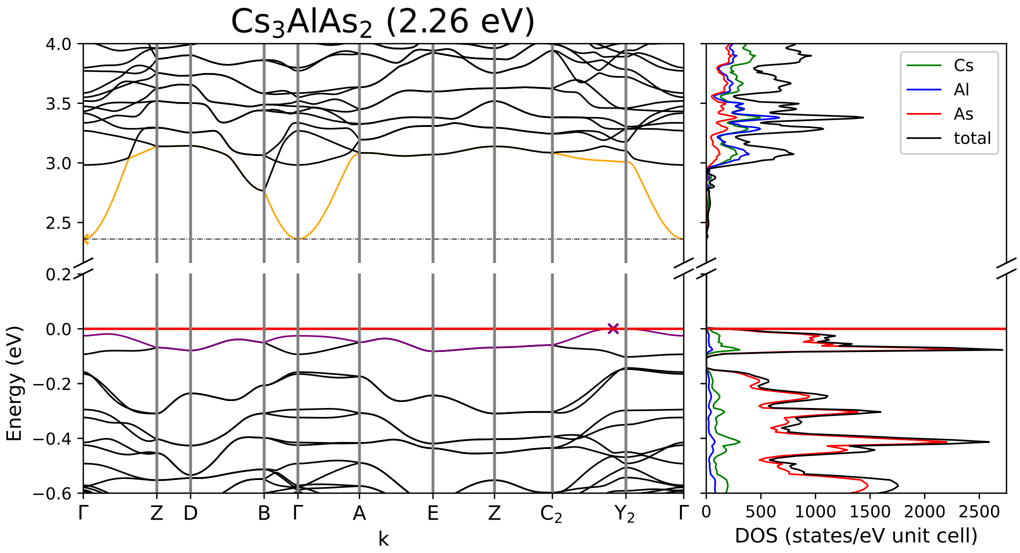

Figure S94. Band structure and DOS of Cs_3AlAs_2.

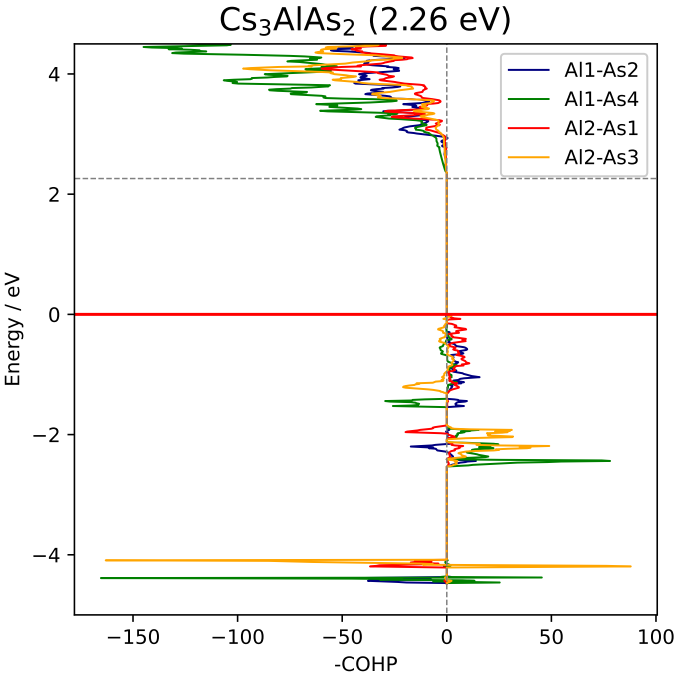

Figure S95. COHP of Al-As interactions in Cs_3AlAs_2.

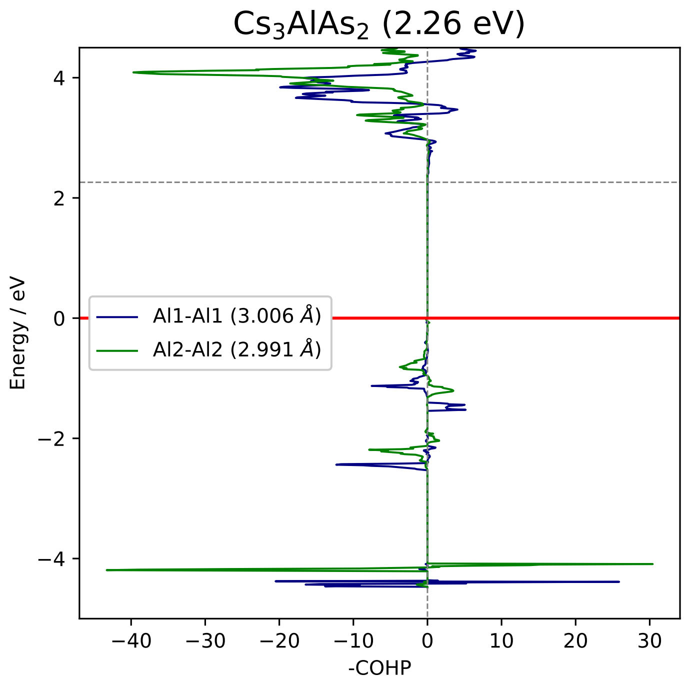

Figure S96. COHP of Al-Al interactions in Cs_3AlAs_2.

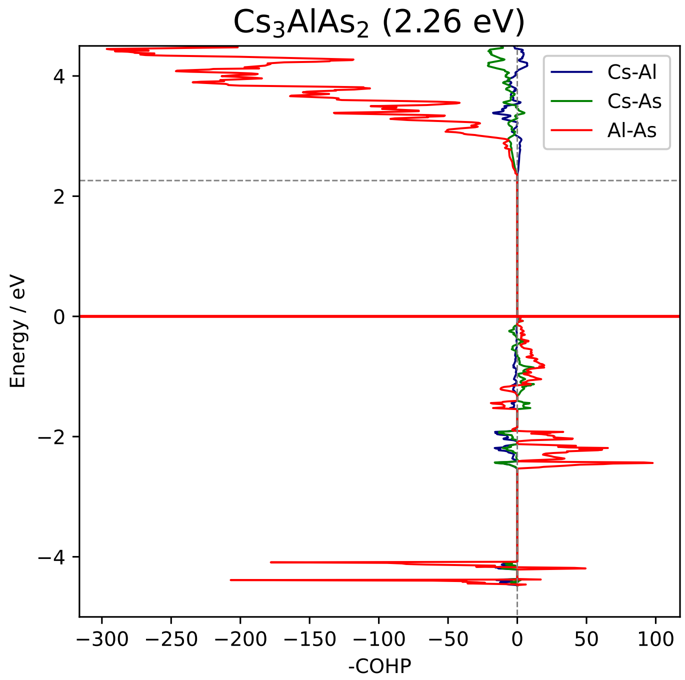

Figure S97. COHP of Cs-Al, Cs-As and Al-As interactions in Cs_3AlAs_2.

Table S44. Partial charges for each atom position in Cs_3AlAs_2.

Atom	Z	charge	part charge
Cs1	9	8.339	0.661
Cs2		8.323	0.677
Cs3		8.294	0.706
Cs4		8.318	0.682
Cs5		8.316	0.684
Cs6		8.29	0.71
Al1	13	12.782	0.218

Atom	Z	charge	partialcharge
Al2		12.789	0.211
As1	33	34.223	−1.223
As2		34.237	−1.237
As3		34.045	−1.045
As4		34.042	−1.042

Table S45. Overlap population and interatomic distances of Cs_3AlAs_2.

Atom A	Atom B	r_{AB} / Å	overlap
Cs1	As3	3.58	0.02
	As1	3.598	0.04
	As2	3.71	0.037
	As1	3.717	0.035
	Al2	3.904	0.0
Cs2	As4	3.563	0.015
	As2	3.571	0.03
	As1	3.722	0.038
	As2	3.769	0.029
	Cs4	3.869	0.007
	Al1	3.888	−0.002
Cs3	Al2	3.613	0.012
	As4	3.75	0.018
	As2	3.777	0.035
	As3	3.854	0.016
	Al1	3.885	0.002
Cs4	As2	3.721	0.029
	Al2	3.782	0.007
	As4	3.839	0.016
	As1	3.842	0.026
	Al1	3.964	0.002

Atom A	Atom B	r_{AB} / Å	overlap
Cs5	As1	3.606	0.034
	As3	3.614	0.025
	As2	3.701	0.03
	Al1	3.761	0.006
	As4	3.965	0.017
Cs6	As3	3.669	0.02
	Al1	3.875	0.011
	As1	3.883	0.031
	Al2	3.89	0.004
	Cs6	3.91	0.005
	As1	3.94	0.023
Al1	As2	2.362	0.451
	As4	2.453	0.332
	As4	2.468	0.319
	Al1	3.006	−0.027
Al2	As1	2.334	0.475
	As3	2.437	0.322
	As3	2.442	0.312
	Al2	2.991	−0.029
As4	As4	3.897	−0.064

9.4 Cs_3GaAs_2[18]

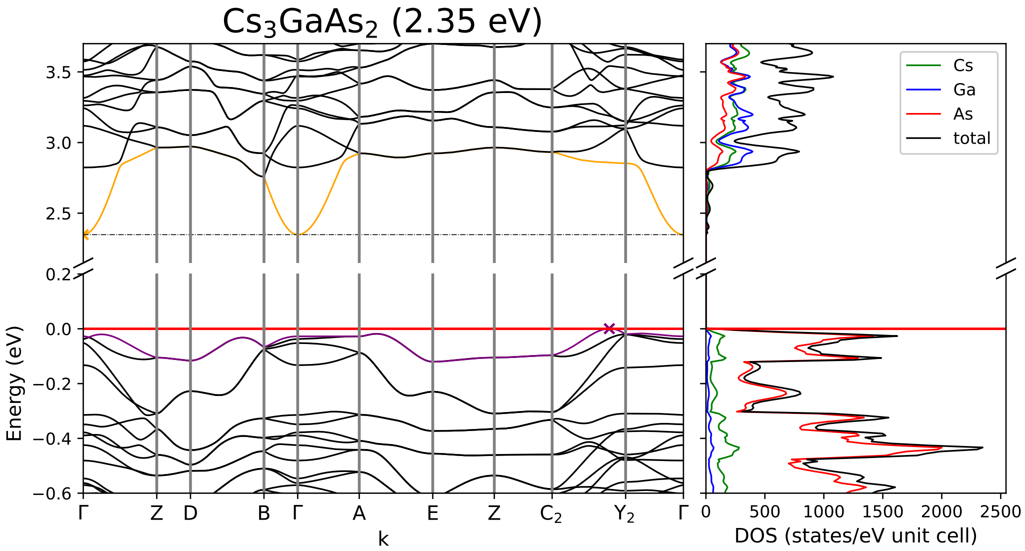

Figure S98. Band structure and DOS of Cs_3GaAs_2.

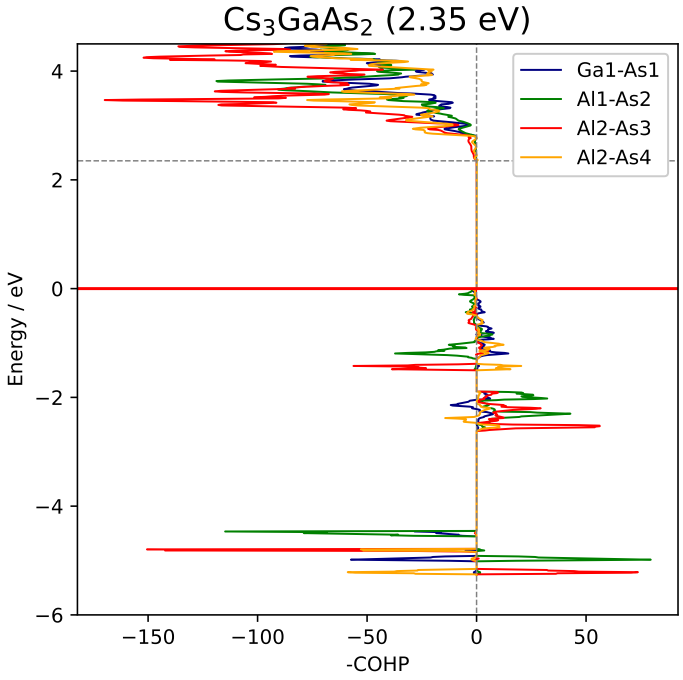

Figure S99. COHP of Ga-As interactions in Cs_3GaAs_2.

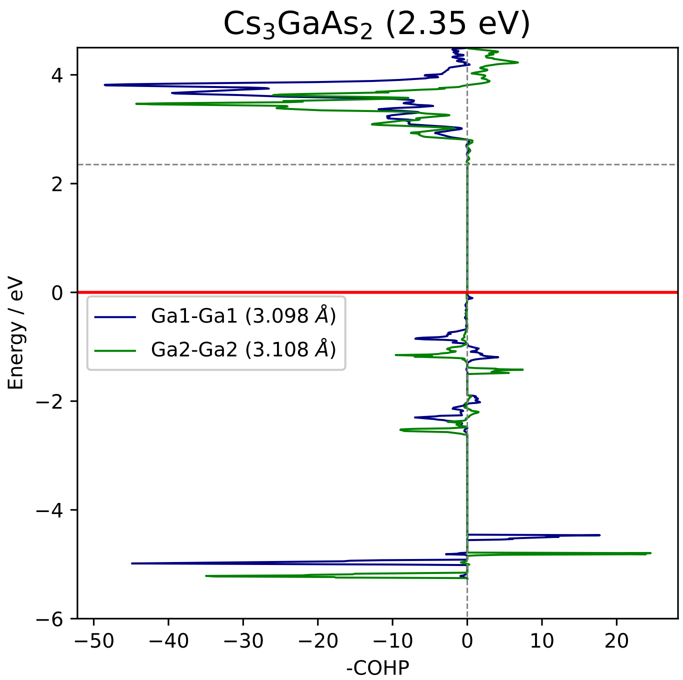

Figure S100. COHP of Ga-Ga interactions in Cs_3GaAs_2.

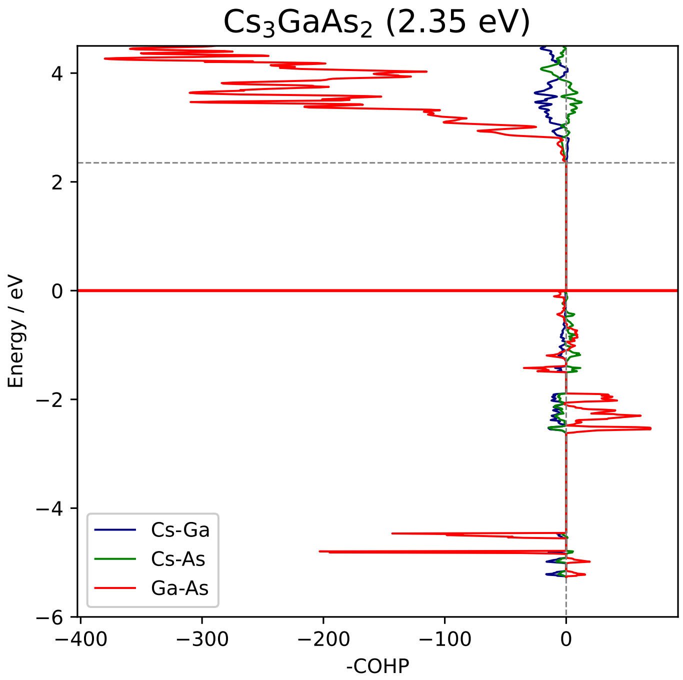

Figure S101. COHP of Cs-Ga, Cs-As and Ga-As interactions in Cs_3GaAs_2.

Table S46. Partial charges for each atom position in Cs_3GaAs_2.

Atom	Z	charge	part charge
Cs1	9	8.306	0.694
Cs2		8.311	0.689
Cs3		8.271	0.729
Cs4		8.278	0.722
Cs5		8.334	0.666
Cs6		8.32	0.68
Ga1	31	31.06	−0.06

Atom	Z	charge	partialcharge
Ga2		31.056	−0.056
As1	33	34.129	−1.129
As2		33.899	−0.899
As3		33.898	−0.898
As4		34.137	−1.137

Table S47. Overlap population and interatomic distances of Cs_3GaAs_2.

Atom A	Atom B	r_{AB} / Å	overlap
Cs1	As4	3.717	0.028
	Ga1	3.758	0.001
	As3	3.819	0.016
	As1	3.836	0.024
	Cs6	3.893	0.007
	Ga2	3.934	−0.003
Cs2	As1	3.57	0.033
	As2	3.638	0.028
	As4	3.666	0.029
	Ga2	3.73	−0.001
	As3	3.944	0.017
	As4	4.009	0.018
Cs3	As2	3.65	0.02
	Ga2	3.86	0.007
	Ga1	3.866	−0.001
	As1	3.903	0.029
	Cs3	3.915	0.006
	As1	3.935	0.022
Cs4	Ga1	3.607	0.004
	As3	3.744	0.018
	As4	3.81	0.034
	As2	3.833	0.017
	Ga2	3.865	−0.003

Atom A	Atom B	r_{AB} / Å	overlap
Cs5	As2	3.557	0.02
	As1	3.589	0.039
	As4	3.699	0.037
	As1	3.721	0.032
	Ga1	3.852	−0.005
Cs6	As3	3.544	0.014
	As4	3.572	0.028
	As1	3.72	0.04
	As4	3.767	0.03
	Ga2	3.832	−0.007
Ga1	As1	2.335	0.43
	As2	2.452	0.295
	As2	2.455	0.305
	Ga1	3.098	−0.067
Ga2	As4	2.364	0.397
	As3	2.465	0.322
	As3	2.484	0.305
	Ga2	3.108	−0.065
As2	As2	3.805	−0.07
As3	As3	3.851	−0.063

10 Structure predictions

10.1 K_3GaP_2

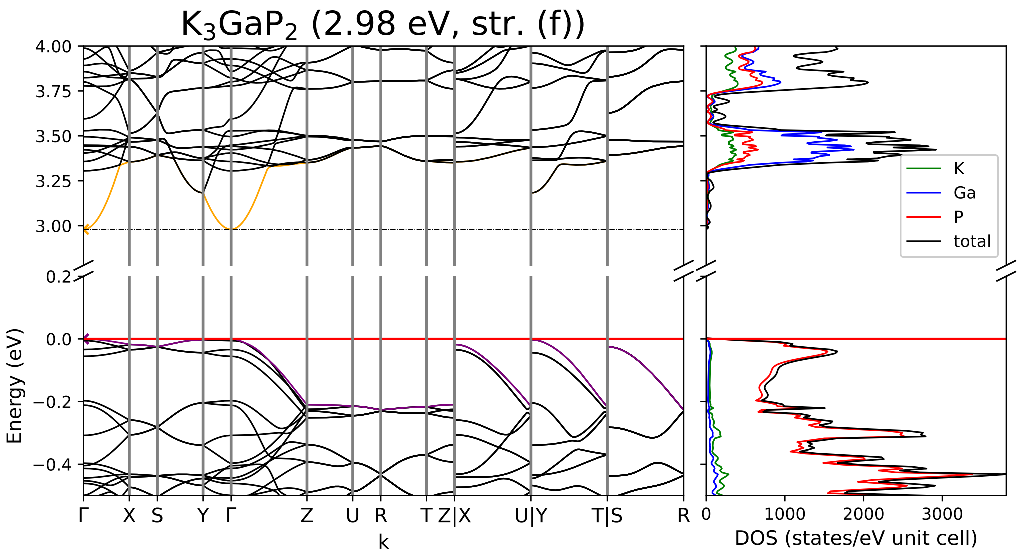

Figure S102. Band structure and DOS of K_3GaP_2 within structure-type **B**.

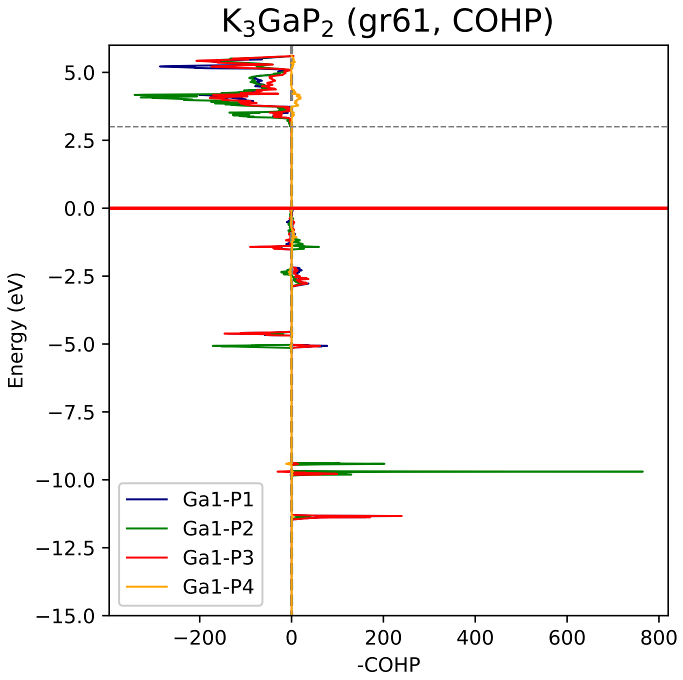

Figure S103. COHP of Ga1-P interactions in K_3GaP_2.

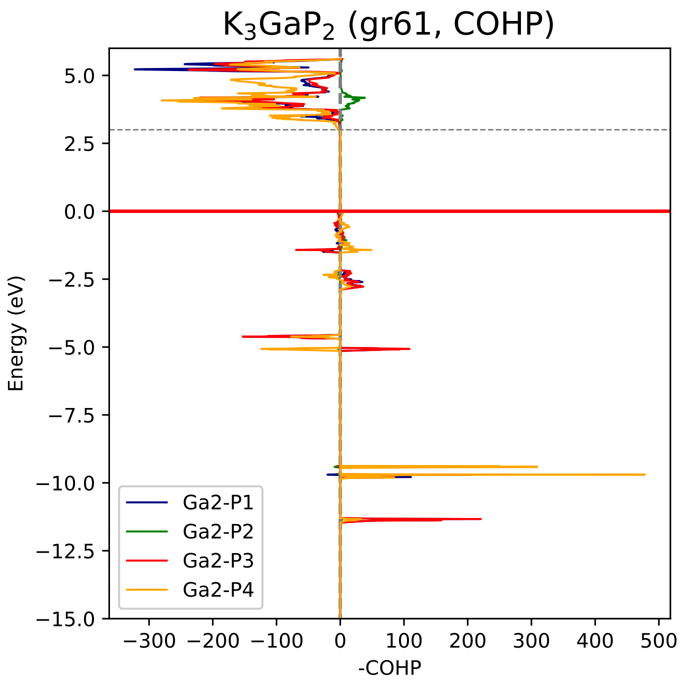

Figure S104. COHP of Ga2-P interactions in K_3GaP_2.

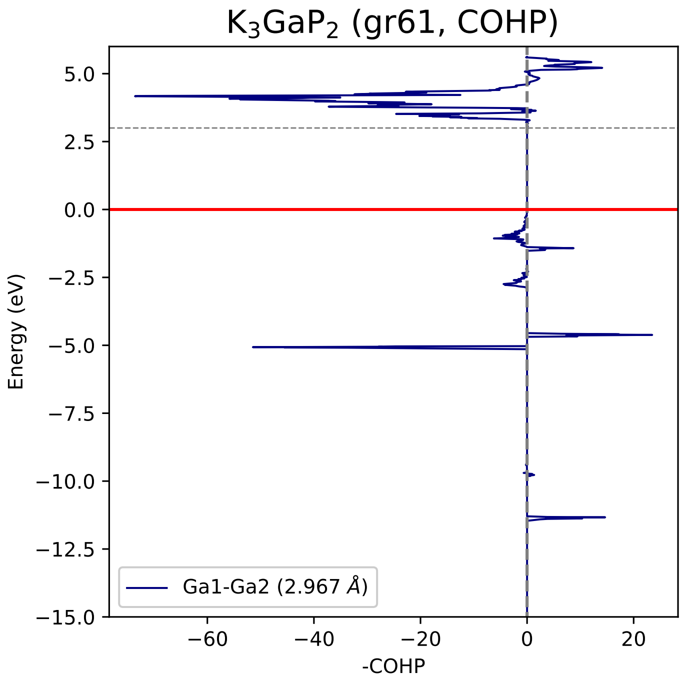

Figure S105. COHP of Ga-Ga interactions in K_3GaP_2.

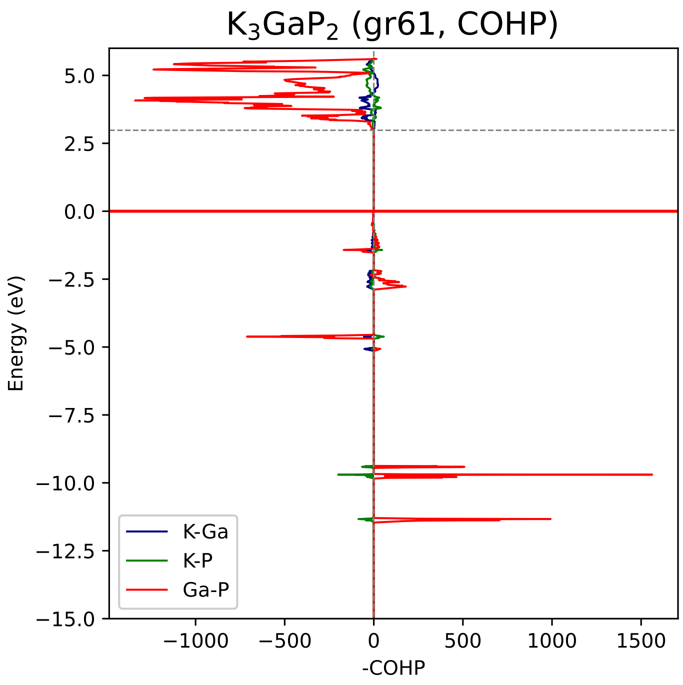

Figure S106. COHP of K-Ga, K-P and Ga-P interactions in K_3GaP_2.

Table S48. Partial charges for each atom position in K_3GaP_2.

Atom	Z	charge	part charge
K1	19	18.24	0.76
K2		18.237	0.763
K3		18.227	0.773
K4		18.207	0.793
K5		18.248	0.752
K6		18.246	0.754
Ga1	31	31.323	−0.323

Atom	Z	charge	partialcharge
Ga2		31.245	−0.245
P1	15	15.875	−0.875
P2		16.171	−1.171
P3		15.841	−0.841
P4		16.14	−1.14

Table S49. Overlap population and interatomic distances of K_3GaP_2.

Atom A	Atom B	r_{AB} / Å	overlap
K1	P2	3.254	0.012
	P4	3.291	0.024
	P2	3.337	0.028
	P1	3.392	0.011
	Ga1	3.396	−0.018
	K5	3.489	−0.001
	K3	3.528	0.001
	Ga2	3.562	−0.005
	K6	3.773	0.001
	K4	3.86	0.001
	K3	3.883	0.001
	P1	3.944	0.007
	K5	3.945	0.001
K2	P4	3.204	0.02
	P4	3.205	0.026
	P3	3.354	0.014
	P2	3.455	0.02
	Ga1	3.586	−0.01
	K6	3.696	0.001
	Ga2	3.816	−0.003
	K3	3.866	0.001
	K4	3.975	0.001
K3	P3	3.359	0.016
	P1	3.37	0.012
	Ga1	3.376	−0.014
	Ga1	3.432	−0.013
	P2	3.481	0.022
	P2	3.521	0.02
	P1	3.827	0.021
	K5	3.852	0.001
K4	P4	3.182	0.027
	P3	3.217	0.012
	Ga2	3.454	−0.01
	Ga1	3.47	−0.004
	P4	3.52	0.017
	Ga2	3.639	−0.003
	P3	3.841	0.006
	P1	3.861	0.007
	K4	3.955	0.0
	K6	3.962	0.002

Atom A	Atom B	r_{AB} / Å	overlap
K5	P2	3.283	0.027
	P2	3.427	0.034
	P3	3.435	0.01
	P1	3.452	0.026
	Ga2	3.465	−0.008
	Ga1	3.604	−0.007
	P1	3.715	0.013
K6	P1	3.456	0.015
	P4	3.469	0.026
	P3	3.469	0.025
	P2	3.546	0.028
	Ga2	3.681	−0.006
	P4	3.69	0.026
Ga1	P2	2.258	0.426
	P3	2.341	0.318
	P1	2.391	0.306
	Ga2	2.967	−0.088
	P1	4.917	0.003
Ga2	P4	2.234	0.469
	P3	2.382	0.313
	P1	2.385	0.293
P1	P3	3.697	−0.084
	P4	4.182	−0.032
	P2	4.215	−0.029
P2	P3	4.119	−0.033
	P3	4.923	0.0
P3	P4	4.161	−0.03
	P4	4.994	0.0
P4	P4	4.862	−0.002

10.2 Rb_3AlP_2

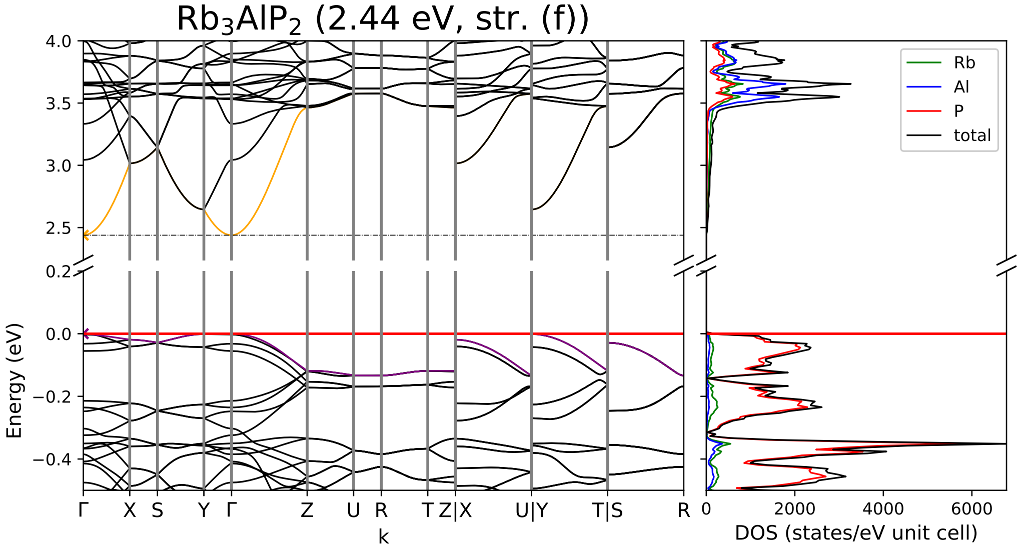

Figure S107. Band structure and DOS of Rb_3AlP_2 within structure-type **B**.

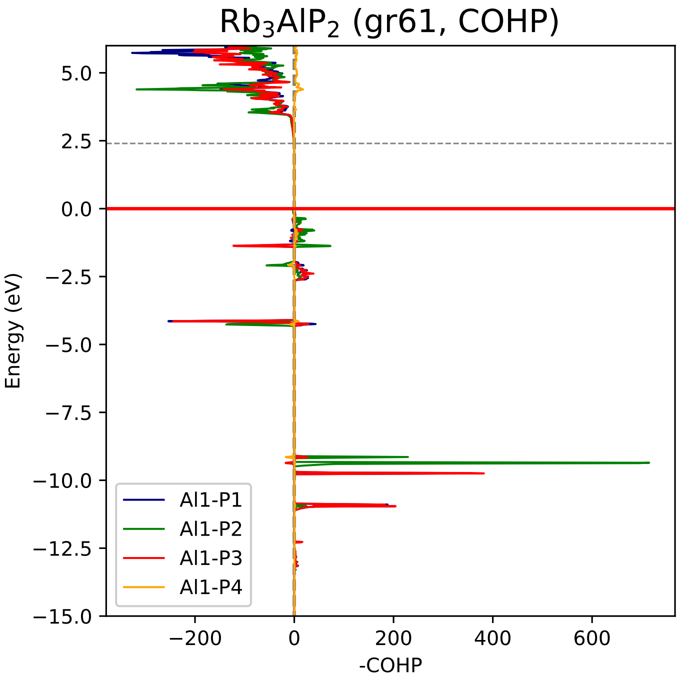

Figure S108. COHP of Al1-P interactions in Rb_3AlP_2.

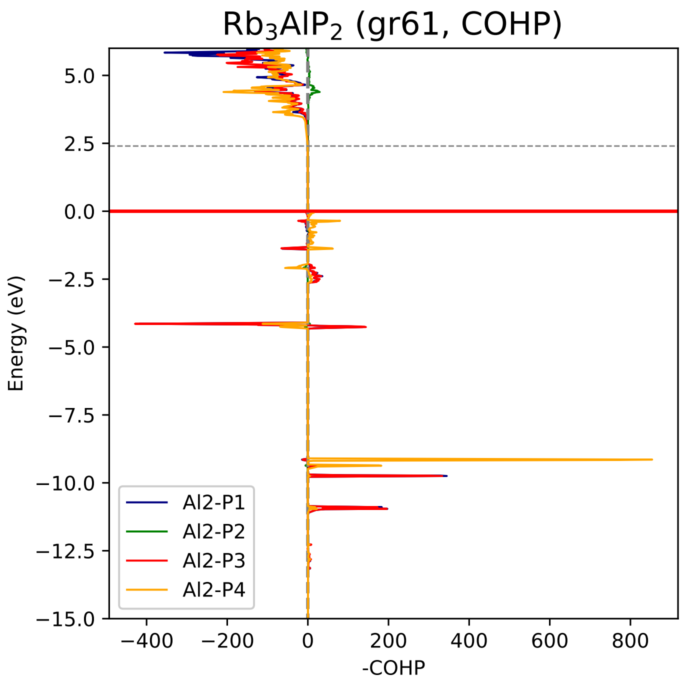

Figure S109. COHP of Al2-P interactions in Rb_3AlP_2.

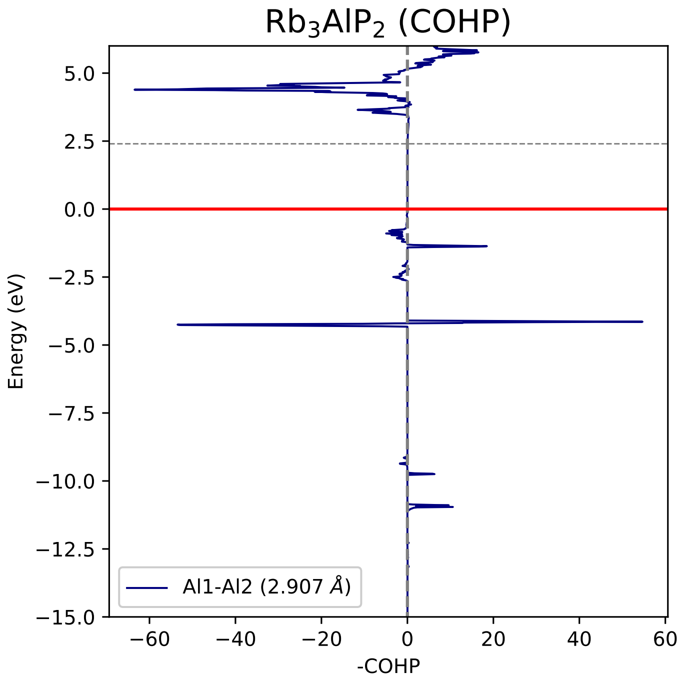

Figure S110. COHP of Al-Al interactions in Rb_3AlP_2.

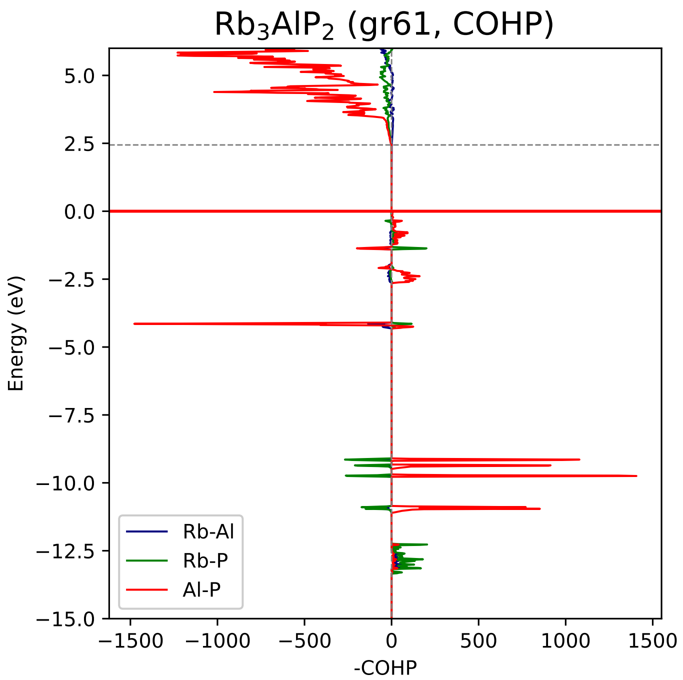

Figure S111. COHP of Rb-Al, Rb-P and Al-P interactions in Rb_3AlP_2.

Table S50. Partial charges for each atom position in Rb_3AlP_2.

Atom	Z	charge	part charge
Rb1	9	8.312	0.688
Rb2		8.315	0.685
Rb3		8.299	0.701
Rb4		8.283	0.717
Rb5		8.315	0.685
Rb6		8.306	0.694
Al1	13	12.964	0.036

Atom	Z	charge	partialcharge
Al2		12.928	0.072
P1	15	15.992	−0.992
P2		16.188	−1.188
P3		15.946	−0.946
P4		16.151	−1.151

Table S51. Overlap population and interatomic distances of Rb_3AlP_2.

Atom A	Atom B	r_{AB} / Å	overlap
Rb1	P2	3.422	0.02
	P4	3.482	0.032
	P1	3.518	0.012
	P2	3.588	0.027
	Al1	3.594	−0.007
	Rb5	3.64	−0.005
	Rb3	3.68	−0.004
	Al2	3.719	0.003
	Rb6	3.986	0.0
Rb2	P4	3.372	0.031
	P4	3.389	0.031
	P3	3.516	0.017
	P2	3.642	0.025
	Al1	3.797	−0.003
	Rb6	3.874	−0.002
	Al2	3.975	0.003
Rb3	P1	3.498	0.016
	P3	3.506	0.016
	Al1	3.557	−0.004
	P2	3.594	0.028
	Al1	3.605	−0.002
	P2	3.614	0.024
	Rb5	3.94	−0.002
Rb4	P3	3.37	0.015
	P4	3.371	0.033
	P4	3.632	0.025
	Al2	3.654	−0.003
	Al1	3.697	0.002
	Al2	3.782	0.003
Rb5	P2	3.478	0.034
	P3	3.594	0.016
	P2	3.599	0.037
	P1	3.662	0.026
	Al2	3.693	−0.002
	Al1	3.778	−0.002
	P1	3.904	0.015
Rb6	P4	3.581	0.033
	P1	3.583	0.018
	P3	3.649	0.026
	P2	3.753	0.032
	Al2	3.867	0.0
	P4	3.941	0.025
Al1	P2	2.263	0.471
	P3	2.333	0.34
	P1	2.38	0.319
	Al2	2.907	−0.044
Al2	P4	2.237	0.504
	P3	2.366	0.323
	P1	2.374	0.31
P1	P3	3.71	−0.088
	P4	4.158	−0.029
	P2	4.193	−0.026
P2	P3	4.116	−0.03
P3	P4	4.142	−0.027

10.3 Na_3GaAs_2

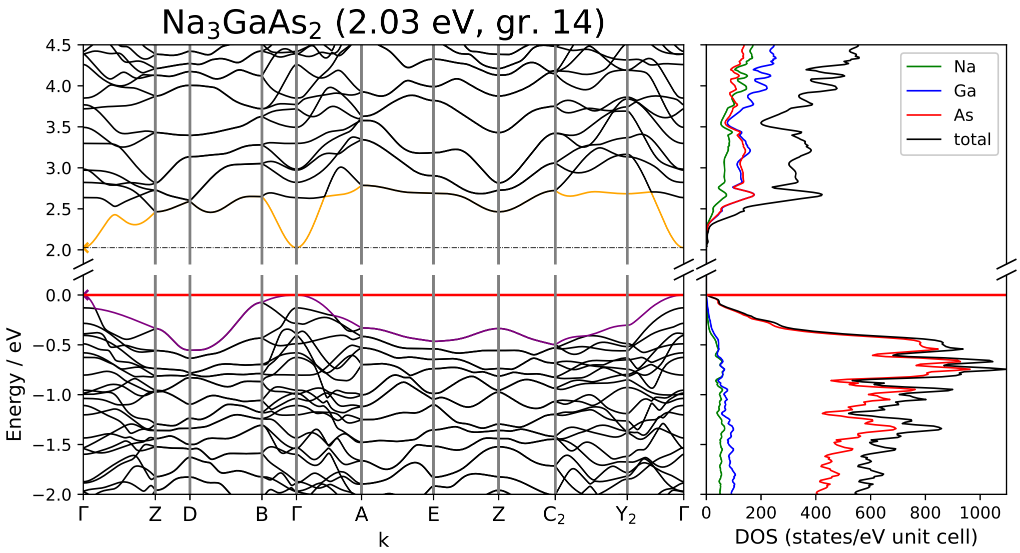

Figure S112. Band structure and DOS of Na_3GaAs_2 within structure-type **H**.

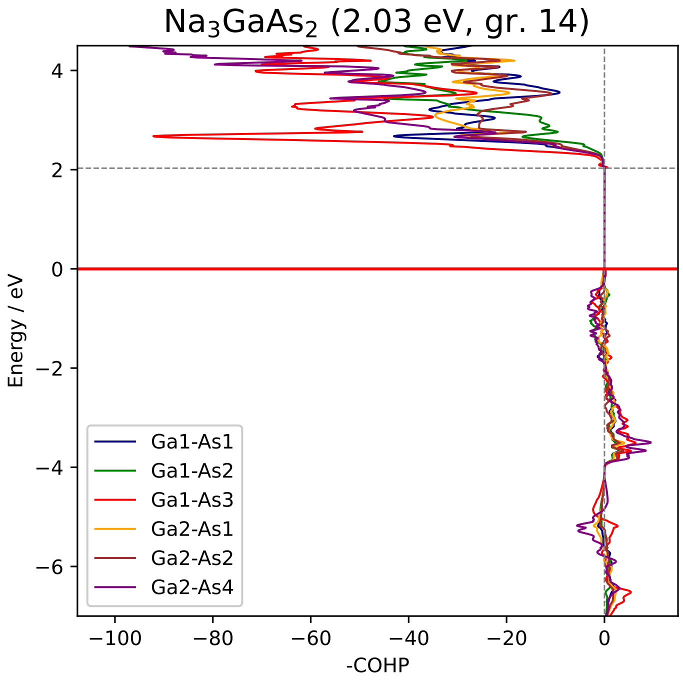

Figure S113. COHP of Ga-As interactions in Na_3GaAs_2.

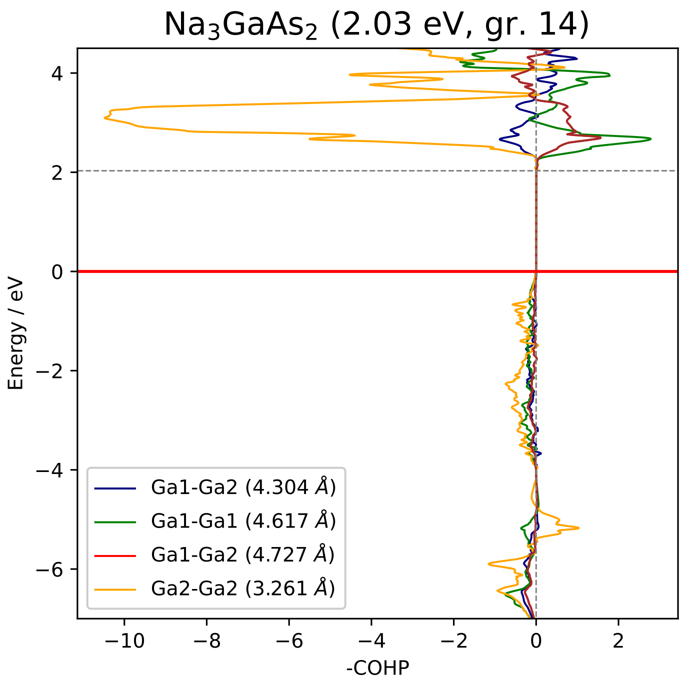

Figure S114. COHP of Ga-Ga interactions in Na_3GaAs_2.

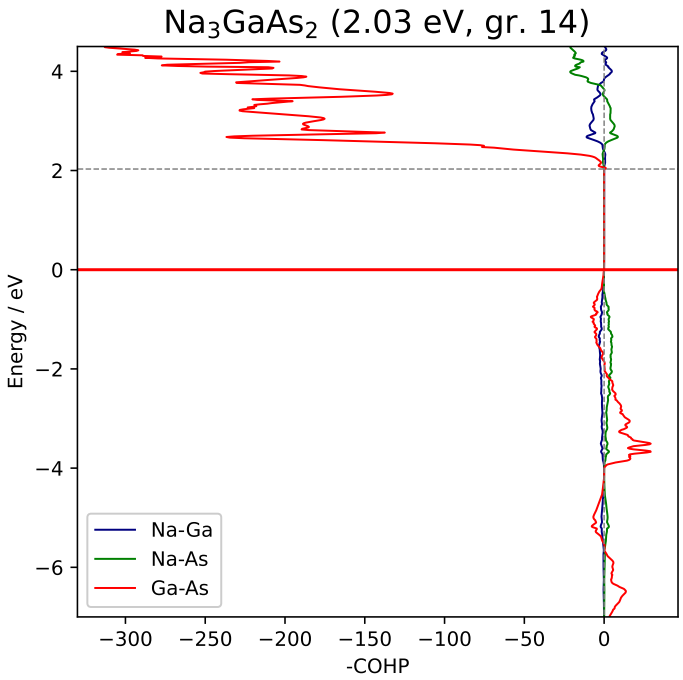

Figure S115. COHP of Na-Ga, Na-As and Ga-As interactions in Na_3GaAs_2.

Table S52. Partial charges for each atom position in Na_3GaAs_2.

Atom	Z	charge	part charge
Na1	11	10.306	0.694
Na2		10.261	0.739
Na3		10.274	0.726
Na4		10.344	0.656
Na5		10.262	0.738
Na6		10.269	0.731
Ga1	31	31.141	−0.141

Atom	Z	charge	partialcharge
Ga2		31.02	−0.02
As1	33	34.031	−1.031
As2		33.998	−0.998
As3		34.015	−1.015
As4		34.078	−1.078

10.4 K_3GaAs_2

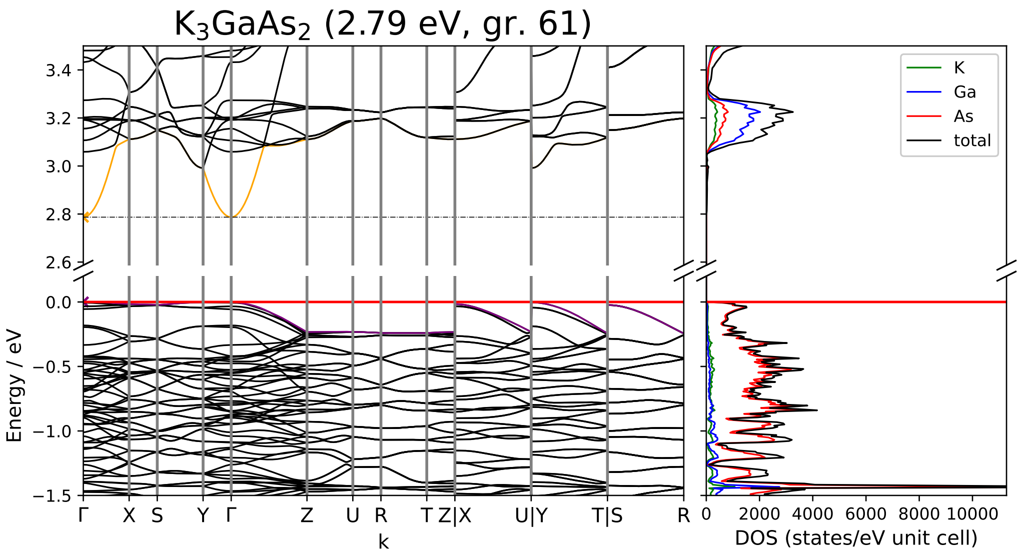

Figure S116. Band structure and DOS of K_3GaAs_2 within structure-type **B**.

Table S53. Overlap population and interatomic distances of Na_3GaAs_2.

Atom A	Atom B	r_{AB} / Å	overlap
Na1	As4	2.811	0.048
	As2	2.858	0.028
	As3	2.86	0.051
	As2	2.933	0.056
	Na3	3.202	0.007
	Na5	3.217	0.005
	Na3	3.244	0.004
	Na5	3.26	0.006
	Ga2	3.298	0.0
	Na6	3.337	0.006
	Ga1	3.412	−0.001
	Na2	3.502	0.002
Na2	As3	2.94	0.026
	As1	2.975	0.052
	As1	3.087	0.035
	Ga1	3.11	0.003
	Ga2	3.166	0.003
	Na4	3.19	0.007
	As3	3.192	0.031
	Na4	3.407	0.004
	Na2	3.459	0.005
	Ga1	3.478	0.003
	As4	3.48	0.014
	Na3	3.59	0.002
	As2	3.661	0.001
	Na6	3.804	0.002
	Na6	3.827	0.001
Na3	As4	3.012	0.032
	As1	3.012	0.02
	As2	3.124	0.034
	As3	3.145	0.051
	Ga2	3.21	0.003
	Na4	3.319	0.005
	Ga1	3.327	0.005
	As2	3.363	0.028
	Ga2	3.511	0.001
	Na6	3.546	0.003
	Na5	3.827	0.002
	Na5	3.916	0.0
	As4	3.951	0.003
Na4	As3	2.802	0.048
	As4	2.852	0.054
	As1	2.902	0.047
	As1	2.92	0.061
	Na6	3.046	0.007
	Na6	3.271	0.004
	Na5	3.301	0.004
	Na4	3.398	0.004
	Ga1	3.419	−0.001
Na5	As4	2.972	0.04
	As4	3.032	0.036
	As1	3.091	0.019
	As2	3.128	0.036
	Ga2	3.349	0.007
	As2	3.367	0.025
	Ga1	3.37	0.004
	Ga2	3.54	0.002
	Na5	3.565	0.001
	Na6	3.702	0.002
Na6	As2	2.857	0.031
	As3	3.103	0.022
	As3	3.112	0.037
	As1	3.272	0.03
	Ga1	3.275	0.004
	As4	3.308	0.035
	Ga1	3.401	0.003
	Ga2	3.531	0.005
	As1	3.83	0.006
Ga1	As2	2.536	0.279
	As3	2.538	0.274
	As1	2.609	0.271
	As3	2.656	0.252
	Ga2	4.304	−0.021
	Ga1	4.617	−0.017
	As3	4.719	0.004
	Ga2	4.727	−0.013
Ga2	As4	2.495	0.272
	As4	2.54	0.25
	As2	2.574	0.272
	As1	2.598	0.269
	Ga2	3.261	−0.052
	As4	4.779	0.005
As1	As4	4.152	−0.03
	As3	4.233	−0.022
	As2	4.237	−0.022
	As3	4.278	−0.02
	As2	4.314	−0.017
	As4	4.361	−0.023
	As4	4.508	−0.005
	As3	4.669	−0.004
	As1	4.727	−0.004
As2	As4	4.042	−0.024
	As3	4.214	−0.024
	As4	4.25	−0.029
	As3	4.27	−0.025
	As2	4.67	−0.004
	As4	4.89	−0.003
	As4	4.934	−0.002
As3	As3	4.067	−0.024
	As4	4.599	−0.002
	As4	4.777	−0.003
As4	As4	3.837	−0.056
	As4	4.831	−0.001

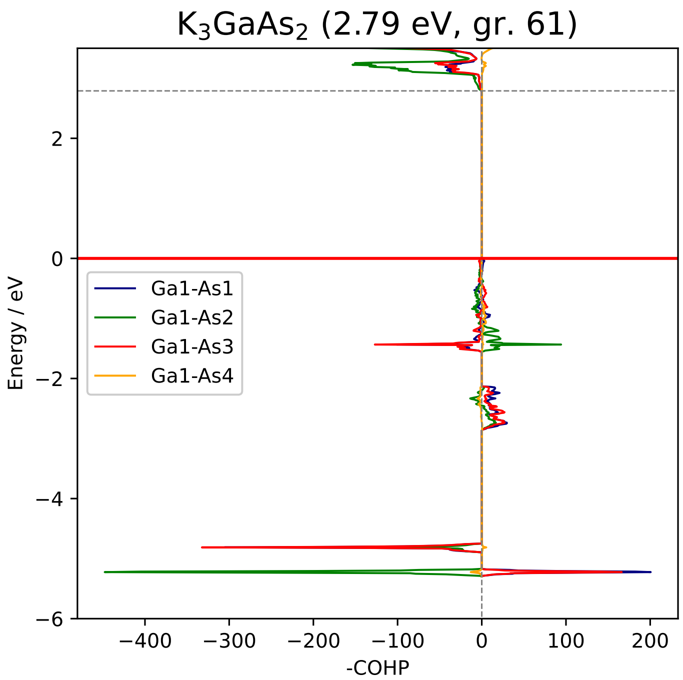

Figure S117. COHP of Ga1-As interactions in K_3GaAs_2.

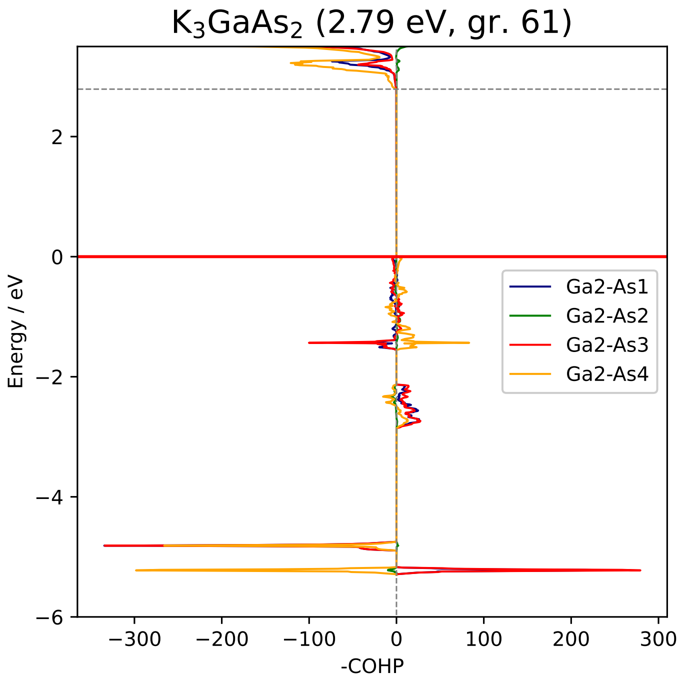

Figure S118. COHP of Ga2-As interactions in K_3GaAs_2.

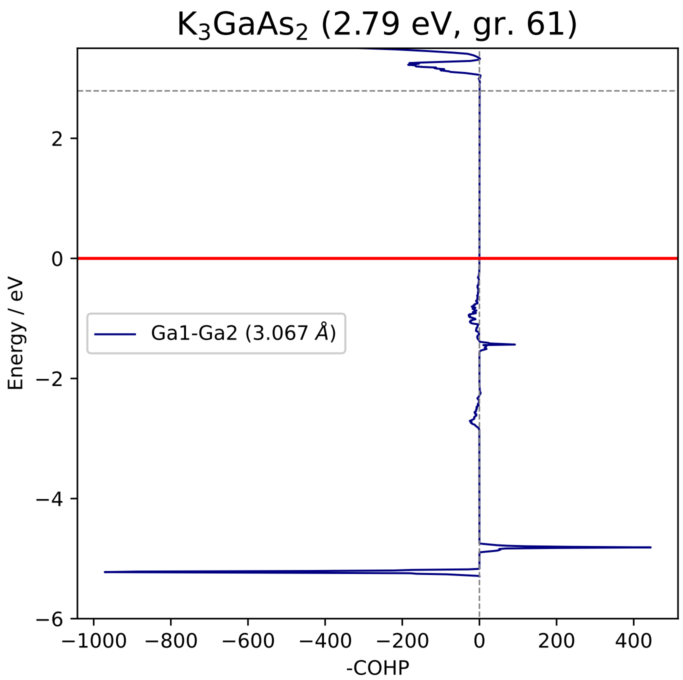

Figure S119. COHP of Ga-Ga interactions in K_3GaAs_2.

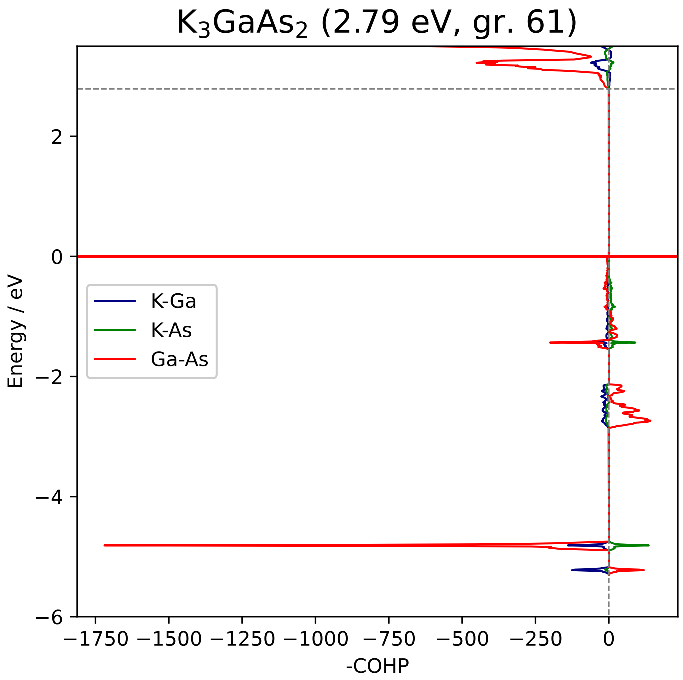

Figure S120. COHP of K-Ga, K-As and Ga-As interactions in K_3GaAs_2.

Table S54. Partial charges for each atom position in K_3GaAs_2.

Atom	Z	charge	part charge
K1	19	18.266	0.734
K2		18.26	0.74
K3		18.248	0.752
K4		18.226	0.774
K5		18.268	0.732
K6		18.262	0.738
Ga1	31	31.183	−0.183

Atom	Z	charge	partialcharge
Ga2		31.104	−0.104
As1	33	33.939	−0.939
As2		34.177	−1.177
As3		33.907	−0.907
As4		34.16	−1.16

10.5 Rb_3AlAs_2

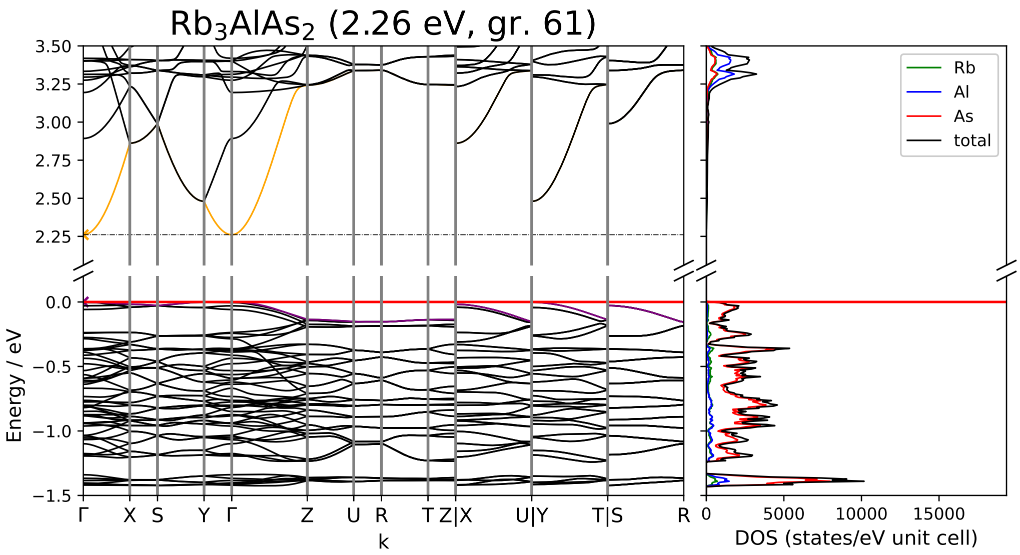

Figure S121. Band structure and DOS of Rb_3AlAs_2 within structure-type **B**.

Table S55. Overlap population and interatomic distances of K_3GaAs_2.

Atom A	Atom B	r_{AB} / Å	overlap
K1	As2	3.327	0.017
	As4	3.361	0.029
	As2	3.403	0.033
	Ga1	3.467	−0.011
	As1	3.475	0.013
	K5	3.585	0.001
	Ga2	3.615	−0.002
	K3	3.617	0.002
	K6	3.831	0.001
	K4	3.938	0.001
	K3	3.953	0.001
	As1	3.991	0.009
K2	As4	3.268	0.026
	As4	3.268	0.031
	As3	3.428	0.017
	As2	3.485	0.025
	Ga1	3.653	−0.007
	K6	3.761	0.001
	Ga2	3.854	0.0
	K3	3.919	0.002
	K4	3.998	0.001
K3	Ga1	3.43	−0.008
	As1	3.443	0.014
	As3	3.449	0.019
	Ga1	3.502	−0.007
	As2	3.562	0.026
	As2	3.619	0.023
	As1	3.884	0.022
	K5	3.963	0.001
K4	As4	3.257	0.03
	As3	3.31	0.015
	Ga1	3.482	−0.001
	Ga2	3.511	−0.005
	As4	3.586	0.02
	Ga2	3.669	−0.001
	As3	3.845	0.009
	As1	3.924	0.008
	K4	3.965	0.001

Atom A	Atom B	r_{AB} / Å	overlap
K5	As2	3.358	0.03
	As2	3.475	0.037
	Ga2	3.499	−0.006
	As3	3.506	0.013
	As1	3.516	0.028
	Ga1	3.622	−0.004
	As1	3.742	0.015
K6	As1	3.529	0.016
	As4	3.534	0.03
	As3	3.537	0.026
	As2	3.623	0.03
	As4	3.705	0.029
	Ga2	3.74	−0.003
Ga1	As2	2.345	0.389
	As3	2.432	0.309
	As1	2.481	0.299
	Ga2	3.067	−0.075
	As1	4.976	0.004
Ga2	As4	2.322	0.439
	As3	2.471	0.309
	As1	2.476	0.284
As1	As3	3.85	−0.077
	As4	4.338	−0.031
	As2	4.378	−0.027
As2	As3	4.267	−0.033
	As3	4.964	0.0
As3	As4	4.316	−0.029
As4	As4	4.942	−0.002

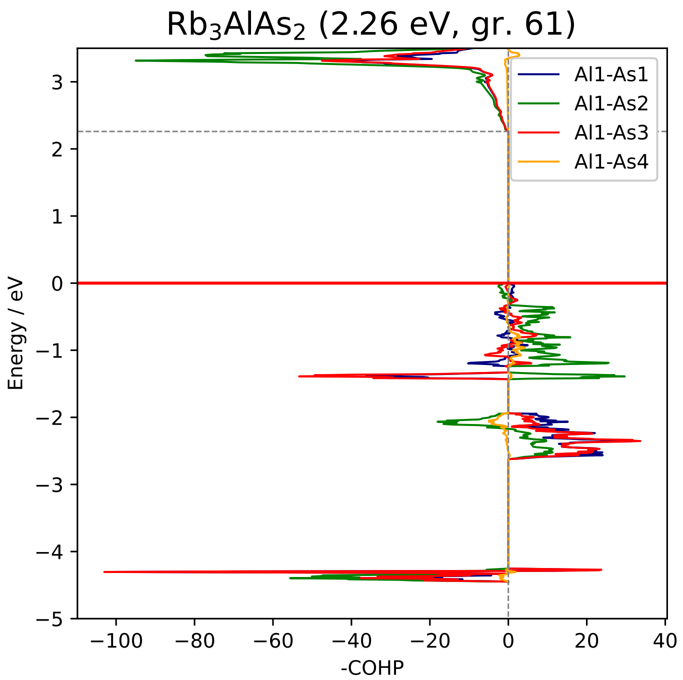

Figure S122. COHP of Al1-As interactions in Rb_3AlAs_2.

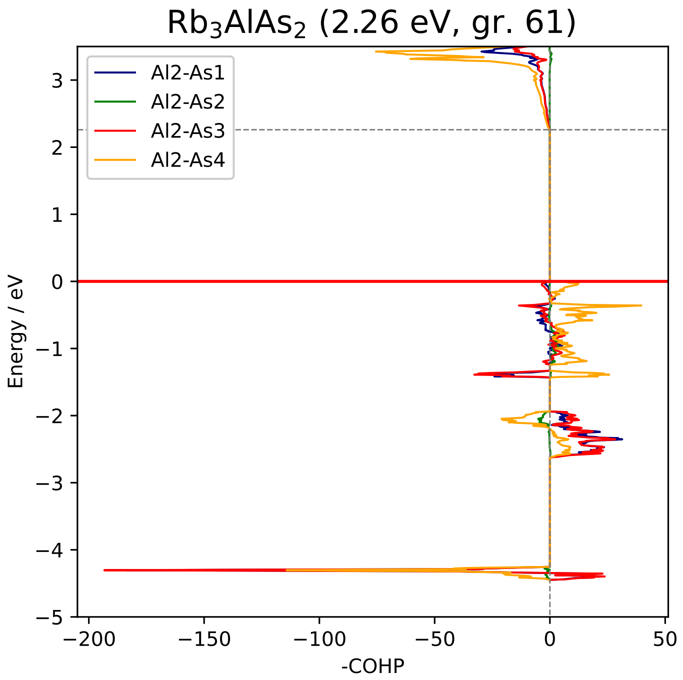

Figure S123. COHP of Al2-As interactions in Rb_3AlAs_2.

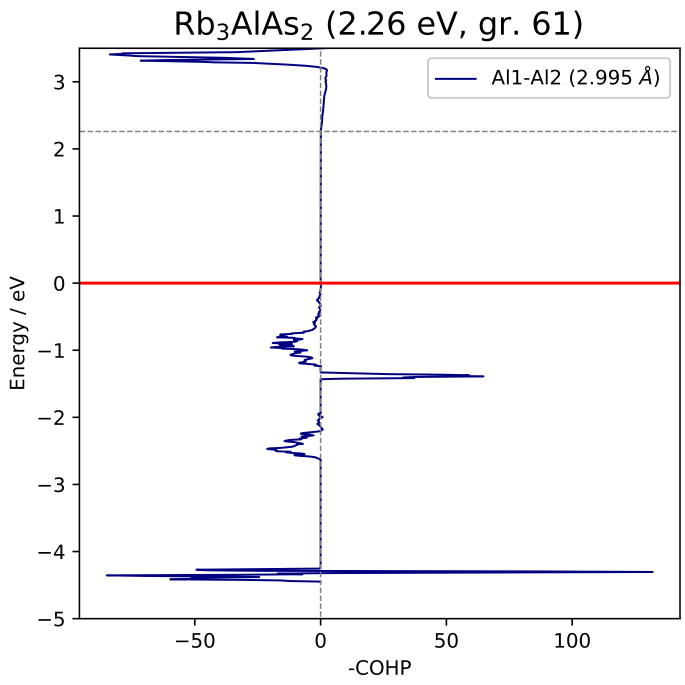

Figure S124. COHP of Al-Al interactions in Rb_3AlAs_2.

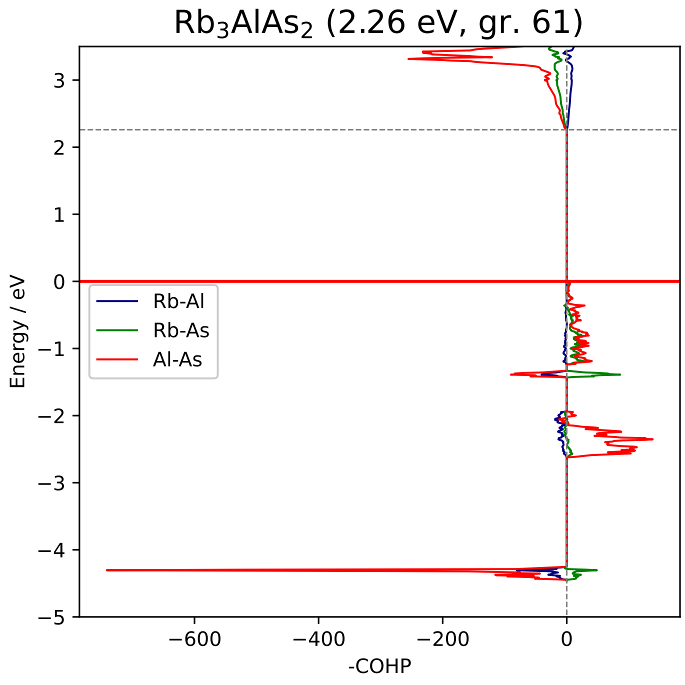

Figure S125. COHP of Rb-Al, Rb-As and Al-As interactions in Rb_3AlAs_2.

Table S56. Partial charges for each atom position in Rb_3AlAs_2.

Atom	Z	charge	part charge
Rb1	9	8.337	0.663
Rb2		8.337	0.663
Rb3		8.32	0.68
Rb4		8.303	0.697
Rb5		8.336	0.664
Rb6		8.323	0.677
Al1	13	12.828	0.172

Atom	Z	charge	partialcharge
Al2		12.802	0.198
As1	33	34.046	−1.046
As2		34.196	−1.196
As3		34.008	−1.008
As4		34.163	−1.163

Table S57. Overlap population and interatomic distances of Rb_3AlAs_2.

Atom A	Atom B	r_{AB} / Å	overlap
Rb1	As2	3.495	0.026
	As4	3.552	0.036
	As1	3.604	0.016
	As2	3.646	0.032
	Al1	3.666	−0.003
	Rb5	3.734	−0.003
	Rb3	3.766	−0.002
	Al2	3.775	0.005
Rb2	As4	3.433	0.036
	As4	3.451	0.036
	As3	3.587	0.02
	As2	3.68	0.03
	Al1	3.864	−0.001
	Rb6	3.932	−0.001
Rb3	As1	3.572	0.019
	As3	3.596	0.02
	Al1	3.622	0.001
	As2	3.679	0.032
	Al1	3.689	0.001
	As2	3.71	0.028
Rb4	As4	3.448	0.036
	As3	3.449	0.019
	As4	3.706	0.029
	Al1	3.713	0.005
	Al2	3.721	0.0
	Al2	3.804	0.005

Atom A	Atom B	r_{AB} / Å	overlap
Rb5	As2	3.555	0.036
	As2	3.656	0.04
	As3	3.661	0.019
	As1	3.711	0.029
	Al2	3.725	0.001
	Al1	3.798	0.001
	As1	3.937	0.018
Rb6	As4	3.647	0.037
	As1	3.666	0.02
	As3	3.709	0.028
	As2	3.82	0.034
	Al2	3.942	0.002
	As4	3.963	0.028
Al1	As2	2.355	0.453
	As3	2.428	0.334
	As1	2.477	0.313
	Al2	2.995	−0.033
Al2	As4	2.327	0.485
	As3	2.459	0.316
	As1	2.467	0.306
As1	As3	3.88	−0.072
	As4	4.315	−0.026
	As2	4.362	−0.024
As2	As3	4.269	−0.027
As3	As4	4.298	−0.025

10.6 Rb_3GaAs_2

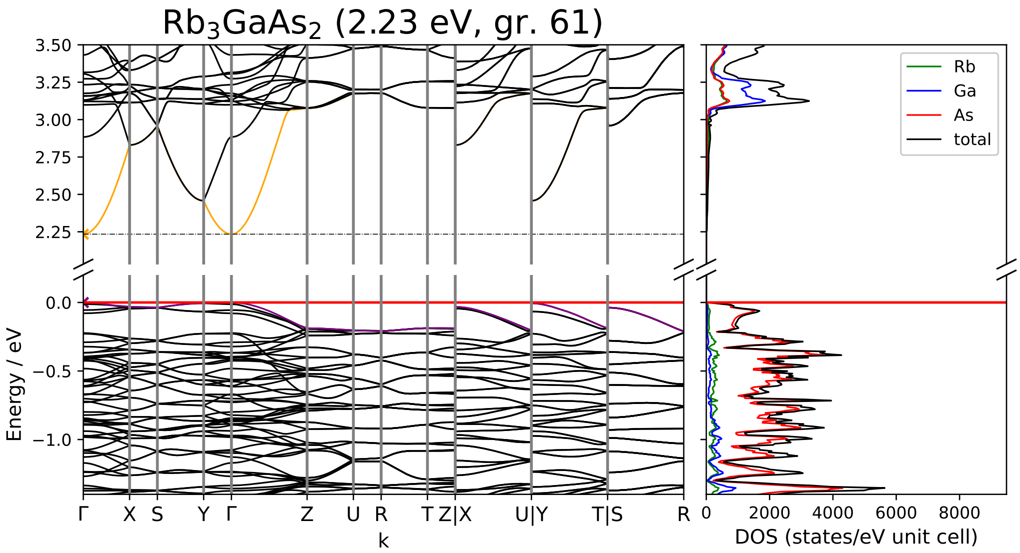

Figure S126. Band structure and DOS of Rb_3GaAs_2 within structure-type **B**.

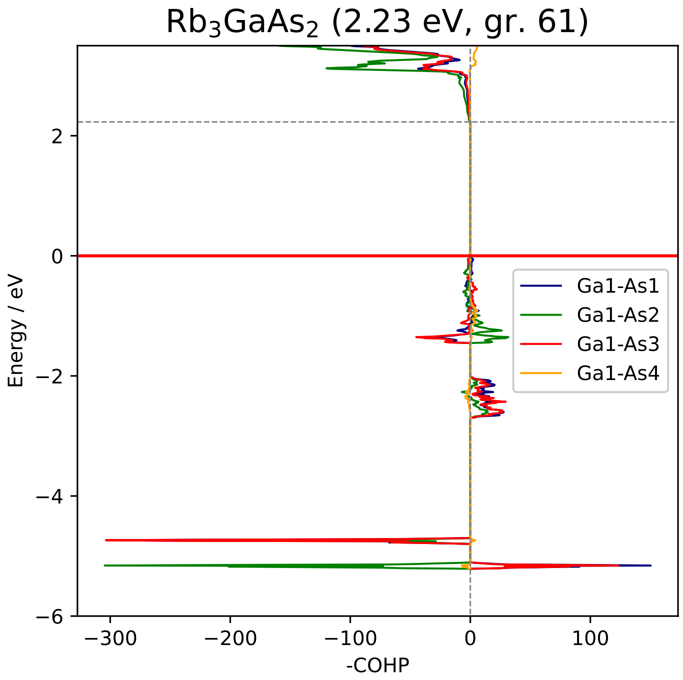

Figure S127. COHP of Ga1-As interactions in Rb_3GaAs_2.

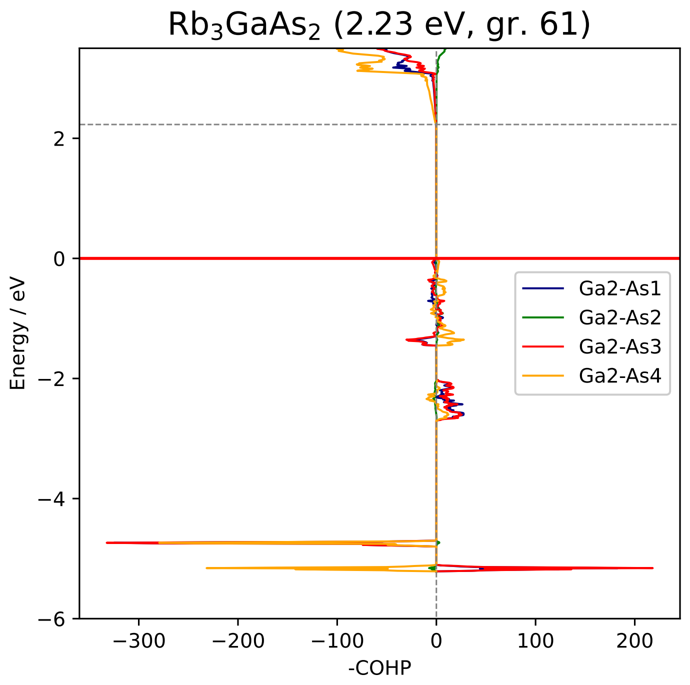

Figure S128. COHP of Ga2-As interactions in Rb_3GaAs_2.

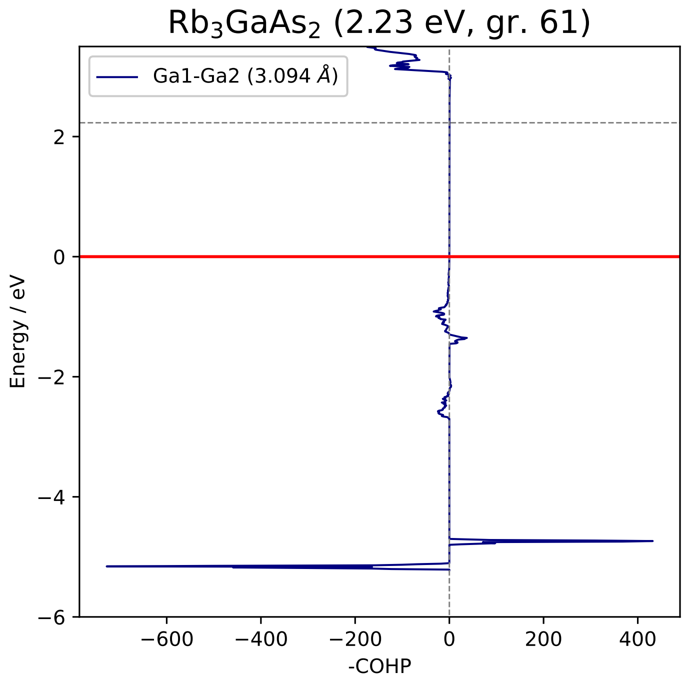

Figure S129. COHP of Ga-Ga interactions in Rb_3GaAs_2.

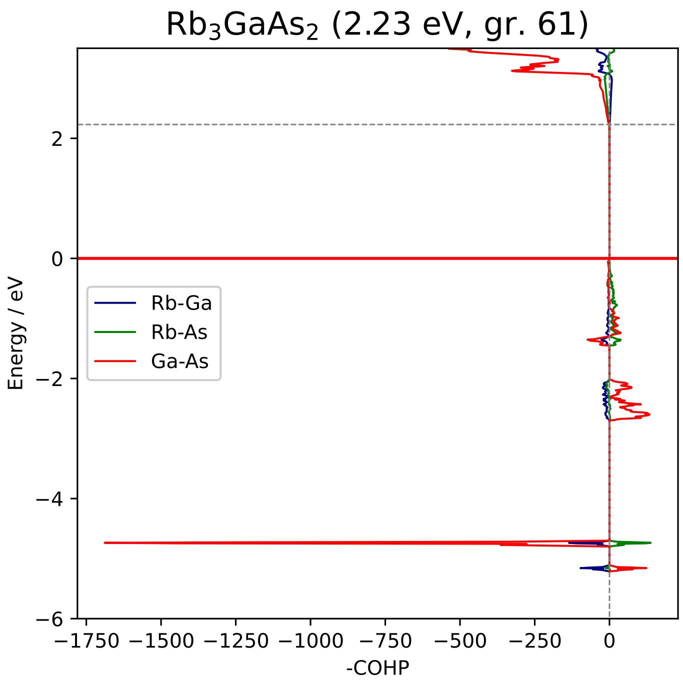

Figure S130. COHP of Rb-Ga, Rb-As and Ga-As interactions in Rb_3GaAs_2.

Table S58. Partial charges for each atom position in Rb_3GaAs_2.

Atom	Z	charge	part charge
Rb1	9	8.325	0.675
Rb2		8.33	0.67
Rb3		8.309	0.691
Rb4		8.285	0.715
Rb5		8.331	0.669
Rb6		8.322	0.678
Ga1	31	31.092	−0.092

Atom	Z	charge	partialcharge
Ga2		31.051	−0.051
As1	33	33.911	−0.911
As2		34.102	−1.102
As3		33.874	−0.874
As4		34.068	−1.068

Table S59. Overlap population and interatomic distances of Rb_3GaAs_2.

Atom A	Atom B	r_{AB} / Å	overlap
Rb1	As2	3.485	0.023
	As4	3.543	0.035
	As1	3.607	0.016
	As2	3.625	0.033
	Ga1	3.627	−0.01
	Rb5	3.755	−0.002
	Ga2	3.76	−0.001
	Rb3	3.776	−0.001
Rb2	As4	3.424	0.036
	As4	3.449	0.035
	As3	3.574	0.021
	As2	3.668	0.029
	Ga1	3.841	−0.007
	Rb6	3.956	−0.001
Rb3	As1	3.569	0.018
	Ga1	3.597	−0.007
	As3	3.613	0.019
	Ga1	3.67	−0.006
	As2	3.682	0.032
	As2	3.739	0.026
Rb4	As4	3.446	0.035
	As3	3.457	0.02
	Ga1	3.677	−0.001
	Ga2	3.701	−0.005
	As4	3.713	0.026
	Ga2	3.797	0.0

Atom A	Atom B	r_{AB} / Å	overlap
Rb5	As2	3.542	0.035
	As3	3.638	0.017
	As2	3.66	0.04
	Ga2	3.697	−0.004
	As1	3.728	0.031
	Ga1	3.788	−0.003
	As1	3.889	0.018
Rb6	As4	3.648	0.036
	As1	3.672	0.02
	As3	3.719	0.029
	As2	3.805	0.034
	Ga2	3.912	−0.004
	As4	3.957	0.028
Ga1	As2	2.357	0.397
	As3	2.439	0.316
	As1	2.492	0.302
	Ga2	3.094	−0.07
Ga2	As4	2.329	0.428
	As3	2.474	0.311
	As1	2.483	0.293
As1	As3	3.84	−0.073
	As4	4.352	−0.028
	As2	4.406	−0.024
As2	As3	4.294	−0.03
As3	As4	4.334	−0.027

10.7 Rb_3InAs_2

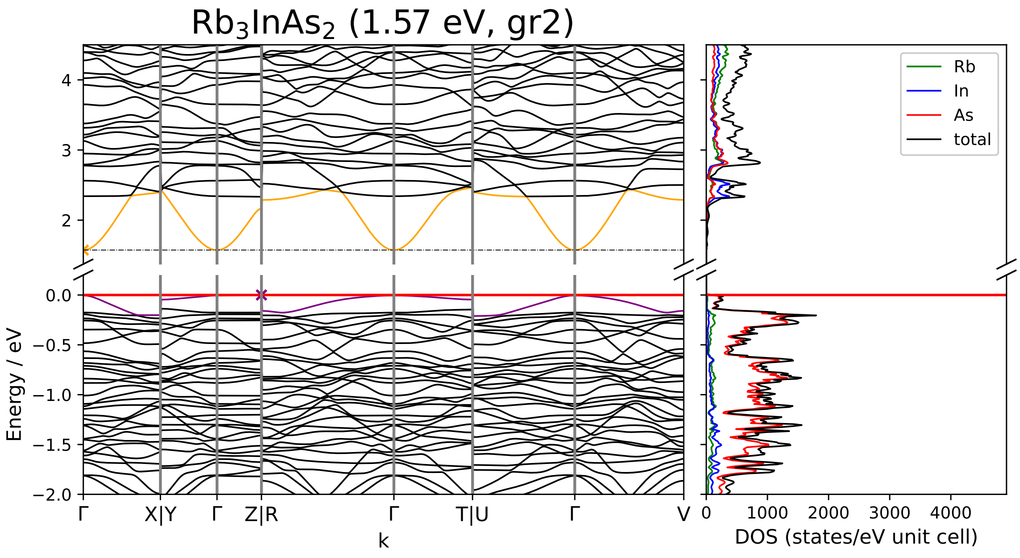

Figure S131. Band structure and DOS of Rb_3InAs_2 within structure-type **C**.

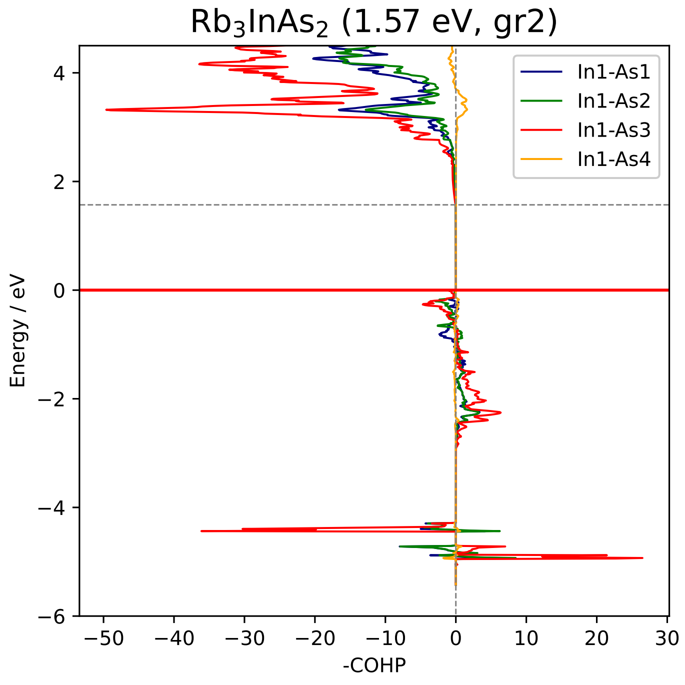

Figure S132. COHP of In1-As interactions in Rb_3InAs_2.

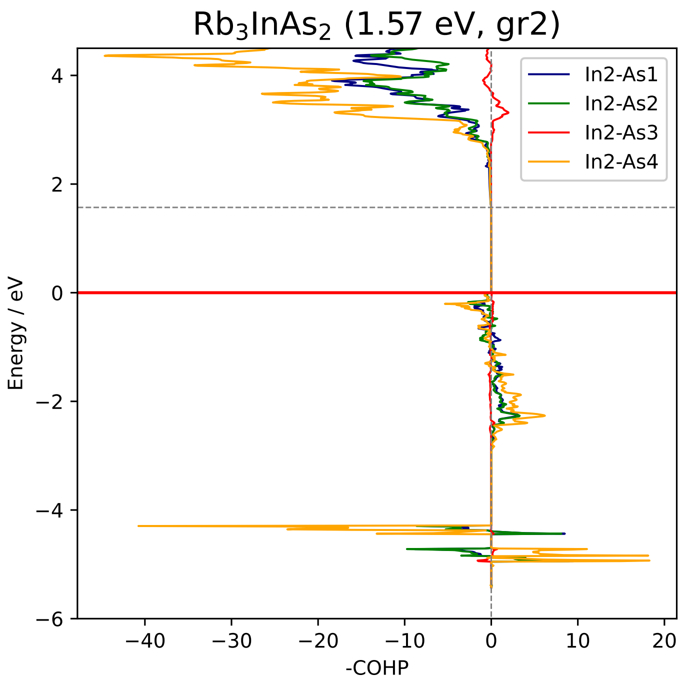

Figure S133. COHP of In2-As interactions in Rb_3InAs_2.

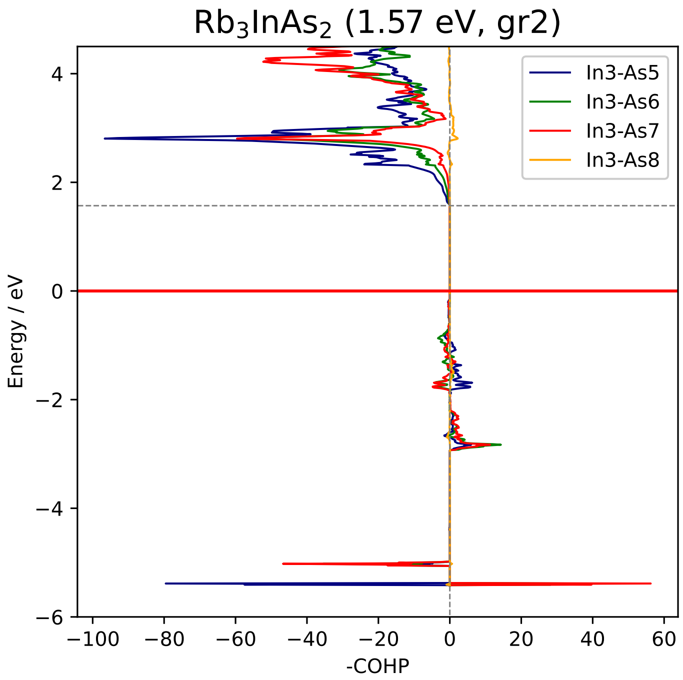

Figure S134. COHP of In3-As interactions in Rb_3InAs_2.

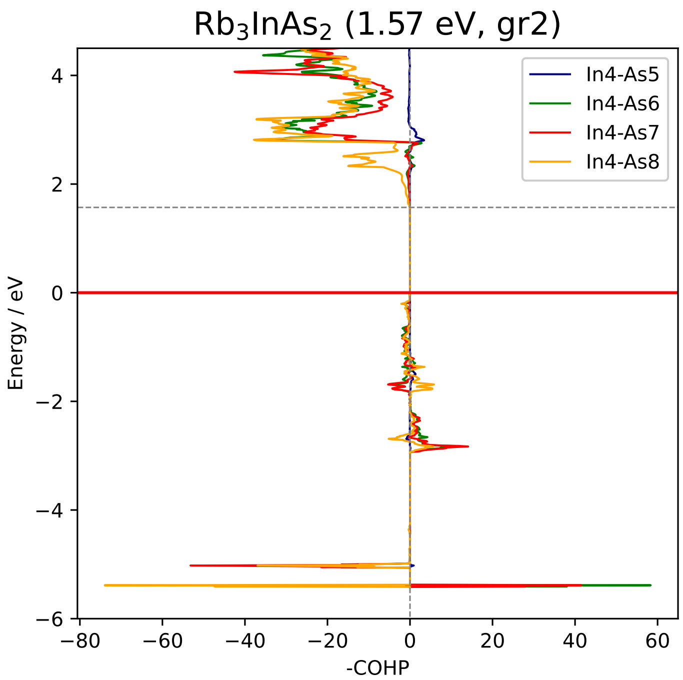

Figure S135. COHP of In4-As interactions in Rb_3InAs_2.

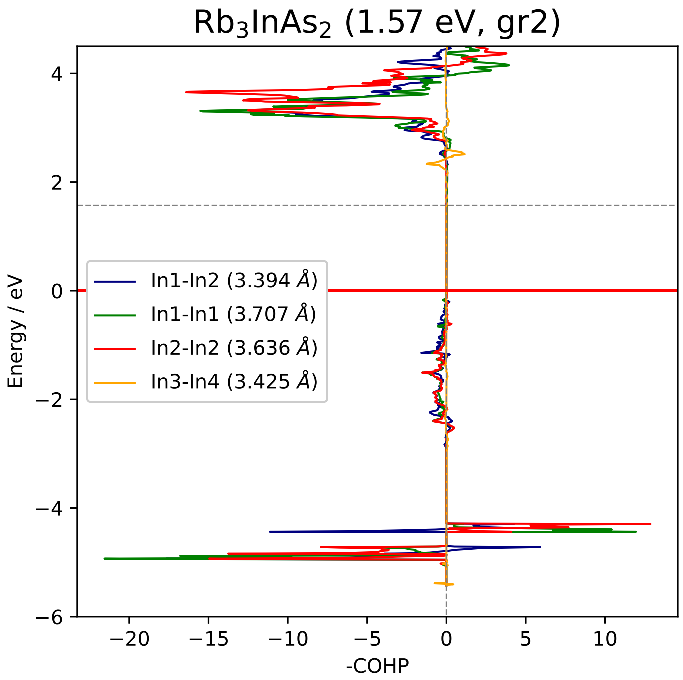

Figure S136. COHP of In-In interactions in Rb_3InAs_2.

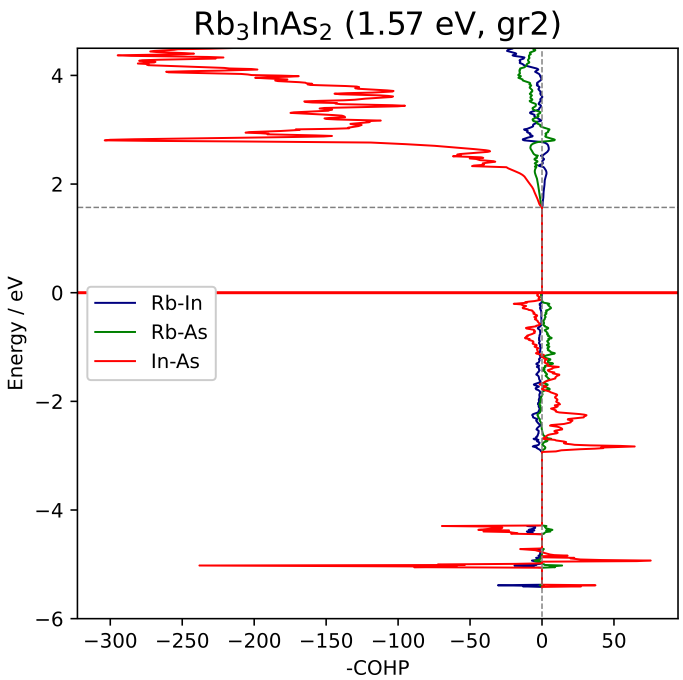

Figure S137. COHP of Rb-In, Rb-As and In-As interactions in Rb_3InAs_2.

10.8 Cs_3InAs_2

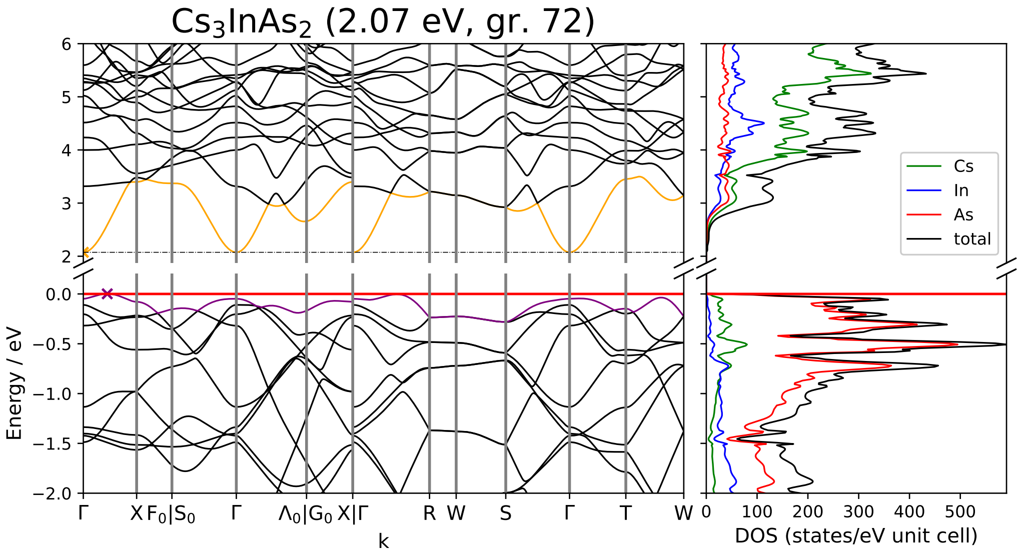

Figure S138. Band structure and DOS of Cs_3InAs_2 within structure-type **E**.

Table S60. Overlap population and interatomic distances of Rb_3InAs_2.

Atom A	Atom B	r_{AB} / Å	overlap	Atom A	Atom B	r_{AB} / Å	overlap
Rb1	Rb2	3.405	−0.011	Rb9	As8	3.434	0.03
	As7	3.406	0.007		As2	3.442	0.029
	In1	3.514	−0.016		As6	3.658	0.034
	As2	3.58	0.015		As4	3.669	0.038
	In1	3.586	−0.012		Rb10	3.838	−0.001
	Rb8	3.638	−0.005	Rb10	Rb10	3.646	−0.003
	As3	3.654	0.012		As2	3.681	0.022
	As3	3.656	0.01		As1	3.697	0.022
	As5	3.757	0.017		As4	3.713	0.034
	In3	3.783	−0.013		As1	3.863	0.024
Rb2	As4	3.322	0.035		As8	3.88	0.023
	As1	3.35	0.024		In2	3.997	−0.002
	As3	3.522	0.03	Rb11	As5	3.438	0.038
	As5	3.568	0.032		Rb11	3.525	−0.007
	Rb5	3.836	−0.001		As7	3.573	0.033
	In1	3.946	−0.009		Rb12	3.594	−0.005
	Rb10	3.998	0.0		As8	3.617	0.035
Rb3	In2	3.602	−0.009		As7	3.78	0.014
	As1	3.654	0.017		In4	3.991	−0.005
	As4	3.686	0.017	Rb12	As6	3.457	0.039
	In2	3.703	−0.007		As5	3.597	0.033
	As8	3.8	0.023		As8	3.648	0.038
	As4	3.83	0.015		As6	3.816	0.014
	Rb9	3.837	−0.001		In3	3.818	−0.007
	As6	3.885	0.013	In1	As2	2.715	0.257
	As2	3.891	0.019		As1	2.733	0.249
	Rb10	3.917	0.0		As3	2.734	0.263
Rb4	As8	3.541	0.032		As3	2.788	0.239
	As1	3.595	0.048		In2	3.394	−0.035
	In3	3.717	−0.003		In1	3.707	−0.048
	In4	3.851	−0.004	In2	As2	2.708	0.252
	As7	3.873	0.016		As4	2.719	0.252
Rb5	As1	3.472	0.029		As4	2.72	0.257
	As3	3.481	0.033		As1	2.732	0.249
	As4	3.649	0.032		In2	3.636	−0.04
	As5	3.651	0.03	In3	As5	2.509	0.385
	In1	3.893	−0.009		As6	2.641	0.281
Rb6	As3	3.506	0.034		As7	2.674	0.267
	As2	3.553	0.03		In4	3.425	−0.072
	As4	3.763	0.032	In4	As8	2.477	0.35
	As3	3.886	0.034		As7	2.6	0.277
Rb7	As2	3.44	0.049		As6	2.669	0.257
	As5	3.709	0.028	As1	As2	4.108	−0.035
	As6	3.762	0.02	As3	As3	4.093	−0.044
	In3	3.813	−0.003	As4	As4	4.044	−0.04
	As7	3.89	0.019	As5	As7	4.623	−0.018
	In4	3.937	−0.001	As6	As7	4.029	−0.062
Rb8	As8	3.422	0.038	As7	As8	4.585	−0.019
	As3	3.542	0.038				
	As7	3.587	0.029				
	As2	3.733	0.028				
	Rb10	3.915	0.0				

Table S61. Partial charges for each atom position in Rb_3InAs_2.

Atom	Z	charge	part charge
Rb1	9	8.283	0.717
Rb2		8.362	0.638
Rb3		8.293	0.707
Rb4		8.315	0.685
Rb5		8.332	0.668
Rb6		8.36	0.64
Rb7		8.32	0.68
Rb8		8.349	0.651
Rb9		8.331	0.669
Rb10		8.317	0.683
Rb11		8.327	0.673
Rb12		8.327	0.673
In1	21	20.857	0.143

Atom	Z	charge	partialcharge
In2		20.802	0.198
In3		21.081	−0.081
In4		21.067	−0.067
As1	33	34.069	−1.069
As2		34.072	−1.072
As3		34.077	−1.077
As4		34.068	−1.068
As5		34.063	−1.063
As6		33.947	−0.947
As7		33.936	−0.936
As8		34.043	−1.043

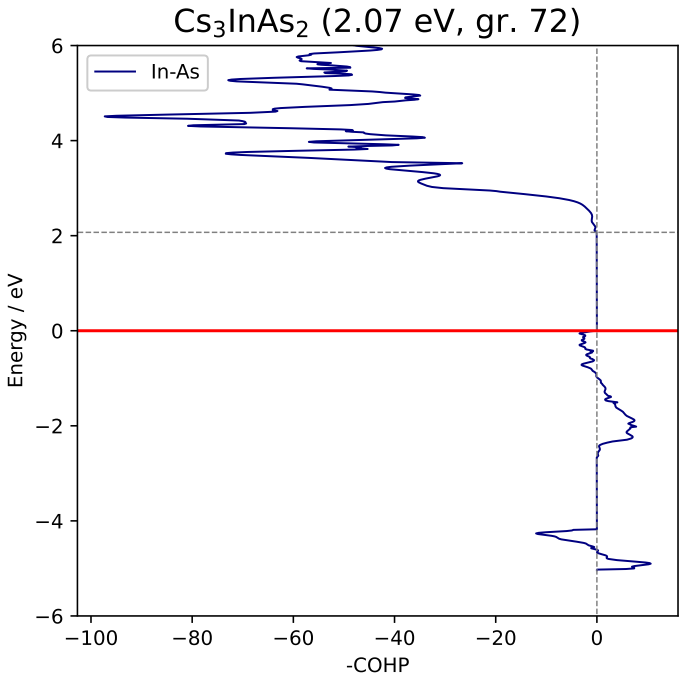

Figure S139. COHP of In-As interactions in Cs_3InAs_2.

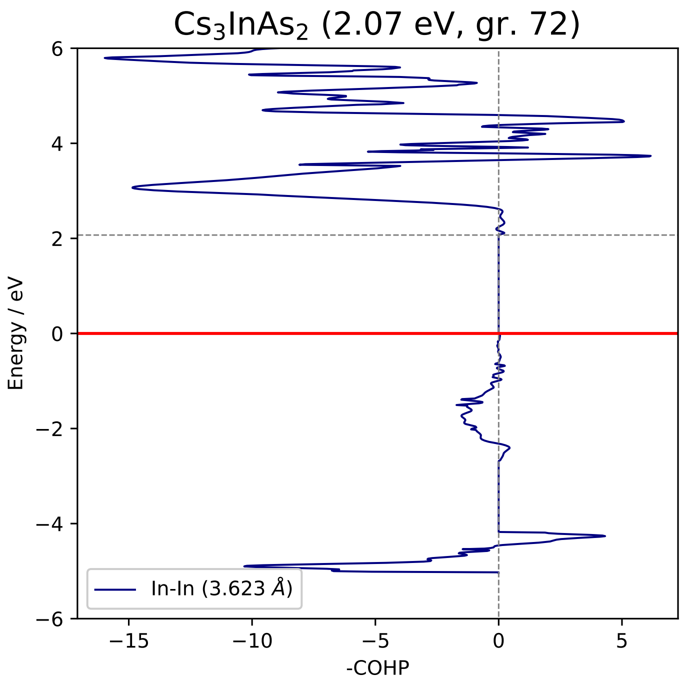

Figure S140. COHP of In-In interactions in Cs_3InAs_2.

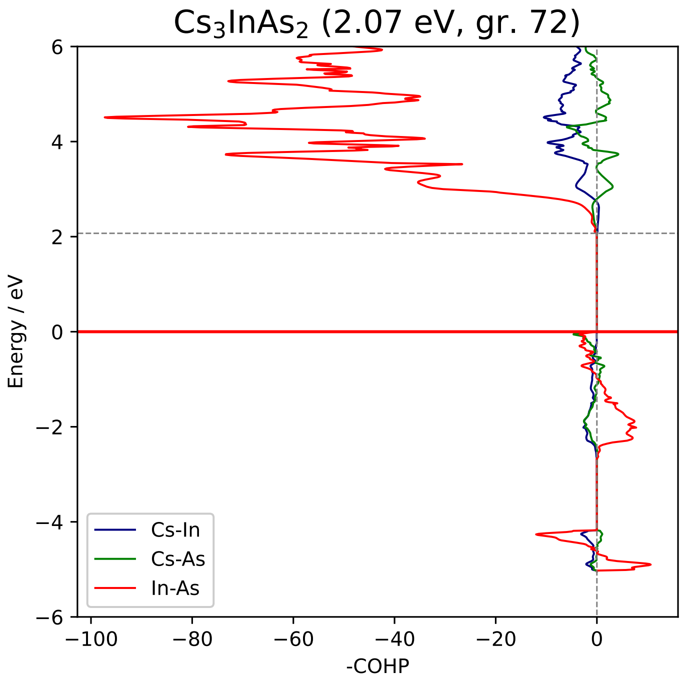

Figure S141. COHP of Cs-In, Cs-As and In-As interactions in Cs_3InAs_2.

Table S62. Partial charges for each atom position in Cs_3InAs_2.

Atom	Z	charge	part charge
Cs1	9	8.294	0.706
Cs2		8.354	0.646
In1	21	20.852	0.148

Atom	Z	charge	partialcharge
As1	33	34.102	−1.102

Table S63. Overlap population and interatomic distances of Cs_3InAs_2.

Atom A	Atom B	r_{AB} / Å	overlap
Cs1	As1	3.522	0.018
	In1	3.762	−0.005
	As1	3.776	0.04
	As1	3.878	0.018
	Cs2	4.155	0.006
	As1	4.236	0.008
Cs2	As1	3.581	0.028
	Cs2	3.623	0.005
	In1	4.235	−0.004
	In1	5.573	−0.001

Atom A	Atom B	r_{AB} / Å	overlap
In1	As1	2.754	0.255
	In1	3.623	−0.042
	As1	5.817	−0.001
As1	As1	4.149	−0.042

References

(1) Restle, T. M.; Dums, J. V.; Raudaschl-Sieber, G.; Fässler, T. F. *Chemistry–A European Journal* **2020**, *26*, 6812–6819.

(2) Wegner, F.; Kamm, F.; Pielnhofer, F.; Pfitzner, A. *Zeitschrift für anorganische und allgemeine Chemie* **2022**, *649*, e202200330.

(3) Restle, T. M.; Deringer, V. L.; Meyer, J.; Raudaschl-Sieber, G.; Fässler, T. F. *Chemical Science* **2021**, *12*, 1278–1285.

(4) Ohse, L.; Somer, M.; Blase, W.; Cordier, G. *Zeitschrift für Naturforschung B* **1993**, *48*, 1027–1034.

(5) Restle, T. M.; Zeitz, S.; Meyer, J.; Klein, W.; Raudaschl-Sieber, G.; Karttunen, A. J.; Fässler, T. F. *Zeitschrift für anorganische und allgemeine Chemie* **2021**, *647*, 1804–1814.

(6) Cordier, G; Ochmann, H *Zeitschrift für Naturforschung. B, A journal of chemical sciences* **1988**, *43*, 1538–1540.

(7) Boyko, M.; Hlukhyy, V.; Fässler, T. F. *Zeitschrift für anorganische und allgemeine Chemie* **2023**, *649*, e202300164.

(8) Blase, W; Cordier, G; Somer, M *Zeitschrift für Kristallographie-Crystalline Materials* **1991**, *195*, 119–120.

(9) Cordier, G; Ochmann, H *Zeitschrift für Kristallographie-Crystalline Materials* **1991**, *195*, 105–106.

(10) Somer, M; Walz, L; Peters, K; Schnering, H. v. *Zeitschrift für Kristallographie-Crystalline Materials* **1990**, *193*, 301–302.

(11) Somer, M; Carrillo-Cabrera, W; Peters, E.-M.; Peters, K; Schnering, H. v. *Zeitschrift für Kristallographie-New Crystal Structures* **1998**, *213*, 5–6.

(12) Von Schnering, H.; Somer, M; Walz, L; Peters, K; Cordier, G; Blase, W *Zeitschrift für Kristallographie-Crystalline Materials* **1990**, *193*, 299–300.

(13) Blase, W; Cordier, G; Somer, M *Zeitschrift für Kristallographie-Crystalline Materials* **1991**, *195*, 123–124.

(14) Somer, M; Peters, K; Thiery, D; von Schnering, H. *Zeitschrift für Kristallographie* **1990**, *192*, 271–272.

(15) Somer, M; Walz, L; Thiery, D; Schnering, H. v. *Zeitschrift für Kristallographie-Crystalline Materials* **1990**, *193*, 303–304.

(16) Somer, M; Thiery, D; Hartweg, M; Walz, L; Peters, K; von Schnering, H. *Zeitschrift für Kristallographie-Crystalline Materials* **1990**, *193*, 287–288.

(17) Somer, M; Thiery, D; Hartweg, M; Walz, L; Popp, T; Peters, K; von Schnering, H. *Zeitschrift für Kristallographie* **1990**, *192*, 269–270.

(18) Somer, M; Peters, K; Popp, T; Schnering, H. v. *Zeitschrift für Kristallographie-Crystalline Materials* **1990**, *192*, 273–274.
